# Genome-wide association analysis for quantitative trait loci influencing Warner–Bratzler shear force in five taurine cattle breeds

**DOI:** 10.1111/j.1365-2052.2012.02323.x

**Published:** 2012-02-27

**Authors:** M C McClure, H R Ramey, M M Rolf, S D McKay, J E Decker, R H Chapple, J W Kim, T M Taxis, R L Weaber, R D Schnabel, J F Taylor

**Affiliations:** * Division of Animal Sciences, University of MissouriColumbia, MO, 65211, USA; † Bovine Functional Genomics LaboratoryARS, USDA, Beltsville, MD, 20705, USA

**Keywords:** beef, *Bos taurus taurus*, calpain 1, (mu/I) large subunit, calpastatin, genome-wide association, haplotype, meat tenderness, quantitative trait loci, single-nucleotide polymorphisms, Warner–Bratzler shear force

## Abstract

We performed a genome-wide association study for Warner–Bratzler shear force (WBSF), a measure of meat tenderness, by genotyping 3360 animals from five breeds with 54 790 BovineSNP50 and 96 putative single-nucleotide polymorphisms (SNPs) within *μ-calpain* [HUGO nomenclature *calpain 1, (mu/I) large subunit*; *CAPN1*] and *calpastatin* (*CAST*). Within- and across-breed analyses estimated SNP allele substitution effects (ASEs) by genomic best linear unbiased prediction (GBLUP) and variance components by restricted maximum likelihood under an animal model incorporating a genomic relationship matrix. GBLUP estimates of ASEs from the across-breed analysis were moderately correlated (0.31–0.66) with those from the individual within-breed analyses, indicating that prediction equations for molecular estimates of breeding value developed from across-breed analyses should be effective for genomic selection within breeds. We identified 79 genomic regions associated with WBSF in at least three breeds, but only eight were detected in all five breeds, suggesting that the within-breed analyses were underpowered, that different quantitative trait loci (QTL) underlie variation between breeds or that the BovineSNP50 SNP density is insufficient to detect common QTL among breeds. In the across-breed analysis, *CAPN1* was followed by *CAST* as the most strongly associated WBSF QTL genome-wide, and associations with both were detected in all five breeds. We show that none of the four commercialized *CAST* and *CAPN1*SNP diagnostics are causal for associations with WBSF, and we putatively fine-map the *CAPN1* causal mutation to a 4581-bp region. We estimate that variation in *CAST* and *CAPN1* explains 1.02 and 1.85% of the phenotypic variation in WBSF respectively.

## Introduction

Consumer assessment of beef quality, palatability and overall eating satisfaction is significantly influenced by tenderness (Huffman *et al*. 1996; Weston *et al*. 2002; Moser *et al*. 2004; Smith *et al*. 2006), and consumers have indicated a willingness to pay a premium for ‘guaranteed tender' steak (Boleman *et al*. 1997; Mintert *et al*. 2000; Miller *et al*. 2001; Platter *et al*. 2005). Inadequate tenderness has consistently been identified in National Beef Quality Audits as a priority quality challenge (Lorenzen *et al*. 1993; Roeber *et al*. 2000; Shook *et al*. 2008) because consumers consider tenderness to be the single most important component of meat quality and will substitute protein sources motivated by their dissatisfaction from the purchase of a tough cut (Miller *et al*. 1995; McKenna *et al*. 2002).

To address these concerns, researchers have identified quantitative trait loci (QTL) for Warner–Bratzler shear force (WBSF) measurements on the longissimus dorsi muscle on chromosomes 2, 4, 5, 7, 10, 11, 15, 20, 25 and 29 (Casas *et al*. 1998, 2000, 2001, 2003; Keele *et al*. 1999; Rexroad *et al*. 2001; Alexander *et al*. 2007; Davis *et al*. 2008; Gutierrez-Gil *et al*. 2008; Gill *et al*. 2009, 2010). However, from these reported QTL, DNA marker tests have been developed and commercialized only from *calpastatin* (*CAST*) on chromosome 7 and *calpain 1, (mu/I) large subunit* (*CAPN1*) on chromosome 29 (Page *et al*. 2002, 2004; White *et al*. 2005; Casas *et al*. 2006; Van Eenennaam *et al*. 2007). While these commercialized marker tests are predictive of tenderness in both *Bos taurus taurus* and *B. t. indicus* breeds, it appears that they are not causal for the detected associations with tenderness (Casas *et al*. 2003). However, the estimated genotypic associations estimated for these markers are large, with an average difference of 0.15 kg in WBSF between alternate homozygotes in independent studies involving several breeds (Casas *et al*. 2006; Morris *et al*. 2006; Van Eenennaam *et al*. 2007; Johnston & Graser 2010). While positional candidate genes on other chromosomes have been investigated (Rexroad *et al*. 2001; Stone *et al*. 2005), none have resulted in commercial tests.

To assist beef breeders to make efficient and large changes in tenderness, DNA assays must be developed that can reliably predict the genetic variation in tenderness without regard to the breed composition of an animal. To address this need, we genotyped 3360 animals representing 114 half-sib families produced by the American Angus Association (AAA), American Hereford Association (AHA), American Simmental Association (ASA), American International Charolais Association (AICA) and the North American Limousin Foundation (NALF) as part of the National Cattlemen's Beef Association (NCBA) sponsored Carcass Merit Project (CMP) to develop prediction equations for the implementation of genomic selection (Meuwissen *et al*. 2001) and to identify genomic regions associated with tenderness. This study reports genomic regions detected as being concordant across breeds, which putatively harbour candidate genes that influence tenderness and which could be targeted for the development of diagnostic assays. We also dissect variation within *CAST* and *CAPN1* in order to identify the genomic regions most likely to harbour the causal variants influencing beef tenderness.

## Materials and methods

### Animals and phenotype

A total of 3360 animals representing five of the breed associations participating in the NCBA-sponsored CMP were selected for genotyping based on the availability of WBSF data and DNA samples (Table 1). The design of the CMP project has previously been described by Minick *et al*. (2004); however, only the Angus and Hereford samples represent purebred populations, with the Continental breeds being represented by crossbred progeny, with Simmental, Charolais and Limousin sires mated to predominantly commercial Angus cows. Meat tenderness was measured as WBSF (kg) of longissimus dorsi steaks at day 14 post-mortem as previously described (Wheeler *et al*. 1998; Minick *et al*. 2004). Muscle samples, extracted DNA samples and carcass phenotypes produced in the CMP and owned by the AAA, AHA, ASA, AICA and NALF were transferred to the University of Missouri. All CMP animals had blood samples drawn at weaning, from which DNA was extracted and tested to validate the identity of their sires. Additionally, a muscle sample was taken at slaughter at the capture of phenotype data on most of the animals, and DNA extracted from a subset of the muscle samples was previously genotyped and compared with the genotype profiles produced from the corresponding blood samples to validate the identity of each carcass. This process identified that about 10% of animals or carcasses were misidentified (Thallman *et al*. 2003) likely due to changes in the order of carcasses because of ‘rail-outs' at packing plants. To resolve this issue, we extracted genomic DNA from 2940 muscle samples taken from the phenotyped carcasses by proteinase K digestion followed by phenol–chloroform–isoamyl alcohol extraction and ethanol precipitation (Sambrook *et al*. 1989). The remaining 420 DNA samples were extracted from the blood, but these samples had previously been DNA-typed and successfully matched to the sample taken at harvest.

**Table 1:** Animal counts, mean phenotype and estimates of additive genetic variance and heritability by breed.

Breed	Count		Warner–Bratzler shear force (kg)		
Animals^1^	Sires	Average	$\sigma_{A}^{2}$	h^2^	
Angus	660 (651)	20	3.74	0.22	0.52
Charolais	702 (695)	18	4.41	0.23	0.46
Hereford	1192 (1095)	29	4.75	0.15	0.17
Limousin	285 (283)	23	4.28	0.07	0.09
Simmental	521 (516)	24	4.36	0.06	0.08
All Breeds	3360 (3240)	114	4.37	0.17	0.25

Numbers of animals with genotype call rate ≥0.85 in parentheses.

### Genotypes

All samples were genotyped using the Illumina BovineSNP50 BeadArray (Matukumalli *et al*. 2009) for 54 790 single-nucleotide polymorphisms (SNPs) and a custom-designed Illumina GoldenGate assay incorporating 96 putative SNPs located within 186 kb of *CAST* and *CAPN1* (White *et al*. 2005; Casas *et al*. 2006). Several of the putative SNPs identified in the genome sequencing project were not variable (Table S1), and we were much more successful in fine-mapping *CAPN1* than *CAST*. All genotypes were called in the Illumina genomestudio software. Genotypes were filtered according to their unique localization to an autosome or the X chromosome in the University of Maryland sequence assembly (UMD3.0; Zimin *et al*. 2009), call rate (>0.89) and minor allele frequency >0.01 within each breed. Animals were excluded if their individual genotype call rate was <0.85. The call rate of >0.89 for SNP filtering was used to ensure that all commercialized tenderness SNPs were included in the analysis. After filtering, the data set comprised 40 645 SNPs assayed in 3240 animals (Tables 1 Table S1), discovered either as part of the bovine genome sequencing project or through directed *CAPN1* resequencing studies at the US Meat Animal Research Center at Clay Center, NE (Page *et al*. and S2).

### Analysis

fastphase v1.2.3 (Scheet & Stephens 2006) was used with UMD3.0 coordinates to phase all genotypes and impute the 0.89% of missing genotypes. The complete set of genotypes was then used to generate a genomic relationship matrix (G) across all breeds using the first of the methods described by VanRaden (2008) with a modification allowing the inclusion of X-linked loci as described below.

Warner–Bratzler shear force phenotypes were analysed under a single-trait mixed linear animal model in which the genomic relationship matrix was used to represent the realized identity by descent among the animals. The model fit was *y* = *X*β + Z*u* + *e* where *y* is a vector of WBSF measurements, *β* is a vector of fixed contemporary group effects defined as breed × herd of origin × sex of calf × slaughter date, *u* is a vector of random additive genetic merits, and *e* is a vector of random residuals. The matrices *X* and *Z* are incidence matrices relating observations to levels of the fixed and random effects, and we assume that $\mathrm{Var}(u)=G\sigma_{A}^{2},\mathrm{Var}(e)=I\sigma_{E}^{2}$ and Cov (u,e) = 0. Restricted maximum likelihood was used to estimate the variance components $\sigma_{A}^{2}$ and $\sigma_{E}^{2}$ and iteration on the variance component estimates continued until the estimate of heritability $h^{2}=\sigma_{A}^{2}/(\sigma_{A}^{2}+\sigma_{E}^{2})$ had converged to four significant figures. At convergence, the GBLUP of the vector of SNP allele substitution effects (ASEs) was obtained as $\hat{a}={\left(2\Sigma_{i}p_{i}q_{i}\right)}^{-1}M'G^{-1}\hat{u}\;\;$ where *p*_*i*_ is the frequency of the *A* allele at the *i*th SNP (genotypes at each SNP are called in *A*/*B* space by the GenomeStudio software), *q*_*i*_ = 1 – *p*_*i*_, elements of the *i*th column of M are 2*q*_*i*_, *q*_*i*_ − *p*_*i*_ and −2*p*_*i*_ for *AA*, *AB* and *BB* genotypes at autosomal and pseudoautosomal loci (VanRaden 2008) and are *q*_*i*_ and –*p*_*i*_ for *AY* and *BY* genotypes at X-linked loci in males, and $\hat{u}$ is GBLUP of *u*. Analyses were performed both within each breed and across all breeds.

**Figure 1:**
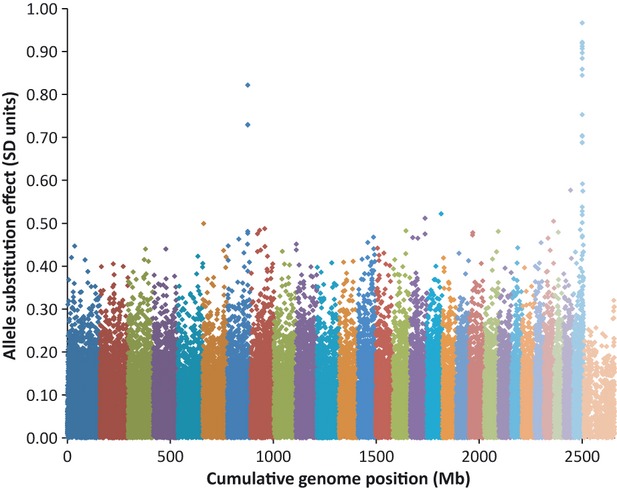
Manhattan plot of single-nucleotide polymorphism (SNP) allele substitution effects estimated in the across-breed analysis and normalized by the square root of the estimated SNP variance component.

The variance component associated with SNP ASEs is $\sigma_{M}^{2}={\left(2\Sigma_{i}p_{i}q_{i}\right)}^{-1}\sigma_{A}^{2}\;$, and for each SNP, the predicted ASE was normalized to a *t*-like statistic as *t*_*i*_ = |α_*i*_|/σ_*M*_. These values are included in Table S2 and are shown in the Manhattan plots in Figs 1 and S1.

### Across-breed comparison of putative QTL regions

To determine whether common QTL influence WBSF across breeds, we ranked the *t*_*i*_ values estimated in the within- and across-breed analyses and then identified SNPs for which the *t*_*i*_ values ranked in the top 500 (1.2%) of SNP ASEs in the across-breed analysis. For each of the regions tagged by these SNPs, we declared the region to harbour a QTL if at least three SNPs from different within-breed analyses had ASEs ranked in the top 500. While linkage disequilibrium (LD) decays to ∼0.1 within less than a 500-kb distance within breeds of distantly related individuals (McKay *et al*. 2007), many of the individuals incorporated into these analyses are half-sibs (Table 1), which leads to a much greater extent of LD because of large common chromosomal segments transmitted by the sires to their progeny. Additionally, we wanted to allow for the possibility that more than one QTL could be present within any one genomic region. Accordingly, we allowed the region size to vary up to 5.7 Mb (average 1.7 Mb) as determined by the signatures of the detected within-breed SNP ASE ranks. Furthermore, within each region, we did not expect to find the same SNP to be most strongly associated with WBSF, because differences in SNP and QTL allele frequencies between breeds (Table S2) can lead to different patterns of LD in different breeds.

### Candidate genes

Genomic regions identified as being associated with WBSF in at least four breeds were analysed using the NCBI Entrez Map Viewer (accessed 07/06/2011) to identify potential candidate genes for tenderness.

### *CAST* and *CAPN1*

A 1.48-Mb region of BTA7 harbouring 28 SNPs spanning *CAST* and a 2.64-Mb region of BTA29 harbouring 93 SNPs spanning *CAPN1* were found to contain loci for which SNP ASEs ranked in the top 500 in the within-breed analyses. To allow haplotype-based analyses, we expanded the regions to 44 SNPs spanning 2.86 Mb for *CAST* and 100 SNPs spanning 3.12 Mb for *CAPN1* (Table S3). We first analysed each SNP individually by including allele effects (the difference between the two estimated allele effects is the ASE for the SNP) in β, in addition to the contemporary group effects, and then we included haplotype effects for windows of nine contiguous SNPs using phase information estimated by fastphase. The haplotype model was sequentially fit by sliding the nine SNP window through each region one SNP at a time, and the statistics computed for each window were assigned to the 5th SNP located at the centre of each window. In both cases, the analysis was performed using the previously estimated variance components (Table 1), and *F*-tests for SNP or haplotype effects were constructed from the difference between model sums of squares including and excluding the fitted SNP or haplotype effects, the difference in number of parameters between the fitted models and the estimated residual variance for the full model. Because the number of detected haplotypes varied throughout each region (Table S3), the window producing the largest model sum of squares does not necessarily result in the largest *F*-statistic or −log_10_*P*-value (because the numerator mean square can be significantly influenced when its degrees of freedom are small but vary between tests). To avoid this, we computed the percentage of phenotypic variation explained by each window through the region from the ratio of the window to phenotypic sums of squares, where the window sum of squares was estimated as the difference between model sum of squares including and excluding haplotype effects for the nine SNP window and the phenotypic sum of squares was estimated as the total sum of squares corrected for the mean and contemporary group sums of squares. This statistic identifies the SNP window that explains the largest amount of variation in WBSF regardless of the number of haplotypes that are fit.

## Results and discussion

We found large differences in the heritabilities of WBSF across the five breeds (Table 1) and were concerned that this might reflect differences in data quality or the correct assignment of phenotype to genotype because of the sample misidentification issue identified within the CMP. However, we also estimated heritabilities for eight additional carcass traits recorded in this project (data not shown) and found no evidence for systematically lower heritabilities within any of the breeds. We therefore conclude that the re-extraction of DNA from tissue samples taken from the carcass at slaughter effectively solved the misidentification problem. Thus, the variation in heritabilities probably reflects the relatively small sample size within each breed and the sampling of the bulls used to produce these animals. However, the effect of variation in heritability across breeds was to substantially influence the ‘genetic' sample size which we estimate as *N* × *h*, the number of phenotypes multiplied by the square root of the heritability, which is an estimate of the cumulative amount of additive genetic information in a sample of *N* unrelated individuals and was 468.3, 451.5, 471.7, 85.4 and 143.5 in Angus, Hereford, Charolais, Limousin and Simmental respectively.

In the across-breed analysis, the use of the genomic relationship matrix corrects for the stratification because of pedigree relatedness while accounting for the extent of background relatedness among the Angus and Continental breed groups because of the use of Angus dams to produce the crossbred Continental breed calves. In this analysis, the associations between the *CAST* and *CAPN1* loci with WBSF were the largest in the genome (Fig. 1), reflecting both the magnitude of effects of these genes and the increased SNP density within these regions, which improves the likelihood of finding SNP in strong LD with the causal mutations. The within-breed analyses identified *CAPN1* as the locus most strongly associated with WBSF genome-wide, although the highest ranked SNP ASE within this region for Limousin was only 30th (Table S2), presumably reflecting the very small sample size for this breed. On the other hand, the *CAST* associations were more variable among the breeds, being the most strongly associated with WBSF genome-wide in Hereford, ranking highly in Charolais and Limousin, but only 234th and 208th in Angus and Simmental respectively. These results are likely due to the fairly small sample sizes for the analysed breeds, but probably also may reflect the different SNP densities within the two regions and differences in allele frequencies at the SNPs and QTL across breeds. We accomplished a much higher SNP density in the region harbouring *CAPN1* than *CAST,* and this suggests that we had insufficient SNPs to find at least one that was in strong LD with the causal mutations within *CAST* in all breeds.

Across all 40 645 SNPs, the correlations between ASEs estimated within each of the breeds varied from −0.02 to 0.04, indicating that models developed to predict genomic breeding values within one breed will have very low accuracies in other breeds. This has previously been predicted using simulated data (de Roos *et al*. 2009; Toosi *et al*. 2010) but, despite the use of commercial Angus females to produce the Continental breed crossbred steers, it is a consequence of the genetic distance between the training and validation sets of animals. Habier *et al*. (2010) demonstrated that the number of generations that separate the training and validation data sets influences the accuracy of genomic breeding values estimated in the validation set, with lower accuracies occurring when this relationship is more distant. On the other hand, the correlations between the ASEs estimated in the across-breed analysis and those estimated in the within-breed analyses were 0.37, 0.66, 0.41, 0.31 and 0.42 for Angus, Hereford, Charolais, Limousin and Simmental respectively. This result supports the simulation results of Toosi *et al*. (2010), who showed that training in admixed populations results in genomic estimates of breeding value with accuracies almost equivalent to those achieved from training and validating within the same breed. Of course, the key benefits from the perspective of beef cattle breeding are that training population samples can dramatically be increased by pooling breeds and that the resulting genomic breeding values have industry-wide utility.

Hayes & Goddard (2001) have estimated that between 50 and 100 QTL underlie variation in quantitative traits within livestock populations. While under neutral theory, the common QTL mutations that are detectable by GWA analysis must predate the domestication of cattle (Kimura & Ohta 1973), the relatively small populations upon which breeds were founded may have led to the sampling of different subsets of QTL within different breeds. In fact, the extent to which breeds share common QTL is unknown (Pryce *et al*. 2010), but is of some importance to the development of prediction equations for molecular estimates of breeding value in admixed populations and the development and utilization of genotyping assays for the prediction of genetic merit within the beef industry. To identify QTL underlying variation in WBSF, we examined the genomic regions harbouring the 500 SNPs with the largest ASEs from the across-breed analysis for SNPs with ASEs ranked in the top 500 in the within-breed analyses for at least three of the breeds. We identified 79 genomic regions that putatively harbour QTL influencing WBSF (Table table by GWA analysis must predate the domestication of cattle (Kimura & Ohta). Of these, 42 were identified in three breeds, 29 in four breeds and eight in all five breeds. There was no difference between the breeds (*P*= 0.48) or between British and Continental breeds (*P*= 0.52) in the probability of QTL detection for all 79 QTL or for the 42 QTL identified in only three breeds (*P* = 0.35 and 0.82 respectively). Clearly sample size, assay SNP density, constraints on SNP ranks and the size of regions harbouring highly ranked SNP ASEs all impact the identification of putatively common QTL. Of the 113 instances when the within-breed estimated SNP ASEs ranked >500, the average rank was only 2551, suggesting that the majority of these regions harbour QTL that segregate in all breeds. Changing the minimum within-breed ASE rank criterion to <1000 resulted in 17 of these QTL being detected in all five breeds, 41 in four breeds and 21 in three breeds (Table 2). Thus, there appears to be little phylogenetic signal in these data, and if a QTL was detected in only three breeds, these breeds were as likely to be British and Continental as strictly Continental.

**Table 2:** Genomic regions identified as harbouring QTL that were detected in at least three breeds.

BTA	Start^1^	End^1^	SNP^2^	Location^3^	No. SNP^4^	Breeds	Angus^4^	Hereford^4^	Charolais^4^	Limousin^4^	Simmental^4^	All breeds^4^
1	27 034 490	29 073 969	*rs42409195*	28 111 487	30 (2)	C, L, S	7433	6333	37	189	19	335
1	155 725 361	156 105 357	*rs41600022*	155 725 361	8 (1)	H, L, S	2242	43	967	429	267	423
3	306 322	1 267 869	*ss86301348*	1 267 869	17 (1)	A, H, C	154	134	222	6584	3319	210
4	62 189 085	62 766 260	*rs43403458*	62 685 650	16 (2)	H, C, S	2695	244	292	1679	176	60
5	4 501 932	5 240 327	ss86306901^*^	5 012 505	15 (1)	A, H, S	90	422	8827	3688	453	458
5	21 876 606	23 103 768	*rs29014779*	21 876 606	19 (1)	C, L, S	846	3002	51	441	181	444
5	99 077 991	101 271 357	*rs41654473*	101 271 357	24 (1)	C, L, S	1105	1269	83	280	270	319
6	20 730 690	22 576 164	*rs42756258*	21 884 446	36 (2)	A, C, L, S	10	2467	191	304	78	190
6	102 116 041	104 245 701	*ss117968229*	103 281 884	44 (3)	A, L, S	214	625	1463	94	48	273
7	55 116 289	57 554 684	*rs29012174*	55 116 289	36 (1)	A, H, L, S	65	132	727	105	262	47
7	73 155 944	74 367 220	*ss86318554*	74 367 220	28 (1)	A, H, C, L	358	102	470	144	3570	288
7	77 854 696	83 621 039	*rs43527386*	80 731 488	89 (3)	H, C, L, S	1478	94	420	219	424	71
7	97 861 341	98 820 742	rs41255587^*^	98 579 574	19 (8)	A, H, C, L, S	237	1	14	37	308	10
7	106 927 241	108 205 624	*rs43531510*	106 927 241	24 (2)	H, C, S	8668	163	49	972	306	98
8	3 830 280	4 955 143	*rs41618019*	4 955 143	19 (1)	A, H, S	137	57	534	9307	189	296
8	43 890 714	46 946 557	*rs42312419*	43 890 714	48 (1)	H, C, L, S	3561	208	16	410	126	31
8	65 338 177	69 622 989	*ss117969253*	68 894 735	68 (4)	A, H, C, L, S	156	85	198	240	90	29
8	97 684 074	98 861 495	*ss86319219*	98 746 331	16 (1)	A, H, C, L	31	181	238	141	4390	184
8	112 287 843	113 301 368	*ss86338099*	112 824 694	28 (2)	A, C, L, S	76	1615	123	369	235	330
9	36 960 364	40 088 647	*rs41623216*	38 252 618	41 (2)	H, L, S	1224	410	1033	126	151	188
10	6 871 209	8 514 821	*ss86317616*	7 830 003	26 (1)	A, L, S	299	2813	3578	238	99	338
10	15 413 589	16 985 300	*ss86317957*	16 326 848	34 (1)	A, H, L, S	383	128	4565	486	451	113
10	29 278 086	31 692 125	*ss86305679*	29 278 086	29 (1)	A, H, L, S	162	184	896	449	293	161
10	38 799 891	40 135 969	*rs42412333*	39 278 374	18 (4)	A, H, S	222	120	4536	1974	336	211
10	96 842 358	98 541 920	*rs41590854*	97 410 796	26 (1)	A, H, L	239	415	777	113	764	262
10	102 286 251	103 234 411	*rs41596899*	102 308 122	25 (3)	H, C, L, S	3577	393	184	103	103	160
11	1 214 856	1 963 074	*ss86324631*	1 214 865	21 (1)	H, C, L, S	10476	235	469	173	107	124
11	31 734 782	33 348 373	*rs41606137*	32 224 661	26 (3)	A, L, S	288	1652	1054	336	168	241
12	35 454 037	36 764 448	*ss117970656*	35 581 416	20 (3)	H, C, S	3094	50	489	4969	211	149
12	50 715 278	52 618 243	*rs43699567*	52 573 538	40 (1)	A, H, C, L, S	416	288	385	352	27	498
13	3 723 531	5 128 166	*rs42862024*	4 308 889	22 (2)	A, H, S	107	381	3033	2879	341	305
13	29 072 163	33 201 457	*rs29011158*	31 826 409	64 (2)	A, H, C, L, S	315	242	31	36	4	151
13	66 080 035	69 702 161	*rs41631563*	66 080 035	72 (14)	A, H, C, S	471	8	61	787	142	97
13	73 369 210	73 746 516	*ss86338902*	73 746 516	9 (1)	A, H, S	344	127	594	2950	130	283
13	75 018 157	76 078 033	*ss86289318*	76 042 839	24 (2)	A, C, S	41	773	65	1767	110	43
13	80 848 032	81 665 695	*rs42630433*	81 029 787	21 (3)	A, H, C, L	386	48	69	41	5004	75
14	18 732 660	20 347 849	*rs41633333*	18 756 025	32 (5)	A, H, C	293	414	87	573	2160	76
14	47 926 524	48 572 837	*ss86299784*	48 184 967	13 (1)	C, L, S	2191	871	301	195	109	302
14	62 549 674	63 827 753	*ss86297726*	63 213 438	24 (1)	A, H, C, L	352	301	97	275	1445	166
15	31 599 942	33 310 389	*ss86291817*	32 861 621	32 (4)	A, H, L	311	31	1527	243	553	162
15	34 682 617	36 817 688	rs41757680^*^	35 661 186	40 (1)	A, H, C, L, S	99	21	53	32	468	354
15	48 688 111	50 222 093	*rs41582705*	48 936 679	10 (1)	C, L, S	4718	5799	172	162	124	119
15	62 309 986	63 517 557	*rs41621125*	63 253 454	20 (1)	H, C, L	9112	77	109	444	3538	74
15	64 876 840	66 717 899	*ss86314348*	64 876 840	15 (1)	H, C, L, S	1137	42	20	84	92	32
15	81 655 317	82 875 229	*ss86296417*	82 768 398	25 (1)	H, C, L	626	152	80	122	1842	178
16	11 797 915	13 358 683	*rs41623175*	12 130 589	23 (2)	A, H, C, L	18	18	4	272	1145	44
16	17 070 345	19 313 882	*ss86290236*	18 059 649	19 (1)	A, C, L, S	334	2017	256	381	96	353
16	22 147 468	23 830 920	*ss86329907*	22 406 467	17 (1)	A, H, C	401	88	354	1452	1920	216
16	25 000 153	28 384 914	*ss86291490*	27 629 566	39 (4)	H, C, L, S	1089	166	19	234	37	148
16	71 968 734	72 962 506	*rs41824081*	72 165 897	20 (2)	H, C, L	6937	265	467	55	2353	25
17	34 429 947	37 201424	*rs41626299*	34 429 947	25 (1)	H, C, L, S	1866	131	391	420	479	195
17	63 049 154	64 637 527	*ss86317522*	63 049 154	29 (1)	A, C, L, S	205	1220	347	454	391	278
17	73 315 120	74 393 620	*ss86339946*	73 315 120	27 (1)	A, C, S	166	551	361	5105	22	403
18	4 723 911	6 440 525	*ss86336538*	4 723 911	32 (1)	A, L, S	333	580	3125	151	251	83
18	55 028 139	55 621 823	*ss86310123*	55 590 144	10 (1)	A, H, S	363	418	5999	2353	28	489
20	15 870 897	17 710 059	*rs41933103*	17 175 071	35 (3)	H, C, L	1892	44	52	320	1009	36
20	64 002 006	66 587 451	ss86335963^*^	66 105 424	51 (2)	A, C, L, S	142	831	273	295	261	206
21	33 764 430	34 810 865	*rs29015146*	34 165 847	19 (1)	A, H, S	378	397	2032	924	322	434
21	40 955 783	43 096 903	*rs42503056*	40 955 783	30 (1)	A, H, S	116	350	4015	2961	113	85
21	59 665 710	61 121 046	*rs41585245*	61 121 046	22 (3)	A, C, L	458	703	211	205	1790	67
21	68 152 356	68 965 986	*ss86312849*	68 846 429	17 (4)	H, C, L	2122	108	209	83	1796	33
23	48 537 019	49 094 579	*rs41617911*	48 856 081	16 (1)	A, C, L	89	2461	332	448	2831	329
25	1 160 378	2 105 645	*ss117973580*	1 919 606	21 (2)	A, L, S	215	1633	2777	387	478	116
25	14 683 151	15 752 362	*ss86336453*	15 752 362	23 92)	A, C, L, S	96	1940	306	60	145	132
25	19 762 712	22 728 704	*rs41572366*	21 655 452	47 (2)	A, H, C, L, S	97	63	495	3	258	102
25	27 545 745	30 572 524	ss86283327^*^	29 485 851	48 (2)	A, H, C, L, S	57	499	99	102	68	49
26	12 580 311	14 127 433	*ss86273489*	13 293 856	27 (1)	A, H, S	27	107	4581	641	461	144
26	17 058 843	18 288 540	*ss86287439*	18 288 540	25 (2)	A, H, L, S	243	404	512	93	212	138
26	29 698 221	31 348 288	*rs41646897*	30 903 998	37 (1)	A, H, C, S	420	76	317	897	183	63
26	41 183 634	43 312 255	*ss86282954*	42 274 097	37 (2)	H, L, S	3947	23	701	256	445	388
27	3 343 936	6 388 642	*rs29024621*	3 909 806	24 (1)	A, H, L, S	275	80	2437	412	201	401
27	19 195 734	21 993 669	*rs42118878*	19 195 734	39 (4)	H, L, S	2323	323	538	192	42	35
27	34 978 041	36 054 950	*ss86310277*	35 372 600	21 (1)	A, H, C, S	304	425	149	1423	222	364
28	4 837 387	5 876 902	*rs41612729*	5 052 476	24 (3)	H, C, L, S	1466	317	438	117	233	280
28	31 700 004	34 066 383	*ss86337100*	33 570 352	33 (1)	A, H, L, S	39	138	3800	70	7	19
28	37 398 488	38 314 983	*rs29013966*	37 514 643	20 (1)	H, C, S	624	320	110	916	450	84
28	43 815 607	44 961 253	*ss86283362*	44 694 578	25 (1)	A, C, S	153	834	121	696	84	389
29	34 618 653	36 573 929	*rs29022154*	35 387 115	35 (2)	A, C, L, S	120	2258	276	35	87	129
29	44 042 363	44 087 629	rs42192103^*^	44 070 713	30 (18)	A, H, C, L, S	1	4	1	30	1	1

A, Angus; C, Charolais; H, Hereford; L, Limousin; S, Simmental; QTL, quantitative trait loci; SNP, single-nucleotide polymorphism.

UMD3.0 coordinates for the SNPs defining the boundaries of the SNP putatively harbouring the QTL.

Identity and UMD3.0 coordinate of the most strongly associated SNP within the interval as determined in the across-breed analysis. QTL previously reported in the Cow QTL Database (http://www.animalgenome.org/cgi-bin/QTLdb/BT/draw_traitmap?trait_ID=1030) are indicated with asterisks.

Number of SNPs within the interval. Number of SNPs within the region ranked in top 500 ASEs in the across-breed analysis in parentheses.

Lowest rank for *t*_*i*_ value within the interval.

We have previously found poor concordance between GWA and half-sib linkage analyses for large-effect QTL underlying growth traits, even when large numbers (>50) of families with family sizes ranging from 20 to 224 half-sibs are analysed (data not shown). Assuming that GWA analysis detects common variants, we would expect a significant number of sires to be both heterozygous and detected to be segregating for a large-effect QTL; however, this largely depends on the underlying genetic architecture of the trait. Reed *et al*. (2008) found that growth was affected in 34% of viable mouse knockouts, suggesting that natural variation in thousands of genes underlies variation in growth. As a consequence of this complex genetic architecture, there may be a large number of QTL on each chromosome, and the allelic combinations present at these QTL in the sire will impact on whether any one QTL is detected in linkage analyses. Thus, common variants detected in GWA analysis may not be detected in segregation analysis, and rare variants detected in segregation analysis may not be detected in GWA analysis. Nevertheless, we found six of the 12 previously reported meat tenderness QTL, including *CAST* and *CAPN1,* to coincide with the QTL identified in this study (Table 2) (Cattle QTL database, http://www.animalgenome.org/cgi-bin/QTLdb/BT/draw_traitmap?trait_ID=1030, accessed June 27, 2011). Notwithstanding the poor resolution of QTL location mapped by linkage analysis, we also found support for all of the other previously identified QTL. For example, in the across-breed analysis, QTL were identified with ASE ranks <500 at 3 151 989 bp and at 6 831 955–7 086 105 bp (300 kb from *MSTN*) on BTA2. The first was supported by ASE ranks <500 for Angus and Charolais, but an ASE rank of 565 in Limousin. The second was supported by an ASE rank <500 in Charolais and ASE ranks <1000 in Angus, Limousin and Simmental. Thus, despite their proximity, these QTL are likely distinct, and the concordance between our and previously published results suggests that the genetic architecture of meat tenderness is substantially less complex than for growth.

We examined the genomic regions harbouring the 37 QTL that were detected in at least four of the breeds for potential candidate genes underlying meat tenderness. Very little is known about the genetic regulation of meat tenderness, and few candidate genes are suggested for these QTL. While *CAST* and *CAPN1* have consistently been identified and analysed as candidate genes for the BTA7 97 861 341–98 820 742-bp and BTA29 44 042 363–44 087 629-bp QTL, respectively, no causal variants have been identified in either gene. *CAPN1* encodes the protease μ-calpain, which has been implicated in the proteolysis of muscle proteins during meat ageing (Smith *et al*. 2000), and *CAST* encodes calpastatin, which is an inhibitor of μ-calpain (Goll *et al*. 2003). Myogenic determination factor 1 is a transcription factor encoded by *MYOD1* and is expressed in skeletal muscle during myogenesis and regeneration. Variation in *MYOD1* has been suggested to affect its ability to influence the expression of muscle structural components (Rexroad *et al*. 2001), making it a candidate for the QTL at 34 682 617–36 817 688 bp on BTA15. Calpain-2 (m/II) large subunit (m-calpain) is a calcium-activated neutral protease encoded by *CAPN2* on BTA16 (25 000 153–28 384 914 bp). M-calpain activity has been associated with both meat tenderness and palatability measurements (Riley *et al*. 2003). Fibroblast growth factor 2 (*FGF2*) is an upstream regulator of heat shock protein B1 (*HSPB1*), which has been found to be negatively related to WBSF (Kim *et al*. 2011), making it a candidate for the 34 429 947–37 201 424-bp QTL on BTA17. *GSN* encodes gelsolin, a calcium-regulated protein that functions in both the assembly and disassembly of actin filaments, which are a component of the contractile apparatus in muscle cells and may underlie the BTA8 112 287 843–113 301 368-bp QTL. Finally, *CALM1* encodes calmodulin, a calcium-binding protein, which interacts with titin and mediates smooth muscle contraction, making it a candidate for the BTA10 102 286 251–103 234 411-bp QTL.

**Figure 2:**
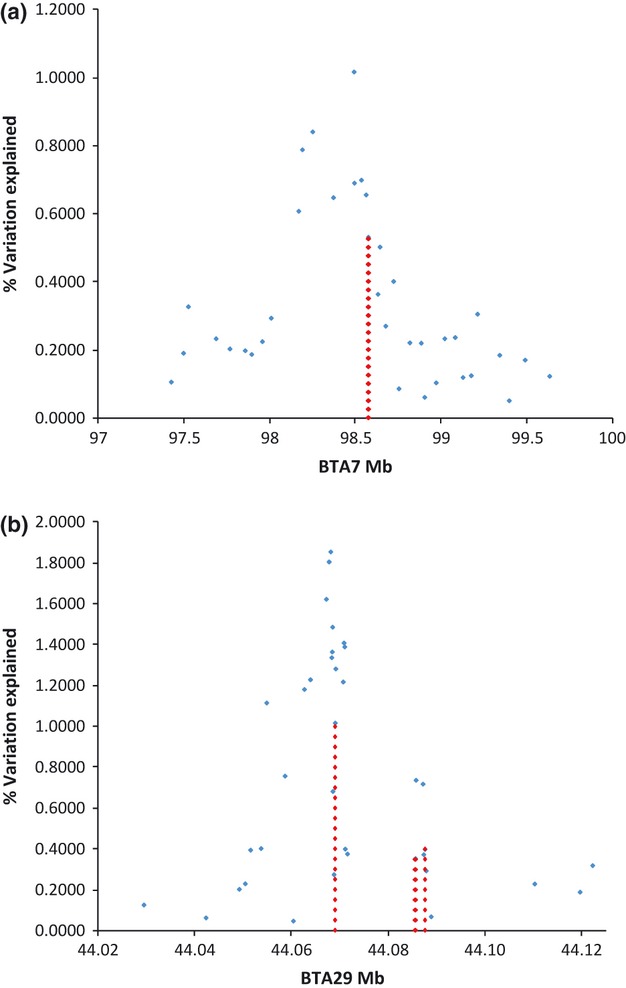
Proportion of phenotypic variation in the across-breed analysis explained by haplotypes constructed from nine consecutive single-nucleotide polymorphism (SNPs) in the region of (a) BTA7 harbouring *CAST* and (b) BTA29 harbouring *CAPN1*. Locations and amount of variation explained by the commercialized tenderness SNPs are indicated by red dotted lines.

While the commercially tested *CAST* SNP *rs41255587* was the most strongly associated with WBSF in the across-breed analysis (−log_10_*P* = 8.95), it was only the most strongly associated *CAST* SNP within Hereford and Charolais, with stronger associations being detected for SNPs in the 5′ upstream region in Angus, Limousin and Simmental (Table S3). In fact, the haplotype analysis moves the location of the most significantly associated SNP window 83.7 kb upstream of *rs41255587* to be centred on *rs43529872* (−log_10_*P* = 8.78), and this *CAST* window was found to explain the greatest amount of phenotypic variation in WBSF in the across-breed (1.02%; Table 3), Angus and Hereford analyses. The sign and magnitude of the ASE was consistent for *rs41255587* in all breeds except Limousin, and the haplotype analysis explained considerably more variation in WBSF than the single SNP analysis, indicating that either the causal variant is not among the tested polymorphisms or that there is more than one causal variant. Furthermore, the haplotype analyses move the most likely location of the causal mutation 5′ of the commercially tested *CAST* SNP *rs41255587,* probably in the 678-kb region from 97 861 341–98 538 952 bp (Fig. 2). Clearly, additional fine-mapping is required to identify the number of mutations influencing WBSF that lie in the vicinity of *CAST* and their most likely locations.

**Table 3:** Percentages of phenotypic variation in WBSF explained by the commercialized SNPs, the most strongly associated SNPs and haplotypes within the most strongly associated nine SNP window within *CAST* and *CAPN1*.

Locus	All breeds	Angus	Hereford	Charolais	Limousin	Simmental
*CAST* (BTA7)						
*rs41255587*^1^ 98 579 574	0.66	0.53	1.47	1.14	0.70	0.02
SNP^2^	0.66 98 579 574	0.54 98 498 047	1.47 98 579 574	1.14 98 579 574	2.28 97 861 341	1.13 98 013 150
Window-P^3^	1.02 98 495 888	1.36 98 495 888	1.88 98 566 391	2.10 98 538 952	3.88 97 501 859	2.77 97 861 341
Window-V_P_^4^	1.02 98 495 888	1.36 98 495 888	1.92 98 495 888	2.10 98 538 952	4.02 98 375 640	2.77 97 861 341
*CAPN1* (BTA29)						
*rs17812000*^1^ 44 069 063	1.14	2.36	0.96	1.38	0.00	3.75
*rs17871051*^1^ 44 085 642	0.39	1.54	0.16	0.39	0.57	1.66
*rs17872050*^1^ 44 097 629	0.53	0.89	0.08	1.21	2.88	1.65
SNP^2^	1.16 44 070 713	2.36 44 069 063	1.62 44 067 796	1.57 44 070 713	2.88 44 087 629	4.65 44 042 363
Window-P^3^	1.80 44 067 796	3.18 44 068 519	2.59 44 062 694	2.76 44 070 881	2.99 44 087 356	5.05 44 067 234
Window-V_P_^4^	1.85 44 068 143	3.19 44 068 445	2.59 44 062 694	2.76 44 070 881	3.52 44 070 881	5.35 44 068 143

*CAST*, *calpastatin*; *CAPN1*, *calpain 1, (mu/I) large subunit*; SNP, single-nucleotide polymorphism; WBSF, Warner–Bratzler shear force.

Commercialized SNP and its chromosomal coordinate.

Most strongly associated SNP and its chromosomal coordinate.

Most strongly associated nine SNP window centred on SNP with shown chromosomal coordinate.

Nine SNP window explaining the greatest amount of phenotypic variation in WBSF.

Among the SNP located within *CAPN1*, *rs17812000* (c.316G>A) was most strongly associated with WBSF in Angus (−log_10_*P* = 9.70) and *rs17872050* was the most strongly associated with WBSF in Limousin (−log_10_*P* = 3.23). However, *rs42192103* was found to be slightly more strongly associated with WBSF than *rs17812000* in the across-breed analysis (−log_10_*P* = 15.25 vs. 15.01), with an average ASE across breeds of 0.23 kg (Table S3). The amount of phenotypic variation explained in the haplotype-based analyses again indicates that none of the tested SNPs are causal for effects on WBSF and that the strongest signal for association with WBSF was in the 8187-bp region from 44 062 694 to 44 070 881 in all five breeds (Table S3). The size of this region is sufficiently small to speculate that there is probably only a single mutation in *CAPN1* affecting WBSF in all *Bos t. taurus* cattle breeds, and the across-breed haplotype analysis shown in Table S3 and Fig.).

## Conclusions

We conclusively demonstrate that none of the SNPs currently commercialized as diagnostics for genetic merit are causal for their effects on WBSF (Casas *et al*. 2003, 2006; Van Eenennaam *et al*. 2007; Gill *et al*. 2009). In fact, the complex patterns of LD in the vicinity of these genes among the different breeds (Figs S2 and S3) and the weaker associations in Limousin and Simmental (Fig. S1) result in different SNPs being most strongly associated with WBSF among the breeds (Table S3). However, by using haplotype-based analysis methods to dissect the variation within these genes, we localized the causal variants to be 5′ to the commercially tested SNPs. In the case of *CAPN1*, the higher SNP density achieved and the use of across-breed analysis, which erodes the patterns of LD within breeds, resolved the likely location of the causal variant to a region of only 4581 bp.

We found evidence for a large number of QTL underlying variation in WBSF, and the majority of the previously published QTL were validated in this analysis. We found reasonably strong evidence that most QTL were segregating in all five breeds; however, the small genetic sample sizes for Limousin and Simmental make this comparison problematic, and it remains an unanswered question as to the extent to which breeds may share private alleles at QTL. This has previously been found in Belgian Blue, Marchigiana and Piedmontese cattle, where breed-specific polymorphisms in *MSTN* produce the double muscled phenotype (Grobet *et al*. 1997; Kambadur *et al*. 1997; McPherron & Lee 1997; Marchitelli *et al*. 2003). This issue is of importance to the development of prediction equations for molecular breeding values in across-breed analyses, because the ASEs estimated for QTL regions will be averaged across breeds that segregate and those that do not segregate for certain QTL, which will limit the accuracy of molecular estimates of breeding value. Despite this, we found moderate correlations between GBLUP predictions of ASEs computed in the across- and within-breed analyses, suggesting that the BovineSNP50 assay has sufficient resolution for the development of prediction equations for genomic selection in beef cattle despite their considerably larger effective population size relative to dairy cattle (The Bovine HapMap Consortium 2009), and also that WBSF QTL are commonly shared among breeds.

Despite the apparent reduced complexity of a trait such as meat tenderness relative to growth, there appear to be a large number of QTL underlying variation in WBSF, and the identification of all of the mutations that underlie these QTL might appear to be intractable. However, recent developments in high-density SNP genotyping, high-throughput sequencing and genotype imputation suggest new strategies for the rapid simultaneous identification of variants underlying quantitative traits genome-wide. We accomplished an average SNP spacing of 1139 bp for the 23 SNPs analysed within *CAPN1,* and this is only slightly smaller than could be accomplished genome-wide by jointly genotyping with the newly available Illumina BovineHD and Affymetrix BOS 1 assays (∼1.3 million SNP, data not shown). Furthermore, the design of these assays was facilitated by a community effort that produced more than 128.4X of genome sequence coverage on more than 80 animals, and SNP data from this work are now available in dbSNP. This project discovered 48.6 million high-quality SNPs, which must include many of the causal variants underlying quantitative variation in cattle, and it may be possible to impute genotypes at the resolution of the genome sequence (Daetwyler *et al*. 2011) in populations that have been genotyped with both assays. Such a strategy could rapidly allow the identification of a large number of causal variants if the association analysis was performed in mixed breed populations.

## Supporting information

Figure S1 Manhattan plots of normalized single-nucleotide polymorphism allele substitution effects for each breed.

Figure S2 Linkage disequilibrium (LD) plots (r^2^) created in haploview v4.1 for 44 single-nucleotide polymorphisms spanning 2.86 Mb centred on *calpastatin* on BTA7.

Figure S3 Linkage disequilibrium (LD) plots (r^2^) created in haploview v4.1 for the 100 single-nucleotide polymorphisms spanning 3.12 Mb centred on *CAPN1* on BTA29.

Table S1 Characteristics of single-nucleotide polymorphisms located near *calpastatin* and *calpain 1, (mu/I) large subunit* that were designed into the Illumina GoldenGate assay and genotyped in 3240 CMP animals.

Table S2 Standardized single-nucleotide polymorphism allele substitution effects, within-breed t-like statistic ranks, heterozygosity, allele frequency and sliding window rank information.

Table S3 Patterns of single-nucleotide polymorphism association with Warner–Bratzler shear force for *calpastatin* and *calpain 1, (mu/I) large subunit* loci.

As a service to our authors and readers, this journal provides supporting information supplied by the authors. Such materials are peer-reviewed and may be re-organized for online delivery, but are not copy-edited or typeset. Technical support issues arising from supporting information (other than missing files) should be addressed to the authors.

## References

[b1] Alexander LJ, MacNeil MD, Geary TW, Snelling WM, Rule DC, Scanga JA (2007). Quantitative trait loci with additive effects on palatability and
fatty acid composition of meat in a Wagyu–Limousin F2
population. Animal Genetics.

[b3] Boleman SJ, Boleman SL, Miller RK (1997). Consumer evaluation of beef of known categories of
tenderness. Journal of Animal Science.

[b4] Casas E, Keele JW, Shackelford SD, Koohmaraie M, Sonstegard TS, Smith TP, Kappes SM, Stone RT (1998). Association of the muscle hypertrophy locus with carcass traits
in beef cattle. Journal of Animal Science.

[b5] Casas E, Shackelford SD, Keele JW, Stone RT, Kappes SM, Koohmaraie M (2000). Quantitative trait loci affecting growth and carcass composition
of cattle segregating alternate forms of
*myostatin*. Journal of Animal Science.

[b6] Casas E, Stone RT, Keele JW, Shackelford SD, Kappes SM, Koohmaraie M (2001). A comprehensive search for quantitative trait loci affecting
growth and carcass composition of cattle segregating alternative forms of
the *myostatin* gene. Journal of Animal Science.

[b7] Casas E, Shackelford SD, Keele JW, Koohmaraie M, Smith TP, Stone RT (2003). Detection of quantitative trait loci for growth and carcass
composition in cattle. Journal of Animal Science.

[b8] Casas E, White SN, Wheeler TL, Shackelford SD, Koohmaraie M, Riley DG, Chase CC, Johnson DD, Smith TP (2006). Effects of *calpastatin* and micro-calpain markers
in beef cattle on tenderness traits. Journal of Animal Science.

[b9] Daetwyler HD, Wiggans GR, Hayes BJ, Woolliams JA, Goddard ME (2011). Imputation of missing genotypes from sparse to high density using
long-range phasing. Genetics.

[b10] Davis GP, Moore SS, Drinkwater RD, Shorthose WR, Loxton ID, Barendse W, Hetzel DJ (2008). QTL for meat tenderness in the M. longissimus lumborum of
cattle. Animal Genetics.

[b11] Gill JL, Bishop SC, McCorquodale C, Williams JL, Wiener P (2009). Association of selected SNP with carcass and taste panel assessed
meat quality traits in a commercial population of Aberdeen Angus-sired beef
cattle. Genetics Selection Evolution.

[b12] Gill JL, Bishop SC, McCorquodale C, Williams JL, Wiener P (2010). Associations between single nucleotide polymorphisms in multiple
candidate genes and carcass and meat quality traits in a commercial
Angus-cross population. Meat Science.

[b13] Goll DE, Thompson VF, Li H, Wei W, Cong J (2003). The calpain system. Physiological Reviews.

[b14] Grobet L, Martin LJ, Poncelet D (1997). A deletion in the bovine *myostatin* gene causes
the double-muscled phenotype in cattle. Nature Genetics.

[b15] Gutierrez-Gil B, Wiener P, Nute GR, Burton D, Gill JL, Wood JD, Williams JL (2008). Detection of quantitative trait loci for meat quality traits in
cattle. Animal Genetics.

[b16] Habier D, Tetens J, Seefried F, Lichtner P, Thaller G (2010). The impact of genetic relationship information on genomic
breeding values in German Holstein cattle. Genetic Selection Evolution.

[b17] Hayes B, Goddard ME (2001). The distribution of the effects of genes affecting quantitative
traits in livestock. Genetics Selection Evolution.

[b18] Huffman KL, Miller MF, Hoover LC, Wu CK, Brittin HC, Ramsey CB (1996). Effect of beef tenderness on consumer satisfaction with steaks
consumed in the home and restaurant. Journal of Animal Science.

[b19] Johnston DJ, Graser HU (2010). Estimated gene frequencies of GeneSTAR markers and their size of
effects on meat tenderness, marbling, and feed efficiency in temperate and
tropical beef cattle breeds across a range of production
systems. Journal of Animal Science.

[b20] Kambadur R, Sharma M, Smith TP, Bass JJ (1997). Mutations in *myostatin* (GDF8) in double-muscled
Belgian Blue and Piedmontese cattle. Genome Research.

[b21] Keele JW, Shackelford SD, Kappes SM, Koohmaraie M, Stone RT (1999). A region on bovine chromosome 15 influences beef longissimus
tenderness in steers. Journal of Animal Science.

[b22] Kim N-K, Lim D, Lee S-H, Cho Y-M, Park E-W, Lee C-S, Shin B-S, Kim T-H, Yoon D (2011). Heat shock protein B1 and its regulator genes are negatively
correlated with intramuscular fat content in the longissimus thoracis muscle
of Hanwoo (Korean cattle) steers. Journal of Agricultural and Food Chemistry.

[b23] Kimura M, Ohta T (1973). The age of a neutral mutant persisting in a finite
population. Genetics.

[b24] Lorenzen CL, Hale DS, Griffin DB, Savell JW, Belk KE, Frederick TL, Miller MF, Montgomery TH, Smith GC (1993). National Beef Quality Audit: survey of producer-related defects
and carcass quality and quantity attributes. Journal of Animal Science.

[b25] Marchitelli C, Savarese MC, Crisa A, Nardone A, Marsan PA, Valentini A (2003). Double muscling in Marchigiana beef breed is caused by a stop
codon in the third exon of myostatin gene. Mammalian Genome.

[b26] Matukumalli LK, Lawley CT, Schnabel RD (2009). Development and characterization of a high density SNP genotyping
assay for cattle. PLoS ONE.

[b27] McKay SD, Schnabel RD, Murdoch BM (2007). Whole genome linkage disequilibrium maps in
cattle. BMC Genetics.

[b28] McKenna DR, Roebert DL, Bates PK (2002). National Beef Quality Audit-2000: survey of targeted cattle and
carcass characteristics related to quality, quantity, and value of fed
steers and heifers. Journal of Animal Science.

[b29] McPherron AC, Lee SJ (1997). Double muscling in cattle due to mutations in the
*myostatin* gene. Proceedings of the National Academy of Sciences of the United States of
America.

[b30] Meuwissen TH, Hayes BJ, Goddard ME (2001). Prediction of total genetic value using genome-wide dense marker
maps. Genetics.

[b31] Miller MF, Huffman KL, Gilbert SY, Hamman LL, Ramsey CB (1995). Retail consumer acceptance of beef tenderized with calcium
chloride. Journal of Animal Science.

[b32] Miller MF, Carr MA, Ramsey CB, Crockett KL, Hoover LC (2001). Consumer thresholds for establishing the value of beef
tenderness. Journal of Animal Science.

[b33] Minick JA, Dikeman ME, Pollak EJ, Wilson DE (2004). Heritability and correlation estimates of Warner-Bratzler shear
force and carcass traits from Angus-, Charolais-, Hereford, and
Simmental-sired cattle. Canadian Journal of Animal Science.

[b34] Mintert J, Lusk JL, Schroeder TC, Fox JA, Koohmaraie M (2000).

[b35] Morris CA, Cullen NG, Hickey SM, Dobbie PM, Veenvliet BA, Manley TR, Pitchford WS, Kruk ZA, Bottema CD, Wilson T (2006). Genotypic effects of calpain 1 and *calpastatin*
on the tenderness of cooked *M. longissimus* dorsi steaks
from Jersey × Limousin, Angus and Hereford-cross
cattle. Animal Genetics.

[b36] Moser DW, Thallman RM, Pollak EJ, Dikeman ME, Gill CA, Koontz SR, Holm TR, Dressler EW (2004). Meeting consumer demands through genetic selection: the NCBA
Carcass Merit Project. Proceedings of the Beef Improvement Federation.

[b37] Page BT, Casas E, Heaton MP (2002). Evaluation of single-nucleotide polymorphisms in
*CAPN1* for association with meat tenderness in
cattle. Journal of Animal Science.

[b38] Page BT, Casas E, Quaas RL (2004). Association of markers in the bovine *CAPN1* gene
with meat tenderness in large crossbred populations that sample influential
industry sires. Journal of Animal Science.

[b39] Platter WJ, Tatum JD, Belk KE, Koontz SR, Chapman PL, Smith GC (2005). Effects of marbling and shear force on consumers'
willingness to pay for beef strip loin steaks. Journal of Animal Science.

[b40] Pryce JE, Bolormaa S, Chamberlain AJ, Bowman PJ, Savin K, Goddard ME, Hayes BJ (2010). A validated genome-wide association study in 2 dairy cattle
breeds for milk production and fertility traits using variable length
haplotypes. Journal of Dairy Science.

[b41] Reed DR, Lawler MP, Tordoff MG (2008). Reduced body weight is a common effect of gene knockout in
mice. BMC Genetics.

[b42] Rexroad CE, Bennett GL, Stone RT, Keele JW, Fahrenkrug SC, Freking BA, Kappes SM, Smith TP (2001). Comparative mapping of BTA15 and HSA11 including a region
containing a QTL for meat tenderness. Mammalian Genome.

[b43] Riley DG, Chase CC, Pringle TD, West RL, Johnson DD, Olson TA, Hammond AC, Coleman SW (2003). Effect of sire on mu- and m-calpain activity and rate of
tenderization as indicated by myofibril fragmentation indices of steaks from
Brahman cattle. Journal of Animal Science.

[b44] Roeber DL, Cannell RC, Belk KE, Tatum JD, Smith GC (2000). Effects of a unique application of electrical stimulation on
tenderness, color, and quality attributes of the beef longissimus
muscle. Journal of Animal Science.

[b45] de Roos APW, Hayes BJ, Goddard ME (2009). Reliability of genomic predictions across multiple
populations. Genetics.

[b46] Sambrook J, Fritsch EF, Maniatis T (1989). Molecular Cloning: A Laboratory Manual.

[b47] Scheet P, Stephens M (2006). A fast and flexible statistical model for large-scale population
genotype data: applications to inferring missing genotypes and haplotypic
phase. American Journal of Human Genetics.

[b48] Shook JN, Vanoverbeke DL, Scanga JA, Belk KE, Savell JW, Lawrence TE, Morgan JB, Griffin DB, Hale DS, Smith GC (2008). The national Beef Quality Audit-2005, Phase 1: views of
producers, packers, and merchandisers on current quality characteristics of
the beef industry. The Professional Animal Scientist.

[b49] Smith TP, Casas E, Rexroad CE, Kappes SM, Keele JW (2000). Bovine *CAPN1* maps to a region of BTA29
containing a quantitative trait locus for meat tenderness. Journal of Animal Science.

[b50] Smith GC, Savell JW, Morgan JB, Lawrence TE (2006). Report of the June–September 2005 national beef quality
audit: A new benchmark for the U.S. beef industry. Proceedings of the Beef Improvement Federation.

[b51] Stone RT, Casas E, Smith TP, Keele JW, Harhay G, Bennett GL, Koohmaraie M, Wheeler TL, Shackelford SD, Snelling WM (2005). Identification of genetic markers for fat deposition and meat
tenderness on bovine chromosome 5: development of a low-density single
nucleotide polymorphism map. Journal of Animal Science.

[b52] Thallman RM, Moser DW, Dressler EW, Totir RL, Fernando RL, Kachman SD, Rumph JM, Dikeman ME, Pollak JE (2003). Carcass merit project: DNA marker validation Proc. Beef Improv.
Fed. 8th Genet. Prediction Workshop.

[b53] The Bovine HapMap Consortium (2009). Genome wide survey of SNP variation uncovers the genetic
structure of cattle breeds. Science.

[b54] Toosi A, Fernando RL, Dekkers JCM (2010). Genomic selection in admixed and crossbred
populations. Journal of Animal Science.

[b55] Van Eenennaam AL, Li J, Thallman RM, Quaas RL, Dikeman ME, Gill CA, Franke DE, Thomas MG (2007). Validation of commercial DNA tests for quantitative beef quality
traits. Journal of Animal Science.

[b56] VanRaden PM (2008). Efficient methods to compute genomic predictions. Journal of Dairy Science.

[b57] Weston AR, Rogers RW, Althen TG (2002). Review: The role of collagen in meat tenderness. The Professional Animal Scientist.

[b58] Wheeler TL, Shackelford SD, Koohmaraie M (1998). Cooking and palatability traits of beef longissimus steaks cooked
with a belt grill or an open hearth electric broiler. Journal of Animal Science.

[b59] White SN, Casas E, Wheeler TL, Shackelford SD, Koohmaraie M, Riley DG, Chase CC, Johnson DD, Keele JW, Smith TP (2005). A new single nucleotide polymorphism in *CAPN1*
extends the current tenderness marker test to include cattle of *Bos
indicus Bos taurus*, and crossbred descent. Journal of Animal Science.

[b61] Zimin AV, Delcher AL, Florea L (2009). A whole-genome assembly of the domestic cow, *Bos
taurus*. Genome Biology.

