# Supplementary figures and images for: Genome-wide association analysis for quantitative trait loci influencing Warner–Bratzler shear force in five taurine cattle breeds
Source: Anim Genet. 2012 Feb 27;43(6):662–73. doi: 10.1111/j.1365-2052.2012.02323.x (PMC3506923; doi:10.1111/j.1365-2052.2012.02323.x)

**Figure S1.** Manhattan plots of normalized SNP allele substitution effects for each breed.

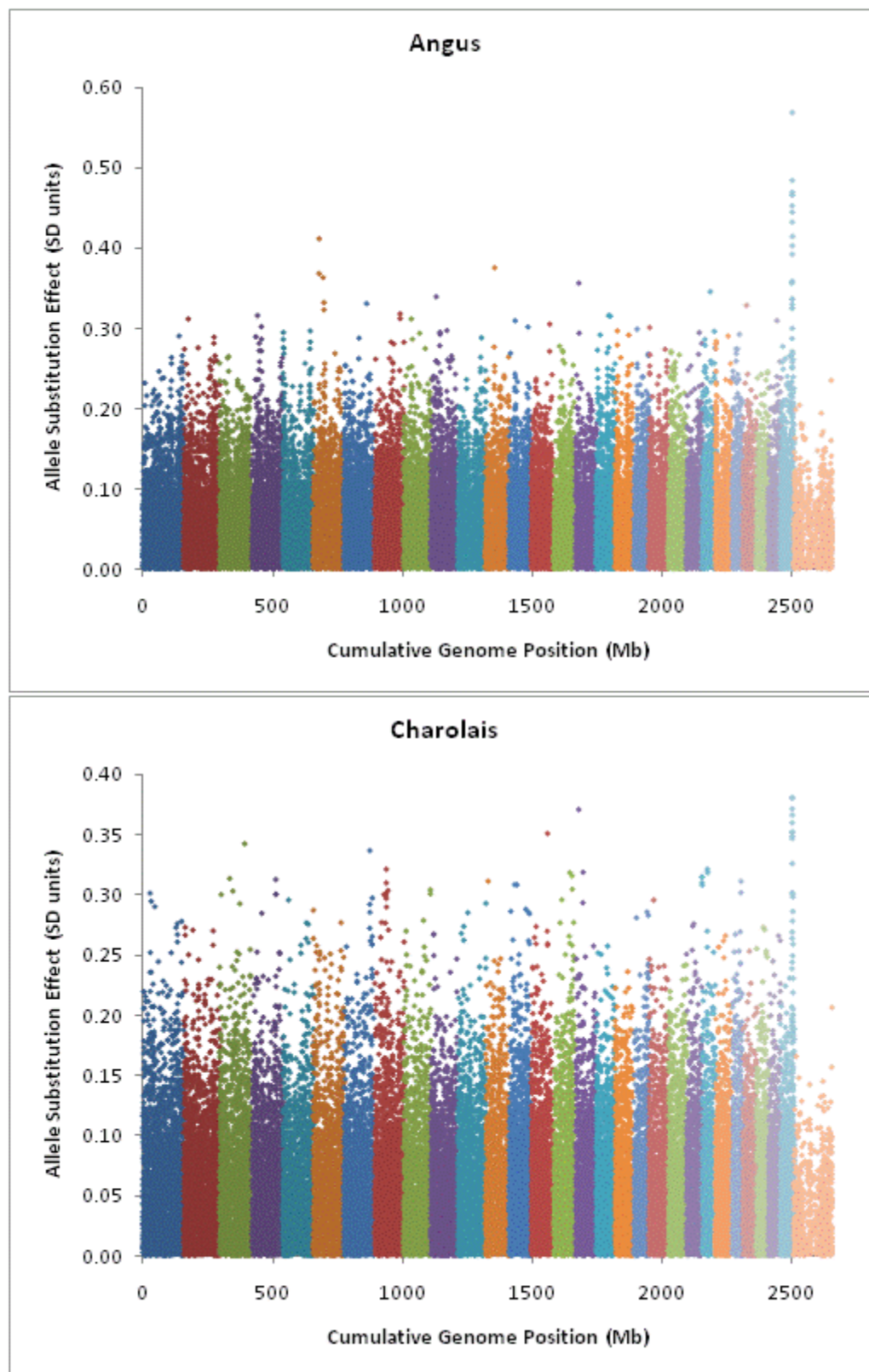

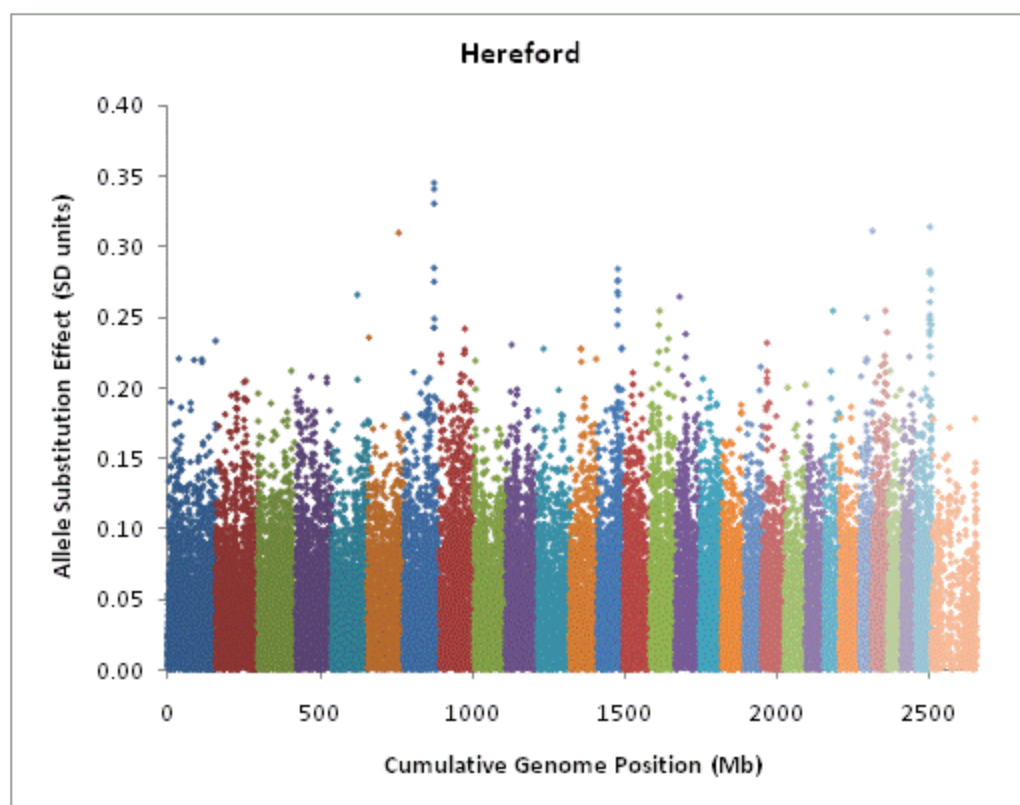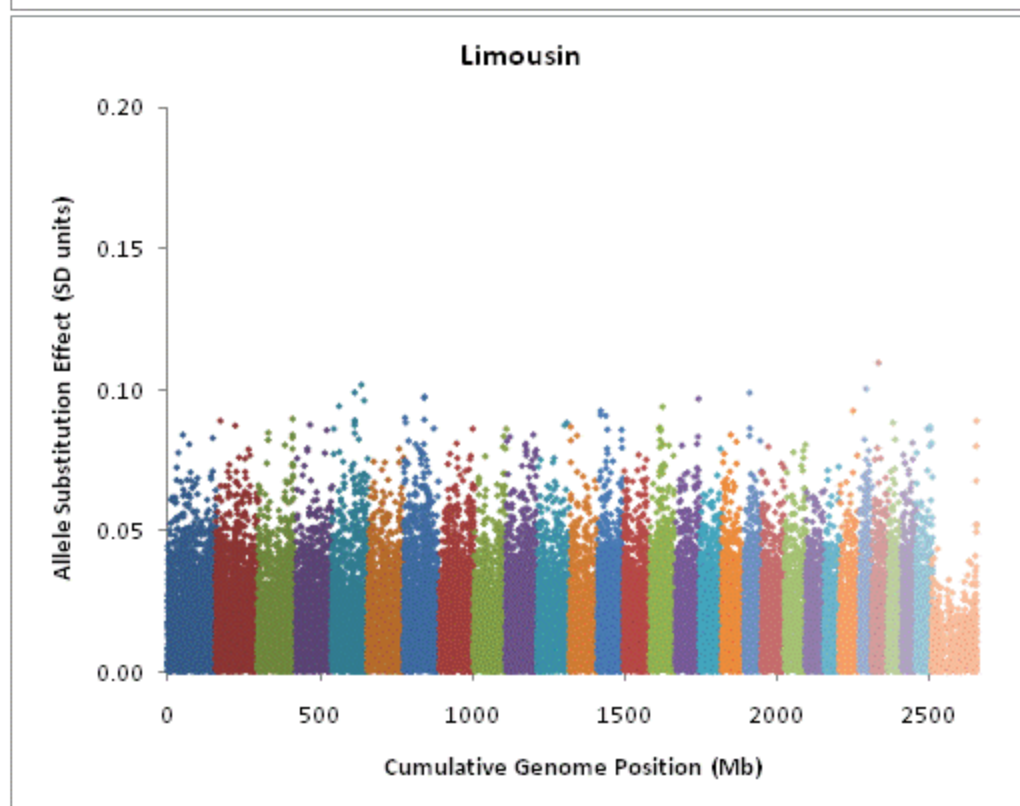

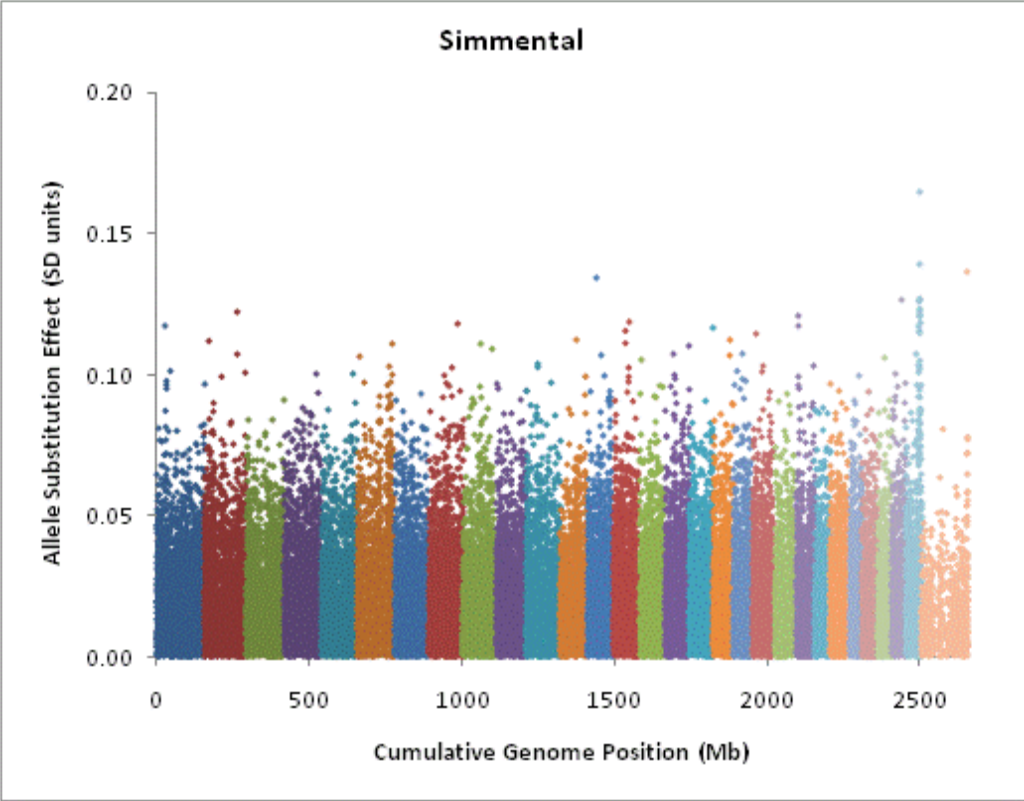

Supplement: Figure S1 — Manhattan plots of normalized single-nucleotide polymorphism allele substitution effects for each breed. [file age0043-0662-sd1.pdf]
