# Supplementary material for: Genome-wide association analysis for quantitative trait loci influencing Warner–Bratzler shear force in five taurine cattle breeds
Source: Anim Genet. 2012 Feb 27;43(6):662–73. doi: 10.1111/j.1365-2052.2012.02323.x (PMC3506923; doi:10.1111/j.1365-2052.2012.02323.x)
Supplement: Figure S2 — Linkage disequilibrium (LD) plots (r2) created in haploview v4.1 for 44 single-nucleotide polymorphisms spanning 2.86 Mb centred on calpastatin on BTA7. [file age0043-0662-sd2.pdf]

**Figure S2** Linkage disequilibrium (LD) plots ($r^2$) created in Haploview v4.1 (Barrett *et al*. 2005) for 44 SNP spanning 2.86 Mb centered on *CAST* on BTA7. Darker shading indicates regions of higher LD. The commercially tested SNP, *rs41255587*, (#22) is highlighted in green.

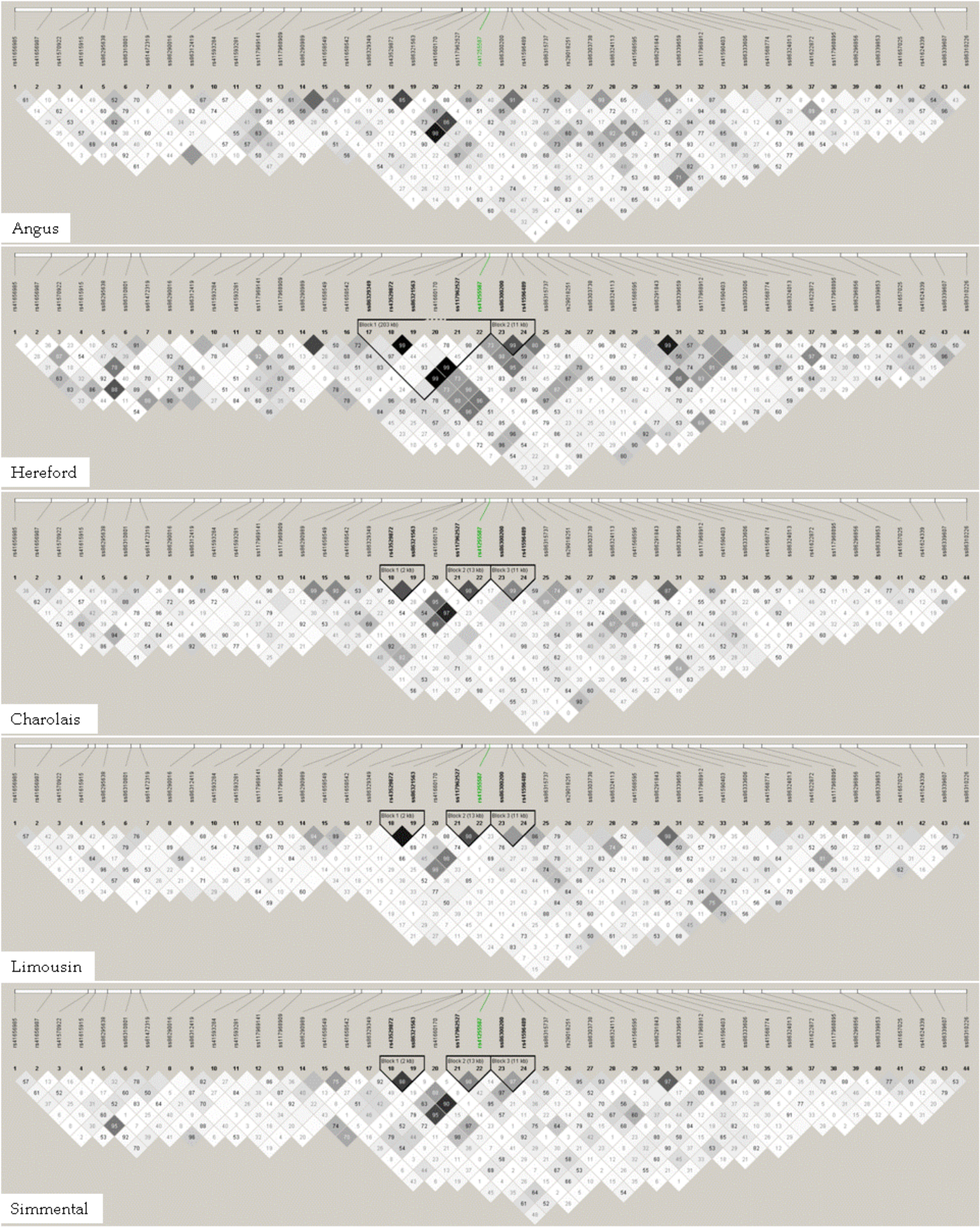

**References**

Barrett J.C., Fry B., Maller J. & Daly M.J. (2005) Haploview: analysis and visualization of LD and haplotype maps. *Bioinformatics* **21,** 263-5.
