# Supplementary material for: Genome-wide association analysis for quantitative trait loci influencing Warner–Bratzler shear force in five taurine cattle breeds
Source: Anim Genet. 2012 Feb 27;43(6):662–73. doi: 10.1111/j.1365-2052.2012.02323.x (PMC3506923; doi:10.1111/j.1365-2052.2012.02323.x)
Supplement: Figure S3 — Linkage disequilibrium (LD) plots (r2) created in haploview v4.1 for the 100 single-nucleotide polymorphisms spanning 3.12 Mb centred on CAPN1 on BTA29. [file age0043-0662-sd3.pdf]

Figure S3 Linkage disequilibrium (LD) plots (r^2) created in Haploview v4.1 (Barrett *et al*. 2005) for the 100 SNP spanning 3.12 Mb centered on *CAPN1* on BTA29. Darker shading indicates regions of higher LD. The commercially tested SNP, *rs17812000* (#51), *rs17871051* (#58), and *rs17872050* (#62) are highlighted in green.

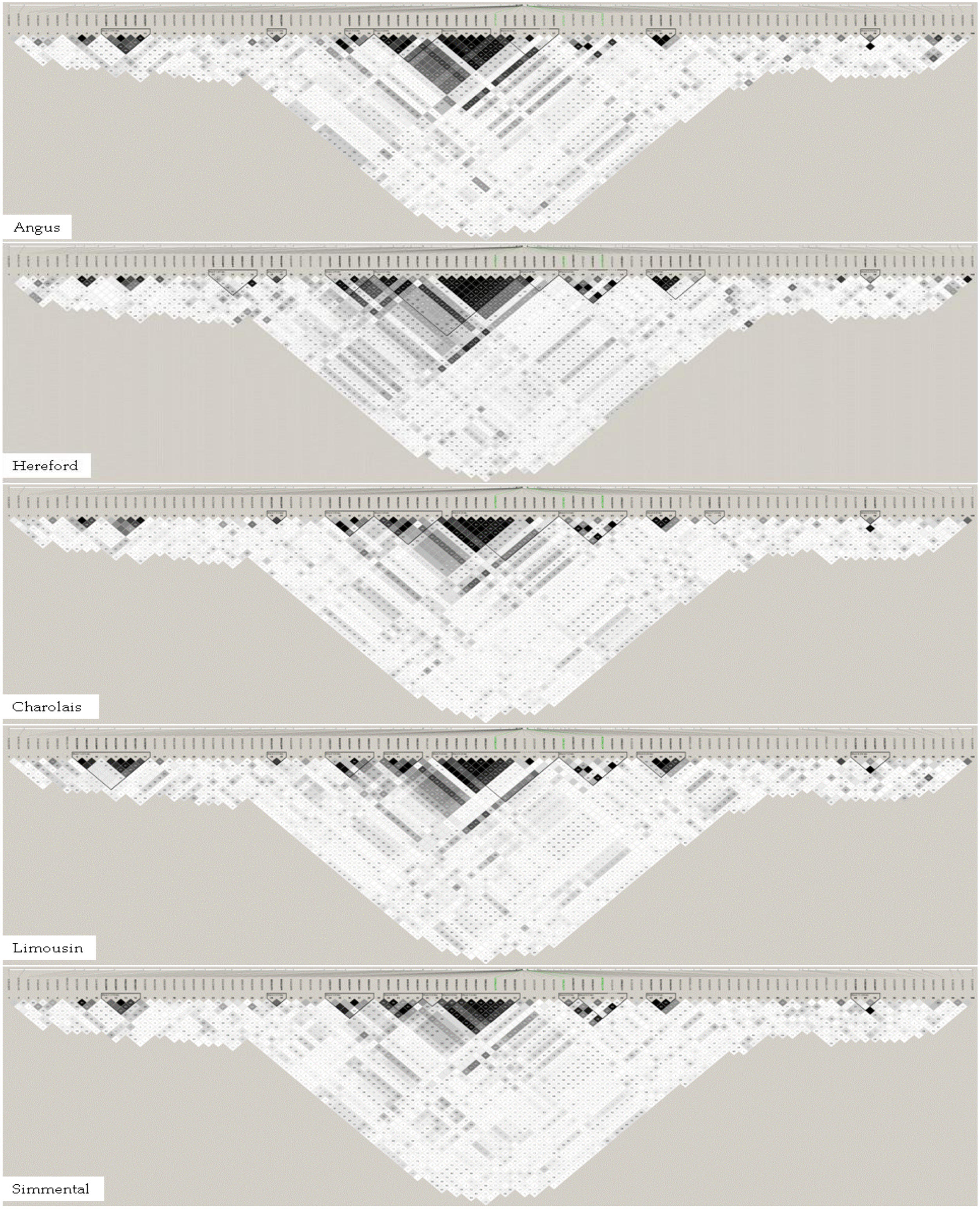

References

Barrett J.C., Fry B., Maller J. & Daly M.J. (2005) Haploview: analysis and visualization of LD and haplotype maps. *Bioinformatics* **21,** 263-5.
