# Supplementary material for: Genome-wide association analysis for quantitative trait loci influencing Warner–Bratzler shear force in five taurine cattle breeds
Source: Anim Genet. 2012 Feb 27;43(6):662–73. doi: 10.1111/j.1365-2052.2012.02323.x (PMC3506923; doi:10.1111/j.1365-2052.2012.02323.x)
Supplement: Table S1 — Characteristics of single-nucleotide polymorphisms located near calpastatin and calpain 1, (mu/I) large subunit that were designed into the Illumina GoldenGate assay and genotyped in 3240 CMP animals. [file age0043-0662-sd4.pdf]

Table S1 Characteristics of SNPs located near *CAST* and *CAPN1* that were designed into the Illumina GoldenGate assay and genotyped in 3,240 CMP animals

				Call Rate (%)					*A* Allele Frequency				
SNP ID[1]	BTA	POS[2]	Filtered[3]	Angus	Hereford	Charolais	Limousin	Simmental	Angus	Hereford	Charolais	Limousin	Simmental
rs42269174	7	98,260,103	AF	99.09	98.41	99.00	89.47	97.89	0.0000	0.0009	0.0000	0.0000	0.0000
rs42269169	7	98,346,128	AF	99.09	97.82	99.00	89.12	97.50	1.0000	0.9991	1.0000	0.9980	0.9990
rs42269168	7	98,351,749	CR,AF	93.48	92.03	96.58	88.42	95.20	0.9749	0.9699	0.9904	0.9782	0.9808
rs42269166	7	98,381,155	AF	98.79	97.57	99.00	90.18	97.89	0.0000	0.0000	0.0000	0.0039	0.0000
rs42269167	7	98,406,957	AF	98.64	96.90	99.15	91.23	98.46	0.0077	0.0074	0.0101	0.0058	0.0058
rs42269164	7	98,414,333	CR,AF	98.94	96.81	97.72	88.42	96.93	0.9985	0.9931	0.9942	0.9940	0.9851
rs42269165	7	98,423,052	CR,AF	98.94	97.57	98.72	88.77	97.70	0.0000	0.0000	0.0000	0.0000	0.0000
rs43529864	7	98,446,203	AF	98.94	97.57	98.86	89.82	97.89	0.0000	0.0000	0.0000	0.0000	0.0000
rs43529865	7	98,447,598	AF	98.64	97.06	99.00	89.12	97.31	1.0000	1.0000	1.0000	1.0000	1.0000
rs43529866	7	98,450,896	AF	99.24	98.57	99.15	89.82	97.89	1.0000	0.9991	1.0000	1.0000	1.0000
rs43529867	7	98,462,790	AF	99.09	98.07	98.86	90.18	98.27	0.0000	0.0000	0.0000	0.0000	0.0010
rs43529868	7	98,465,338	AF	98.94	97.48	98.29	89.12	97.89	1.0000	0.9991	1.0000	1.0000	0.9980
rs41596484	7	98,481,587	AF	99.09	97.90	98.86	89.82	97.50	0.0122	0.0043	0.0677	0.0137	0.0600
rs41596488	7	98,482,088	CR	99.09	97.32	98.43	88.77	97.31	0.7768	0.4767	0.7359	0.8241	0.5592
rs43529869	7	98,486,784	AF	98.18	97.06	98.72	90.18	97.12	0.0000	0.0000	0.0000	0.0000	0.0000
rs43529872	7	98,495,888		99.09	98.15	98.72	90.18	98.08	0.2064	0.5094	0.1912	0.1518	0.3630
rs43529870	7	98,503,071	CR,AF	95.15	77.10	93.02	89.82	84.84	0.9968	0.9717	0.9931	0.9961	0.9706
rs41619071	7	98,601,495	DNC										
rs43529862	7	98,525,723	AF	99.24	98.24	98.58	89.82	97.70	0.9992	0.9983	0.9993	1.0000	0.9951
rs41660170	7	98,538,952		99.09	97.06	99.15	90.18	98.08	0.0749	0.0402	0.1121	0.0856	0.0646
rs42776088	7	98,559,498	AF	99.09	95.72	98.86	89.82	97.50	1.0000	0.9982	1.0000	1.0000	0.9990
rs42776087	7	98,574,830	CR	98.94	96.73	98.29	88.77	96.35	0.2213	0.5108	0.2471	0.2688	0.4373
rs41255587	7	98,579,574		98.64	96.31	98.01	89.47	96.55	0.2166	0.5109	0.2406	0.2725	0.4205
rs42776090	7	98,620,447	AF	99.24	98.49	99.00	89.82	98.08	1.0000	0.9991	1.0000	0.9980	0.9980
rs41596489	7	98,646,770		99.24	98.15	98.72	89.12	97.70	0.4901	0.6970	0.6154	0.7756	0.6916
rs43529875	7	98,680,392	AF	99.09	98.07	98.58	89.12	98.46	1.0000	0.9987	1.0000	1.0000	0.9951
rs43529877	7	98,701,638	AF	99.24	98.57	99.15	89.82	98.27	0.0023	0.0060	0.0036	0.0059	0.0020
rs41657006	7	98,709,304	CR	93.94	78.69	85.33	85.61	55.09	0.3750	0.2655	0.3038	0.2111	0.2648
rs43101783	29	43,924,518	AF	98.79	97.65	97.72	89.47	96.93	0.9969	0.9987	0.9942	0.9902	0.9990
rs42191319	29	44,000,728		98.64	96.81	98.72	90.88	97.50	0.0630	0.2959	0.0938	0.0946	0.1358

rs42192038	29	44,029,526		98.33	97.99	98.58	89.47	97.31		0.4923	0.5801	0.3736	0.2137	0.3481
rs42192064	29	44,042,363		99.09	98.15	99.00	89.47	98.27		0.6284	0.4526	0.4187	0.3902	0.4014
rs42192074	29	44,047,246	CR	98.18	96.73	97.44	88.77	95.59		0.6420	0.6019	0.6235	0.5929	0.6235
rs42192077	29	44,049,244		98.64	97.48	98.29	89.12	97.50		0.3510	0.3972	0.3652	0.3878	0.3376
rs42192079	29	44,051,567		98.33	96.56	98.15	89.82	97.31		0.6456	0.6012	0.6277	0.5938	0.6282
rs42192083	29	44,053,733		99.24	97.57	97.72	90.18	96.93		0.3573	0.4015	0.3754	0.4027	0.3663
rs42192084	29	44,054,901		98.94	96.90	98.15	89.12	95.20		0.7940	0.7290	0.7736	0.7539	0.8115
rs41596485	29	44,054,902	CR	9.09	5.03	8.55	12.98	4.99		0.5000	0.4917	0.5000	0.4865	0.5000
rs42192090	29	44,058,688		98.79	98.15	99.00	89.47	98.08		0.4410	0.5679	0.4626	0.6412	0.4374
rs42192096	29	44,060,410		99.24	97.82	98.86	90.18	98.08		0.5534	0.4125	0.4697	0.2899	0.5548
rs42192100	29	44,062,694		98.79	97.23	98.01	89.82	96.74		0.5529	0.4142	0.4731	0.2910	0.5595
rs17872077	29	44,063,844	CR	87.12	84.90	91.03	79.30	85.99		0.1122	0.1260	0.0845	0.0664	0.0993
rs41657009	29	44,063,937	DNC							0.0000	0.0000	0.0000	0.0000	0.0000
rs17872078	29	44,063,938		98.94	96.98	98.72	90.18	97.70		0.4893	0.2171	0.3333	0.1868	0.3536
rs17872101	29	44,066,634	AF	99.09	98.49	99.00	90.18	98.46		0.0000	0.0000	0.0000	0.0000	0.0000
rs17871028	29	44,067,079	AF	99.24	96.98	98.86	89.82	97.31		0.0031	0.0078	0.0029	0.0000	0.0286
rs17872081	29	44,067,161	AF	98.48	97.90	98.43	89.82	97.89		0.0000	0.0004	0.0000	0.0000	0.0020
rs17872093	29	44,067,234		99.24	97.48	97.72	89.82	97.31		0.4466	0.5856	0.5313	0.7090	0.4438
rs17872094	29	44,067,596	CR	62.27	56.71	73.36	49.47	69.10		0.3917	0.6198	0.5427	0.4681	0.6153
rs17871984	29	44,067,796		99.24	97.65	98.58	90.18	98.46		0.1260	0.2302	0.2406	0.1498	0.3343
rs17870633	29	44,067,919	AF	98.94	98.07	98.86	90.18	97.31		0.0000	0.0004	0.0007	0.0000	0.0020
rs17871987	29	44,067,984	CR	99.24	97.73	98.72	88.77	97.50		0.4267	0.1833	0.2302	0.1403	0.2185
rs17870631	29	44,068,143		99.24	97.99	98.72	90.53	98.08		0.5725	0.8134	0.7691	0.8566	0.7789
rs17870630	29	44,068,247	AF	99.24	98.24	98.58	90.18	97.89		1.0000	0.9996	1.0000	1.0000	1.0000
rs17870628	29	44,068,346		98.94	97.48	98.01	89.47	97.89		0.5352	0.8081	0.7071	0.7902	0.7010
rs17870626	29	44,068,445		99.09	97.82	98.72	90.18	96.55		0.5680	0.8135	0.7655	0.8521	0.7465
rs17872010	29	44,068,519		98.94	97.23	97.72	90.18	97.31		0.5743	0.8162	0.7682	0.8560	0.7761
rs17872004	29	44,068,580		98.33	97.40	98.29	90.18	95.97		0.5732	0.8178	0.7703	0.8541	0.7820
rs17872003	29	44,068,812		97.58	96.39	98.15	89.82	96.93		0.4278	0.1819	0.2315	0.1445	0.2188
rs17872000	29	44,069,063		98.18	95.72	99.43	99.30	99.23						
rs17872006	29	44,069,177		96.21	97.23	97.86	89.47	97.12		0.4299	0.1877	0.2394	0.1451	0.2322
rs42192102	29	44,070,590	CR	98.79	97.23	98.15	87.72	97.70		0.4371	0.5669	0.5203	0.6860	0.3978
rs42192103	29	44,070,713		96.67	95.81	98.29	89.82	96.55		0.4326	0.1988	0.2355	0.1641	0.2674
rs42192105	29	44,070,881		98.79	97.57	97.72	89.47	97.31		0.1304	0.1561	0.0707	0.0431	0.0966

rs42192107	29	44,071,034		98.94	97.48	98.72	90.53	97.70		0.1187	0.1506	0.0592	0.0426	0.0452
rs42192108	29	44,071,135		99.09	97.32	98.43	89.82	97.70		0.0344	0.1858	0.1606	0.0781	0.0472
rs42192109	29	44,071,593		98.79	96.90	98.58	89.47	97.31		0.4954	0.3935	0.3728	0.2667	0.4043
rs42192110	29	44,071,657	CR	99.24	97.65	98.15	88.42	97.89		0.5595	0.4214	0.4768	0.3056	0.5500
rs42192111	29	44,071,828	CR,AF	74.24	63.17	60.97	44.91	63.34		1.0000	0.9967	1.0000	0.9844	0.9970
rs42192117	29	44,081,264	AF	98.18	97.23	98.86	89.82	97.50		0.0000	0.0000	0.0000	0.0000	0.0010
rs17870499	29	44,085,195	CR	98.64	97.40	98.58	88.07	97.70		0.0499	0.0263	0.0874	0.1096	0.0697
rs17871051	29	44,085,642		99.39	97.65	98.29	89.12	97.50		0.0998	0.1044	0.3659	0.3996	0.3051
rs17871058	29	44,085,769	DNC*											
rs17871060	29	44,086,042	AF	99.24	98.07	98.29	89.82	96.55		1.0000	1.0000	1.0000	0.9961	1.0000
rs17872027	29	44,086,761	AF	99.24	97.82	99.00	90.53	97.89		0.0061	0.1677	0.0259	0.0174	0.0216
rs17872030	29	44,086,943	CR	98.64	97.40	98.29	88.42	96.35		0.0975	0.1047	0.3616	0.4028	0.3058
rs17872031	29	44,087,019	AF	94.70	97.82	96.58	90.53	98.27		0.0160	0.0262	0.0133	0.0000	0.0078
rs17872032	29	44,087,205		98.33	97.32	98.15	90.18	96.93		0.0493	0.0250	0.0842	0.0953	0.0683
rs17872033	29	44,087,356		97.58	96.31	97.58	89.82	95.39		0.8960	0.8916	0.6263	0.5938	0.6620
rs17872050	29	44,087,629		99.55	96.64	99.29	99.65	99.04						
rs17872151	29	44,087,858		99.09	97.90	98.15	90.18	97.70		0.0153	0.0167	0.0856	0.1323	0.0452
rs17872153	29	44,088,027	CR	99.09	97.65	98.01	87.72	96.93		0.9893	0.9854	0.8990	0.8360	0.9010
rs17870855	29	44,088,451	CR	98.79	97.15	98.43	88.77	96.93		0.0997	0.1036	0.3647	0.4071	0.3267
rs17870847	29	44,088,897		99.24	97.23	98.72	90.53	98.08		0.7038	0.8352	0.4531	0.3217	0.5284
rs17870848	29	44,089,043	CR	88.94	91.78	91.31	81.05	91.55		0.6457	0.5457	0.6131	0.5714	0.5461
rs42192119	29	44,110,295		99.24	98.24	98.86	89.82	98.08		0.3267	0.4539	0.4150	0.4219	0.3591
rs42192125	29	44,112,272	AF	99.09	97.73	99.15	90.88	98.46		0.0000	0.0000	0.0000	0.0019	0.0010
rs42195143	29	44,119,671		96.82	96.31	98.29	90.18	96.35		0.5939	0.6224	0.6203	0.5233	0.6365
rs42194216	29	44,122,272		99.09	98.07	98.86	89.82	97.70		0.0956	0.1737	0.0620	0.1543	0.1130
rs42194178	29	44,125,747		99.55	98.07	98.86	90.88	97.89		0.0951	0.1698	0.0641	0.1544	0.1118
rs42194132	29	44,130,887		99.09	97.15	99.15	89.12	97.70		0.9060	0.8286	0.9368	0.8465	0.8870
rs42193349	29	44,154,126		98.18	97.32	98.01	89.12	97.50		0.0332	0.1586	0.0334	0.1240	0.0827
rs42193335	29	44,163,955	AF	99.09	97.99	98.72	89.82	97.31		1.0000	0.9991	1.0000	0.9961	1.0000
rs41586223	29	44,196,154	DNC											
rs29003633	29	44,208,978		98.94	97.99	99.00	90.18	97.89		0.0398	0.0424	0.0540	0.0136	0.0480
rs42195220	29	44,235,914	AF	99.24	98.49	99.15	90.53	98.27		0.0000	0.0000	0.0000	0.0000	0.0000

[1]Identities of commercialized SNPs predictive of tenderness are shaded in red. Identities of SNPs within *CAST* and *CAPN1* are shaded in yellow.

Bold SNPs have been removed from dbSNP.

[2] Base pair position from the UMD3.1 sequence assembly.									
[3] Reason for filtering SNP from analysis. DNC=Did not convert, AF=Allele Frequency, CR=Call rate. * = Also included on BovineSNP50 assay.									
