# Supplementary material for: Genome-wide association analysis for quantitative trait loci influencing Warner–Bratzler shear force in five taurine cattle breeds
Source: Anim Genet. 2012 Feb 27;43(6):662–73. doi: 10.1111/j.1365-2052.2012.02323.x (PMC3506923; doi:10.1111/j.1365-2052.2012.02323.x)
Supplement: Table S3 — Patterns of single-nucleotide polymorphism association with Warner–Bratzler shear force for calpastatin and calpain 1, (mu/I) large subunit loci. [file age0043-0662-sd6.pdf]

Table S3. Patterns of SNP association with WBSF for *CAST* and *CAPN1* loci.

					Rank[3]			
BTA	POS[1]	SNP ID[2]	All Breeds	Angus	Hereford	Charolais	Limousin	Simmental
7	97,151,592	*rs41656985*	17853	22832	22123	11997	40460	19009
7	97,261,052	*rs41656987*	654	5620	39476	13999	1891	2069
7	97,372,656	*rs41570922*	20417	16753	26124	38433	14071	33362
7	97,393,157	*rs41615915*	1427	37659	39656	1009	17193	6660
7	97,430,691	*ss86295638*	3045	39136	27527	1047	23654	13621
7	97,501,859	*ss86310801*	13413	33096	22602	19582	10997	16395
7	97,529,872	*ss61472319*	10706	16140	20625	1743	6464	29536
7	97,691,853	*ss86290016*	30824	31090	22986	36148	36108	24197
7	97,772,240	*ss86312419*	10263	8991	28755	7170	16285	37994
7	97,861,341	*rs41593284*	921	27589	4833	32544	37	17064
7	97,898,940	*rs41593281*	50	10852	33	23180	32882	6820
7	97,961,659	*ss117969141*	28454	25196	38529	39653	37599	25297
7	98,013,150	*ss117968909*	35590	29216	1170	4574	26692	308
7	98,173,299	*ss86290989*	30594	29793	9717	4845	29926	29007
7	98,194,828	*rs41658549*	34336	6424	2534	90	30247	15824
7	98,254,815	*rs41658542*	13991	24584	267	603	11619	21439
7	98,375,640	*ss86329349*	1790	4605	1593	38596	23512	33940
7	98,495,888	*rs43529872*	13	610	3	57	3069	20634
7	98,498,047	*ss86321563*	12	294	2	66	6102	15068
7	98,538,952	*rs41660170*	8319	6163	39936	10548	29756	20353
7	98,566,391	*ss117962527*	1310	2288	27900	619	20424	39851
7	98,579,574	*rs41255587*	10	237	1	14	2479	27896
7	98,635,208	*ss86300200*	37	11749	7	11342	4871	8383
7	98,646,770	*rs41596489*	34	628	11	38675	17319	30575
7	98,680,293	*ss86315737*	250	465	34	18680	14562	39284
7	98,726,055	*rs29018251*	13126	848	5956	5486	38597	28483
7	98,757,265	*ss86303738*	17394	26602	27914	20844	34152	5174
7	98,820,742	*ss86324113*	137	746	150	29169	22220	2224
7	98,887,313	*rs41568595*	15241	35496	29107	8018	38017	32238
7	98,907,404	*ss86291843*	9022	39450	9020	20771	4773	22927
7	98,974,456	*ss86339659*	7758	16220	6313	25941	24786	34105
7	99,024,444	*ss117968912*	24262	20693	6213	1798	15823	13240
7	99,085,299	*rs41590403*	9428	20682	24941	9134	4854	30656
7	99,130,598	*ss86333606*	1878	35186	26	34124	33105	11400
7	99,178,748	*rs41568774*	33934	35119	17840	37290	16533	18785
7	99,215,474	*ss86324013*	5728	38209	1565	37408	34561	25708
7	99,344,820	*rs41622872*	1488	20805	292	22465	1993	7618
7	99,399,852	*ss117968895*	37103	18064	39777	21174	35095	37490
7	99,492,904	*ss86296856*	6124	5452	40099	5651	36078	36085
7	99,635,841	*ss86339853*	32326	35802	7339	10001	11279	8481
7	99,687,614	*rs41657025*	19734	6764	15594	21139	38911	6044
7	99,845,133	*rs41624339*	1593	28933	13874	1849	16503	9618
7	99,919,908	*ss86339607*	35826	32733	12431	40446	13228	18950
7	100,015,468	*ss86310226*	27936	27227	9309	36010	12692	15892
29	42,406,544	*ss86319674*	37050	4925	23582	28260	21027	18936
29	42,455,680	*ss117974470*	22297	37162	29770	25573	5930	7326
29	42,620,218	*rs42189770*	35226	28646	8272	20924	33490	30054
29	42,651,294	*rs43706142*	28368	1526	13688	20292	33318	33867
29	42,696,595	*rs42189112*	11135	4128	567	12339	38243	4930
29	42,749,808	*rs29020063*	28224	13151	2449	29252	12285	1538
29	42,842,353	*ss117974486*	8500	5777	7774	25144	6122	24
29	42,897,144	*ss86322358*	27082	14890	31939	27607	33377	7184

29	43,006,000	*ss86339964*	32865	10952	32290	27608	33378	10290
29	43,043,207	*ss86341572*	20145	5329	32350	4763	12546	3676
29	43,108,445	*ss86337140*	608	1800	3230	19455	27355	8204
29	43,129,250	*ss86337549*	7614	933	38609	37467	9721	22158
29	43,189,370	*ss86319044*	9896	929	38610	39410	15557	22159
29	43,224,753	*rs29013208*	9469	1268	37486	27816	15836	8935
29	43,269,744	*ss86282762*	16588	2397	36965	25543	21052	24438
29	43,304,256	*rs29024708*	5161	1726	19477	22317	32243	344
29	43,328,607	*ss86295061*	35423	2869	9694	11234	25778	26570
29	43,429,511	*ss86318094*	42	66	37	20269	10690	7322
29	43,466,342	*ss86310741*	7210	889	20819	12113	6737	11755
29	43,498,073	*ss86338800*	1179	1059	26456	1057	12237	4447
29	43,611,640	*ss86298040*	77	13108	2175	227	27133	175
29	43,652,252	*ss86327310*	309	28	6692	33574	14789	1316
29	43,686,401	*ss86325323*	30559	1290	10319	11271	38311	16973
29	43,709,769	*ss86290081*	6403	191	4662	9805	15489	17693
29	43,747,765	*rs42190085*	9036	19561	38491	8579	26434	27476
29	43,749,527	*ss86299346*	25366	23606	37713	17640	33965	21036
29	43,777,249	*ss86299989*	35438	2116	35762	18707	39614	29763
29	43,826,144	*rs42191289*	31815	3320	9684	22649	7809	192
29	43,839,783	*ss86336460*	5396	729	9980	6073	17672	49
29	43,914,923	*ss86335514*	24	6410	3157	114	8553	102
29	44,000,728	*rs42191319*	22471	40614	11851	25045	22311	31987
29	44,029,526	*rs42192038*	1251	6182	24398	4384	19663	609
29	44,042,363	*rs42192064*	21	182	3321	11547	31563	1
29	44,049,244	*rs42192077*	8485	17117	1831	29917	40623	33046
29	44,050,471	*ss86284058*	3336	26458	559	8623	33356	13451
29	44,051,567	*rs42192079*	11270	22597	2795	29554	39615	31917
29	44,053,733	*rs42192083*	12323	17992	2555	32354	36798	30606
29	44,054,901	*rs42192084*	22522	24469	12212	13490	30189	18346
29	44,058,688	*rs42192090*	6685	2065	17751	201	10644	9852
29	44,060,410	*rs42192096*	7985	1898	10671	887	17953	3893
29	44,062,694	*rs42192100*	8555	1844	10835	1333	20163	4552
29	44,063,938	*rs17872078*	8	12	36	15	29894	25
29	44,067,234	*rs17872093*	10292	1899	6796	888	20164	3580
29	44,067,796	*rs17871984*	11	44	4	2066	19038	516
29	44,068,143	*rs17870631*	2	4	24	7	39018	6
29	44,068,346	*rs17870628*	14	16	19	750	22524	269
29	44,068,445	*rs17870626*	6	7	29	12	31903	5
29	44,068,519	*rs17872010*	7	6	40	8	39019	13
29	44,068,580	*rs17872004*	4	5	27	11	36321	9
29	44,068,812	*rs17872003*	5	3	28	6	35508	12
29	44,069,063	*rs17812000*	3	1	59	9	39020	10
29	44,069,177	*rs17872006*	9	2	46	40	24824	14
29	44,070,713	*rs42192103*	1	8	9	1	25633	2
29	44,070,881	*rs42192105*	394	6164	200	30967	37715	10226
29	44,071,034	*rs42192107*	495	5948	183	22528	37716	21810
29	44,071,135	*rs42192108*	10891	35181	23330	21578	9516	38169
29	44,071,593	*rs42192109*	1089	193	26124	100	11867	2864
29	44,085,642	*rs17871051*	22	22	8993	829	3823	128
29	44,085,769	*rs17871058*	16	29	10865	5	82	44
29	44,087,205	*rs17872032*	977	3035	36234	2335	14676	3236
29	44,087,356	*rs17872033*	27	26	9090	1020	2073	337
29	44,087,629	*rs17872050*	15	17	11193	3	30	54
29	44,087,858	*rs17872151*	7794	38670	21318	12661	22722	19232

29	44,088,897	*rs17870847*	17	23	13127	2	39	91
29	44,110,295	*rs42192119*	24761	30393	34998	11139	17791	29587
29	44,119,671	*rs42195143*	27184	13786	2181	33844	29050	24476
29	44,122,272	*rs42194216*	17667	28444	8083	27006	31593	40203
29	44,125,747	*rs42194178*	16874	25410	6562	27074	31594	40204
29	44,130,887	*rs42194132*	20944	25411	7613	26871	31592	31199
29	44,154,126	*rs42193349*	13422	10208	5641	31876	28607	21748
29	44,172,614	*ss117965984*	3742	39565	4851	10280	24312	5842
29	44,196,154	*ss86318958*	29029	13994	8832	22414	34749	33087
29	44,208,978	*rs29003633*	19817	1795	39702	33937	39148	28356
29	44,223,148	*ss86323783*	1050	105	36704	940	9476	28615
29	44,243,444	*ss86313099*	32174	543	399	4467	31547	19210
29	44,325,408	*ss86292140*	41	11	14276	2309	15992	648
29	44,372,611	*ss86329852*	15417	38369	20426	2699	9819	8295
29	44,395,075	*ss86333994*	275	979	2714	33693	22902	210
29	44,416,282	*rs43709648*	18	149	10	1683	32992	14042
29	44,546,564	*ss86288185*	494	1548	1310	4321	35230	12565
29	44,585,782	*ss86341172*	2967	2916	9784	1728	16607	18099
29	44,628,137	*rs41600300*	35992	11176	30882	26481	13878	19086
29	44,649,908	*ss86337530*	36291	30728	2567	18016	29243	4361
29	44,740,917	*ss86334185*	2424	652	2681	15788	26662	24049
29	44,807,928	*ss117965824*	20185	804	1305	37934	36739	26103
29	44,853,970	*rs42192429*	36290	112	19416	4118	21464	2705
29	44,900,940	*rs43706228*	1504	9	26793	43	35156	24021
29	44,969,518	*ss86337154*	21170	31013	35635	16801	23994	26608
29	44,979,377	*ss86338214*	24112	9928	13777	19335	30736	9711
29	44,999,264	*ss86310727*	143	1078	32294	2467	2164	425
29	45,023,665	*ss86338763*	25485	31014	30793	17900	23995	28945
29	45,102,557	*ss86332221*	170	2240	31730	75	31673	264
29	45,129,099	*ss86335405*	4960	2025	26978	5490	33645	24448
29	45,187,114	*ss86295760*	9892	468	16200	33586	24838	32136
29	45,287,502	*ss86312485*	7710	6287	36387	1294	30142	35470
29	45,326,585	*rs42198683*	23963	15047	6206	174	30801	14249
29	45,367,095	*ss86322638*	13328	12392	3940	22784	19051	6856
29	45,458,280	*rs43706176*	24499	6324	34840	34884	24588	40236
29	45,482,143	*rs29025626*	1893	136	35054	17005	2280	16790
29	45,530,264	*ss86341375*	34109	39453	35889	20334	18804	24973

[1]UMD3.1 Coordinates. SNPs located within *CAST* and *CAPN1* are shaded in yellow.

[2]Commercialized SNPs are shaded in red.

[3]GBLUP allele substitution effect (α) ranks. Values less than 500 are shaded in yellow for the across-breed

[4]GBLUP of SNP allele substitution effects (α) estimated as a random effect.

[5]SNP allele substitution effects (β) estimated as fixed effects in a mixed linear model including the genom

[6]-$\log_{10}$(P)-value for the test of the null hypothesis of an allele substitution effect (β) of zero. Maximum va

[7]-$\log_{10}$(P)-value for the test of the null hypothesis of all haplotype substitution effects (H) being zero. Ma

[8]Number of haplotypes and effective number of haplotypes in parentheses for the 13 (*CAST*) or 31 (*CAPN*

[9]Number Percentage of phenotypic variation in WBSF explained by haplotypes centered on the identified

						ASE[4]		
BTA	POS[1]	SNP ID[2]	α-All Breeds	α-Angus	α-Hereford	α-Charolais	α-Limousin	α-Simmental
7	97,151,592	*rs41656985*	0.00030671	-0.00017099	0.00020134	0.00017805	-0.00000025	0.00003928
7	97,261,052	*rs41656987*	0.00104868	0.00048033	0.00017963	-0.00001059	0.00011002	0.00011454
7	97,372,656	*rs41570922*	-0.00026557	-0.00024736	0.00001186	-0.00013418	-0.00004761	0.00001181
7	97,393,157	*rs41615915*	-0.00090198	0.00002637	-0.00048107	0.00000907	-0.00003997	-0.00007934
7	97,430,691	*ss86295638*	0.00074568	-0.00001324	0.00047692	-0.00012040	-0.00002678	0.00005340
7	97,501,859	*ss86310801*	-0.00039152	-0.00006694	-0.00012889	-0.00017279	0.00005665	-0.00004583
7	97,529,872	*ss61472319*	0.00045326	-0.00025686	0.00042590	-0.00019590	0.00007461	-0.00001851
7	97,691,853	*ss86290016*	0.00012060	0.00008623	0.00002409	0.00016817	0.00000658	-0.00002851
7	97,772,240	*ss86312419*	-0.00046509	-0.00038513	-0.00026961	-0.00010804	0.00004202	0.00000432
7	97,861,341	*rs41593284*	0.00097782	0.00011997	0.00031705	-0.00007204	0.00019796	0.00004403
7	97,898,940	*rs41593281*	0.00155220	0.00034795	0.00080756	0.00016613	0.00001148	0.00007858
7	97,961,659	*ss117969141*	0.00015075	0.00014493	0.00001131	0.00000912	0.00000444	0.00002638
7	98,013,150	*ss117968909*	0.00006102	-0.00010369	-0.00046716	0.00050154	0.00002137	0.00016343
7	98,173,299	*ss86290989*	0.00012363	0.00009822	-0.00022941	0.00049209	0.00001609	0.00001944
7	98,194,828	*rs41658549*	-0.00007628	-0.00045406	0.00038728	-0.00109154	0.00001558	0.00004740
7	98,254,815	*rs41658542*	0.00037965	-0.00015143	0.00060092	-0.00081707	-0.00005474	0.00003396
7	98,375,640	*ss86329349*	0.00085501	0.00051810	0.00043447	0.00001862	0.00002704	-0.00001097
7	98,495,888	*rs43529872*	0.00250806	0.00083827	0.00110064	0.00117126	0.00009663	-0.00003567
7	98,498,047	*ss86321563*	0.00251152	0.00094639	0.00113500	0.00114492	0.00007645	-0.00004943
7	98,538,952	*rs41660170*	0.00051614	0.00046201	-0.00000394	0.00034683	0.00001636	0.00003628
7	98,566,391	*ss117962527*	-0.00091729	-0.00063906	-0.00007139	-0.00081305	-0.00003279	0.00000133
7	98,579,574	*rs41255587*	0.00282846	0.00097459	0.00114941	0.00135049	0.00010269	-0.00002145
7	98,635,208	*ss86300200*	-0.00163961	-0.00033048	-0.00094817	0.00033141	-0.00008345	-0.00007121
7	98,646,770	*rs41596489*	0.00165452	0.00083405	0.00091620	0.00001794	0.00003965	0.00001667
7	98,680,293	*ss86315737*	-0.00122381	-0.00087793	-0.00080722	0.00022035	0.00004635	-0.00000223
7	98,726,055	*rs29018251*	0.00039725	-0.00079091	0.00029255	0.00047142	0.00000296	0.00002039
7	98,757,265	*ss86303738*	0.00031484	-0.00013005	0.00007134	0.00019325	-0.00000945	0.00008748
7	98,820,742	*ss86324113*	0.00133604	0.00080850	0.00064476	-0.00010404	-0.00002931	0.00011256
7	98,887,313	*rs41568595*	-0.00035467	0.00004527	-0.00006420	-0.00040082	0.00000383	-0.00001382
7	98,907,404	*ss86291843*	-0.00049765	-0.00001049	-0.00023992	-0.00019419	-0.00008403	0.00003096
7	98,974,456	*ss86339659*	0.00053311	0.00025554	0.00028576	0.00013611	0.00002468	0.00001069
7	99,024,444	*ss117968912*	-0.00020917	-0.00019566	0.00028752	-0.00066185	0.00004319	-0.00005451
7	99,085,299	*rs41590403*	-0.00048724	-0.00019597	0.00009020	-0.00037474	-0.00008357	-0.00001654
7	99,130,598	*ss86333606*	-0.00084381	0.00004792	-0.00082834	0.00005799	0.00001115	0.00006039
7	99,178,748	*rs41568774*	-0.00008141	-0.00004854	0.00014285	-0.00003014	-0.00004148	-0.00003983
7	99,215,474	*ss86324013*	0.00060578	0.00002168	0.00043622	-0.00002909	0.00000885	-0.00002556
7	99,344,820	*rs41622872*	-0.00089371	0.00019432	-0.00059364	0.00017413	-0.00010856	-0.00007464
7	99,399,852	*ss117968895*	0.00004348	-0.00022947	0.00011675	-0.00000801	-0.00000804	-0.00000512
7	99,492,904	*ss86296856*	-0.00059036	-0.00048595	-0.00029855	-0.00000510	-0.00000663	0.00000746
7	99,635,841	*ss86339853*	0.00010124	0.00004271	0.00022532	-0.00041791	0.00005579	-0.00007081
7	99,687,614	*rs41657025*	0.00027601	-0.00044383	0.00011698	0.00026276	-0.00000252	0.00008263
7	99,845,133	*rs41624339*	0.00087822	0.00010669	0.00041919	0.00028876	-0.00004153	0.00006660
7	99,919,908	*ss86339607*	0.00005831	-0.00007035	-0.00000100	0.00031240	0.00004996	-0.00003941
7	100,015,468	*ss86310226*	-0.00015773	0.00012360	-0.00002482	-0.00037122	0.00005151	-0.00004723
29	42,406,544	*ss86319674*	0.00004412	0.00050562	-0.00006947	-0.00016157	0.00003160	-0.00003943
29	42,455,680	*ss117974470*	-0.00023668	-0.00003069	0.00008615	0.00009795	-0.00007734	-0.00007606
29	42,620,218	*rs42189770*	0.00006534	0.00010962	-0.00011858	0.00039419	-0.00001053	-0.00001761
29	42,651,294	*rs43706142*	-0.00015187	-0.00070089	0.00012322	-0.00029180	0.00001081	0.00001108
29	42,696,595	*rs42189112*	0.00044366	0.00053810	-0.00019729	0.00082689	-0.00000348	0.00008903
29	42,749,808	*rs29020063*	-0.00015364	0.00030458	0.00006338	-0.00061076	0.00005270	-0.00012279
29	42,842,353	*ss117974486*	-0.00051136	-0.00047485	0.00025958	0.00014454	-0.00007630	-0.00023417
29	42,897,144	*ss86322358*	0.00016974	-0.00027606	0.00004743	0.00011962	-0.00001069	0.00007674

29	43,006,000	*ss86339964*		0.00009489	-0.00034623	0.00004546	0.00011962	-0.00001069	0.00006409
29	43,043,207	*ss86341572*		0.00026966	-0.00049034	0.00004519	0.00049483	-0.00005195	0.00009832
29	43,108,445	*ss86337140*		-0.00106080	-0.00067556	-0.00036196	-0.00021056	-0.00002031	-0.00007203
29	43,129,250	*ss86337549*		-0.00053788	-0.00077763	-0.00001082	-0.00002855	-0.00006132	-0.00003243
29	43,189,370	*ss86319044*		-0.00047528	-0.00077860	-0.00001082	-0.00001117	-0.00004383	-0.00003243
29	43,224,753	*rs29013208*		0.00048618	0.00073297	-0.00001707	-0.00011758	0.00004316	0.00006908
29	43,269,744	*ss86282762*		0.00032969	0.00063292	-0.00001988	-0.00014031	0.00003155	0.00002805
29	43,304,256	*rs29024708*		-0.00062896	-0.00068295	0.00012981	-0.00017576	-0.00001246	-0.00016043
29	43,328,607	*ss86295061*		0.00006312	0.00060219	-0.00022979	0.00033339	0.00002294	-0.00002393
29	43,429,511	*ss86318094*		-0.00160847	-0.00115096	-0.00079603	0.00019993	-0.00005774	-0.00007607
29	43,466,342	*ss86310741*		-0.00055100	-0.00078488	-0.00011944	-0.00031762	0.00007341	-0.00005915
29	43,498,073	*ss86338800*		0.00093454	0.00075831	0.00008009	0.00073932	-0.00005284	0.00009236
29	43,611,640	*ss86298040*		0.00146282	0.00030516	0.00040305	0.00095462	-0.00002067	0.00017820
29	43,652,252	*ss86327310*		-0.00118582	-0.00130334	-0.00027823	-0.00006298	-0.00004581	-0.00012680
29	43,686,401	*ss86325323*		-0.00012415	-0.00073056	-0.00022126	0.00033263	0.00000339	0.00004428
29	43,709,769	*ss86290081*		-0.00057947	-0.00100397	-0.00032111	0.00036105	0.00004399	-0.00004252
29	43,747,765	*rs42190085*		-0.00049735	-0.00020965	0.00001155	-0.00038731	-0.00002178	-0.00002225
29	43,749,527	*ss86299346*		-0.00019238	0.00016220	0.00001596	-0.00023397	-0.00000979	-0.00003480
29	43,777,249	*ss86299989*		-0.00006297	-0.00065114	-0.00002619	0.00022008	0.00000151	-0.00001811
29	43,826,144	*rs42191289*		0.00010758	0.00057745	-0.00022997	-0.00017228	-0.00006867	0.00017586
29	43,839,783	*ss86336460*		0.00061942	0.00081131	-0.00022565	0.00045409	-0.00003884	0.00020882
29	43,914,923	*ss86335514*		0.00178742	0.00045442	0.00036472	0.00105531	0.00006568	0.00019156
29	44,000,728	*rs42191319*		-0.00023431	0.00000024	-0.00020306	0.00014559	-0.00002916	0.00001428
29	44,029,526	*rs42192038*		-0.00092400	-0.00046153	-0.00009380	-0.00050894	-0.00003445	-0.00014690
29	44,042,363	*rs42192064*		-0.00185121	-0.00101148	-0.00035929	-0.00032758	0.00001350	-0.00033390
29	44,049,244	*rs42192077*		0.00051167	-0.00024213	0.00042010	0.00009658	-0.00000003	-0.00001236
29	44,050,471	*ss86284058*		-0.00072640	0.00013171	-0.00053768	-0.00038629	-0.00001074	0.00005388
29	44,051,567	*rs42192079*		-0.00044039	0.00017368	-0.00037741	-0.00009999	-0.00000151	0.00001440
29	44,053,733	*rs42192083*		0.00041510	-0.00023030	0.00038673	0.00007379	0.00000561	-0.00001662
29	44,054,901	*rs42192084*		0.00023354	-0.00015260	0.00019889	0.00029494	0.00001567	-0.00004086
29	44,058,688	*rs42192090*		0.00056930	0.00065475	-0.00014365	0.00097619	-0.00005793	0.00006581
29	44,060,410	*rs42192096*		-0.00052571	-0.00066766	0.00021685	-0.00076421	0.00003819	-0.00009649
29	44,062,694	*rs42192100*		-0.00050977	-0.00067190	0.00021482	-0.00070708	0.00003338	-0.00009162
29	44,063,938	*rs17872078*		-0.00295459	-0.00157336	-0.00080065	-0.00130368	-0.00001613	-0.00023251
29	44,067,234	*rs17872093*		0.00046438	0.00066766	-0.00027634	0.00076421	-0.00003338	0.00009915
29	44,067,796	*rs17871984*		0.00259095	0.00120226	0.00104514	0.00063915	0.00003574	0.00015159
29	44,068,143	*rs17870631*		0.00317238	0.00186992	0.00083717	0.00140771	0.00000236	0.00025628
29	44,068,346	*rs17870628*		0.00242209	0.00143725	0.00086741	0.00078922	-0.00002876	0.00016684
29	44,068,445	*rs17870626*		0.00308636	0.00178329	0.00081694	0.00138681	-0.00001299	0.00025696
29	44,068,519	*rs17872010*		0.00304194	0.00181486	0.00079066	0.00140771	0.00000236	0.00024530
29	44,068,580	*rs17872004*		0.00313874	0.00186992	0.00082829	0.00139445	0.00000629	0.00024985
29	44,068,812	*rs17872003*		-0.00312101	-0.00188257	-0.00082610	-0.00143916	0.00000748	-0.00024616
29	44,069,063	*rs17812000*		-0.00316072	-0.00228185	-0.00073880	-0.00140771	-0.00000236	-0.00024894
29	44,069,177	*rs17872006*		-0.00290630	-0.00194198	-0.00076244	-0.00120636	0.00002461	-0.00024429
29	44,070,713	*rs42192103*		-0.00332608	-0.00173409	-0.00094128	-0.00152269	0.00002317	-0.00028201
29	44,070,881	*rs42192105*		-0.00114491	-0.00046199	-0.00062239	-0.00008699	0.00000425	-0.00006435
29	44,071,034	*rs42192107*		-0.00110210	-0.00046869	-0.00062781	-0.00017354	0.00000425	-0.00003314
29	44,071,135	*rs42192108*		-0.00044909	-0.00004798	-0.00010125	-0.00018521	-0.00006208	-0.00000408
29	44,071,593	*rs42192109*		-0.00094890	-0.00100318	0.00008230	-0.00107584	0.00005396	-0.00010545
29	44,085,642	*rs17871051*		0.00181650	0.00134971	0.00024034	0.00077510	0.00009067	0.00018604
29	44,085,769	*rs17871058*		0.00236870	0.00130304	0.00021437	0.00146508	0.00018454	0.00021263
29	44,087,205	*rs17872032*		0.00096858	0.00059348	0.00002368	0.00061874	0.00004608	0.00010203
29	44,087,356	*rs17872033*		-0.00172481	-0.00132279	-0.00023887	-0.00074441	-0.00010755	-0.00016116
29	44,087,629	*rs17872050*		0.00241788	0.00143400	0.00021062	0.00148598	0.00019989	0.00020641
29	44,087,858	*rs17872151*		0.00053210	-0.00001758	0.00011545	0.00030835	0.00002839	0.00003877

29	44,088,897	*rs17870847*		-0.00236640	-0.00134853	-0.00018849	-0.00152169	-0.00019782	-0.00019442
29	44,110,295	*rs42192119*		-0.00020159	0.00009268	-0.00003017	-0.00033493	-0.00003855	0.00001843
29	44,119,671	*rs42195143*		0.00016832	-0.00029353	0.00040275	-0.00006054	0.00001746	-0.00002797
29	44,122,272	*rs42194216*		-0.00031009	0.00011168	-0.00025424	-0.00012528	0.00001347	-0.00000075
29	44,125,747	*rs42194178*		-0.00032379	0.00014293	-0.00028060	-0.00012467	0.00001347	-0.00000075
29	44,130,887	*rs42194132*		0.00025708	-0.00014293	0.00026202	0.00012655	-0.00001347	-0.00001560
29	44,154,126	*rs42193349*		-0.00039133	0.00036022	-0.00029871	-0.00007837	0.00001820	-0.00003331
29	44,172,614	*ss117965984*		-0.00069936	0.00000948	-0.00031668	-0.00035165	0.00002555	-0.00008372
29	44,196,154	*ss86318958*		0.00014359	0.00029049	0.00024258	-0.00017471	0.00000858	-0.00001230
29	44,208,978	*rs29003633*		0.00027473	0.00067584	0.00000504	0.00005976	-0.00000215	-0.00002063
29	44,223,148	*ss86323783*		0.00095480	0.00108636	0.00002128	0.00075592	0.00006222	0.00002016
29	44,243,444	*ss86313099*		-0.00010319	0.00085706	-0.00056913	0.00050513	0.00001353	-0.00003883
29	44,325,408	*ss86292140*		-0.00161978	-0.00161585	-0.00017661	-0.00061998	-0.00004278	-0.00014539
29	44,372,611	*ss86329852*		-0.00035147	0.00002025	-0.00012224	-0.00059268	-0.00006096	0.00007161
29	44,395,075	*ss86333994*		-0.00120791	-0.00077009	-0.00038065	0.00006199	-0.00002805	-0.00017409
29	44,416,282	*rs43709648*		-0.00203603	-0.00103453	-0.00093446	-0.00067339	-0.00001133	-0.00005219
29	44,546,564	*ss86288185*		0.00110217	0.00069894	0.00045516	0.00051185	-0.00000784	0.00005656
29	44,585,782	*ss86341172*		-0.00075142	-0.00059974	-0.00022848	-0.00066809	0.00004126	-0.00004148
29	44,628,137	*rs41600300*		-0.00005648	0.00034145	0.00005371	0.00013032	-0.00004817	-0.00003908
29	44,649,908	*ss86337530*		0.00005313	-0.00008978	0.00038621	-0.00022923	0.00001713	-0.00009300
29	44,740,917	*ss86334185*		0.00079430	0.00082804	0.00038222	-0.00025986	0.00002142	0.00002880
29	44,807,928	*ss117965824*		-0.00026913	0.00079928	-0.00045557	0.00002440	0.00000569	0.00002481
29	44,853,970	*rs42192429*		0.00005315	-0.00107690	0.00013023	-0.00052061	0.00003071	0.00010706
29	44,900,940	*rs43706228*		0.00089149	0.00166157	-0.00007800	0.00120207	-0.00000796	-0.00002885
29	44,969,518	*ss86337154*		-0.00025376	-0.00008685	0.00002682	-0.00024640	-0.00002614	-0.00002386
29	44,979,377	*ss86338214*		0.00021114	0.00036588	-0.00018187	0.00021199	0.00001482	0.00006627
29	44,999,264	*ss86310727*		0.00133191	0.00075573	0.00004545	0.00060918	0.00010639	0.00015623
29	45,023,665	*ss86338763*		-0.00019088	-0.00008685	0.00005425	-0.00023066	-0.00002614	-0.00001956
29	45,102,557	*ss86332221*		-0.00129469	-0.00064203	-0.00004862	-0.00111294	0.00001335	-0.00016787
29	45,129,099	*ss86335405*		0.00063776	0.00065799	0.00007693	0.00047129	0.00001030	0.00002804
29	45,187,114	*ss86295760*		-0.00047535	-0.00087672	-0.00015742	-0.00006285	-0.00002459	0.00001400
29	45,287,502	*ss86312485*		0.00053477	0.00045858	0.00002288	0.00071079	0.00001575	-0.00000847
29	45,326,585	*rs42198683*		0.00021305	0.00027334	-0.00028774	0.00099702	0.00001472	-0.00005160
29	45,367,095	*ss86322638*		0.00039329	0.00031861	0.00033922	-0.00017070	0.00003571	-0.00007839
29	45,458,280	*rs43706176*		-0.00020553	-0.00045743	0.00003098	-0.00005162	-0.00002502	0.00000069
29	45,482,143	*rs29025626*		-0.00084208	-0.00104550	-0.00002985	-0.00024322	-0.00010494	-0.00004481
29	45,530,264	*ss86341375*		0.00019820	0.00005755	0.00002545	-0.00001078	0.00003298	0.00003977

[1]UMD3.1 Coordinates. SNPs located within *CAST* and *CAPN1* are shaded in yellow.

[2]Commercialized SNPs are shaded in red.

[3]GBLUP allele substitution effect (α) ranks. Values less than 500 are shaded in yellow for the across-breed and green for the within-

[4]GBLUP of SNP allele substitution effects (α) estimated as a random effect.

[5]SNP allele substitution effects (β) estimated as fixed effects in a mixed linear model including the genomic relationship matrix.

[6]$-\log_{10}$(P)-value for the test of the null hypothesis of an allele substitution effect (β) of zero. Maximum values are shaded in red. Oth

[7]$-\log_{10}$(P)-value for the test of the null hypothesis of all haplotype substitution effects (H) being zero. Maximum values are shaded i

[8]Number of haplotypes and effective number of haplotypes in parentheses for the 13 (*CAST*) or 31 (*CAPN1*) SNP block centered on

[9]Number Percentage of phenotypic variation in WBSF explained by haplotypes centered on the identified SNP.

						ASE[5]		
BTA	POS[1]	SNP ID[2]	β-All Breeds	β-Angus	β-Hereford	β-Charolais	β-Limousin	β-Simmental
7	97,151,592	*rs41656985*	0.01377207	-0.01999276	0.03740770	0.01615795	-0.00029396	0.03399697
7	97,261,052	*rs41656987*	0.06407879	0.08585839	0.05622007	-0.00113931	0.15788787	0.11469922
7	97,372,656	*rs41570922*	-0.04721657	-0.10888501	0.02478650	-0.03871002	-0.15544713	0.02536908
7	97,393,157	*rs41615915*	-0.04605863	0.00265377	-0.10743993	0.00108729	-0.05573660	-0.07783973
7	97,430,691	*ss86295638*	0.05460805	-0.00518239	0.10506659	-0.02294942	-0.04461769	0.07698709
7	97,501,859	*ss86310801*	-0.03764247	-0.02495779	-0.04955234	-0.03468353	0.08999742	-0.09143018
7	97,529,872	*ss61472319*	0.02111581	-0.02877606	0.08140793	-0.01884309	0.10324757	-0.01642583
7	97,691,853	*ss86290016*	0.04112449	0.13325936	0.05822260	0.12703600	0.04074417	-0.09219867
7	97,772,240	*ss86312419*	-0.05640512	-0.07610179	-0.21729369	-0.02281948	0.30240522	0.00927325
7	97,861,341	*rs41593284*	0.05750871	0.01407221	0.11133231	-0.00899134	0.24638150	0.04204862
7	97,898,940	*rs41593281*	0.09632991	0.07850543	0.14152236	0.04174867	0.01595788	0.08909754
7	97,961,659	*ss117969141*	0.04610124	0.12701127	0.01169940	0.00594871	0.04862822	0.17698328
7	98,013,150	*ss117968909*	0.00300209	-0.01193793	-0.09714359	0.05201490	0.02690835	0.15605575
7	98,173,299	*ss86290989*	0.00767338	0.01261753	-0.06474281	0.07826779	0.01956097	0.02321594
7	98,194,828	*rs41658549*	-0.00401437	-0.04982220	0.09135510	-0.13132990	0.02229160	0.04134878
7	98,254,815	*rs41658542*	0.02188950	-0.01574779	0.20327112	-0.10260593	-0.07012338	0.03239528
7	98,375,640	*ss86329349*	0.09183851	0.12926199	0.20569660	0.00380099	0.11857929	-0.02275644
7	98,495,888	*rs43529872*	0.14912943	0.12638542	0.20438144	0.18318184	0.24750333	-0.03846871
7	98,498,047	*ss86321563*	0.14221431	0.15154723	0.20916753	0.15264208	0.18729389	-0.04713413
7	98,538,952	*rs41660170*	0.07895436	0.13526771	-0.00409153	0.08051120	0.07729644	0.11766511
7	98,566,391	*ss117962527*	-0.09262624	-0.16100475	-0.06040231	-0.16610346	-0.05483045	0.00153287
7	98,579,574	*rs41255587*	0.15449911	0.14576743	0.21255338	0.19065204	0.14454926	-0.02075111
7	98,635,208	*ss86300200*	-0.07152941	-0.03340736	-0.16969513	0.03199936	-0.10216313	-0.05727451
7	98,646,770	*rs41596489*	0.08027895	0.08287525	0.18982409	0.00170165	0.06672588	0.01508636
7	98,680,293	*ss86315737*	-0.05859165	-0.10106432	-0.15573983	0.02425142	0.06258840	-0.00192055
7	98,726,055	*rs29018251*	0.02359443	-0.12618996	0.07884128	0.05065498	0.00635118	0.01937443
7	98,757,265	*ss86303738*	0.02532671	-0.01615925	0.05567595	0.03238808	-0.01262411	0.11054776
7	98,820,742	*ss86324113*	0.07294052	0.08716323	0.17291781	-0.01161769	-0.05684945	0.10538010
7	98,887,313	*rs41568595*	-0.03694200	0.01652002	-0.02094997	-0.18933093	0.00660686	-0.02603140
7	98,907,404	*ss86291843*	-0.02347963	-0.00112256	-0.03939083	-0.02093560	-0.12977007	0.03256895
7	98,974,456	*ss86339659*	0.02520614	0.02355067	0.04765437	0.01524006	0.04806978	0.01211776
7	99,024,444	*ss117968912*	-0.00962625	-0.02314016	0.04887861	-0.06772105	0.06137752	-0.05045382
7	99,085,299	*rs41590403*	-0.03960914	-0.05700088	0.02546463	-0.07769533	-0.22934220	-0.01881188
7	99,130,598	*ss86333606*	-0.06239241	0.00956325	-0.14975990	0.02080513	0.03399904	0.11373474
7	99,178,748	*rs41568774*	-0.00494952	-0.00595571	0.04305756	-0.00422053	-0.06928228	-0.03732380
7	99,215,474	*ss86324013*	0.03866208	0.00323942	0.13672049	-0.00375089	0.01211855	-0.03134186
7	99,344,820	*rs41622872*	-0.04295510	0.02002393	-0.12011580	0.01951865	-0.13440981	-0.07138409
7	99,399,852	*ss117968895*	0.00566668	-0.05979255	0.05792219	-0.00246880	-0.06191476	-0.01295397
7	99,492,904	*ss86296856*	-0.06625218	-0.10116761	-0.17772993	-0.00151215	-0.01224771	0.01697012
7	99,635,841	*ss86339853*	0.00560736	0.00581627	0.05035461	-0.05124237	0.08750034	-0.07198733
7	99,687,614	*rs41657025*	0.01600630	-0.05598055	0.03944556	0.02693623	-0.00419388	0.07459634
7	99,845,133	*rs41624339*	0.05420017	0.01451857	0.08271281	0.04128013	-0.15385321	0.10367658
7	99,919,908	*ss86339607*	0.00320745	-0.00812169	-0.00021135	0.05011518	0.06678217	-0.03988769
7	100,015,468	*ss86310226*	-0.00884300	0.01313929	-0.00799969	-0.04322683	0.07312651	-0.04484400
29	42,406,544	*ss86319674*	0.00499755	0.13127994	-0.02539683	-0.05011073	0.37427564	-0.07250370
29	42,455,680	*ss117974470*	-0.01114205	-0.00287066	0.01625321	0.01350677	-0.08903101	-0.06311767
29	42,620,218	*rs42189770*	0.00437862	0.01893309	-0.02713130	0.07412626	-0.02035679	-0.02046697
29	42,651,294	*rs43706142*	-0.00912868	-0.07832798	0.04644727	-0.03432996	0.01516783	0.01150035
29	42,696,595	*rs42189112*	0.02244031	0.06801028	-0.03648918	0.10811249	-0.00408642	0.08595896
29	42,749,808	*rs29020063*	-0.00802797	0.03088923	0.01431881	-0.08214113	0.06422537	-0.11007124
29	42,842,353	*ss117974486*	-0.02723432	-0.05483318	0.06254769	0.01804422	-0.09758154	-0.20088805
29	42,897,144	*ss86322358*	0.01390338	-0.04068813	0.02886829	0.01772594	-0.01746038	0.10763037

29	43,006,000	*ss86339964*	0.00786039	-0.05214215	0.02769884	0.01772594	-0.01746038	0.09288296
29	43,043,207	*ss86341572*	0.01905446	-0.06102574	0.02423195	0.06732475	-0.08042507	0.10649011
29	43,108,445	*ss86337140*	-0.09515691	-0.18767192	-0.13426794	-0.05009274	-0.03198651	-0.10713556
29	43,129,250	*ss86337549*	-0.11213504	-0.36449310	-0.05938852	-0.01576700	-0.11245798	-0.07599159
29	43,189,370	*ss86319044*	-0.09177632	-0.35818174	-0.05938852	-0.00449407	-0.08165142	-0.07599159
29	43,224,753	*rs29013208*	0.07685161	0.23681481	-0.06367290	-0.03767127	0.07834478	0.13411425
29	43,269,744	*ss86282762*	0.06673067	0.22340633	-0.08516222	-0.04797645	0.06672672	0.13893634
29	43,304,256	*rs29024708*	-0.05533914	-0.18017752	0.05280083	-0.03602372	-0.02059741	-0.21167262
29	43,328,607	*ss86295061*	0.00409931	0.06677072	-0.08406764	0.05513809	0.03738891	-0.02303009
29	43,429,511	*ss86318094*	-0.08927452	-0.11940061	-0.19002689	0.02397019	-0.17416374	-0.07217357
29	43,466,342	*ss86310741*	-0.05527207	-0.18298300	-0.03897509	-0.08345126	0.21664736	-0.15334543
29	43,498,073	*ss86338800*	0.05956164	0.11996911	0.01582097	0.12870934	-0.13744882	0.12546311
29	43,611,640	*ss86298040*	0.07322512	0.03240417	0.07923634	0.11566400	-0.02602530	0.17796485
29	43,652,252	*ss86327310*	-0.05350543	-0.13120156	-0.04690503	-0.00705293	-0.05862478	-0.10243448
29	43,686,401	*ss86325323*	-0.01025002	-0.12481137	-0.05790675	0.07998412	0.02080392	0.07436958
29	43,709,769	*ss86290081*	-0.03614715	-0.09808855	-0.14603245	0.04284288	0.06642355	-0.04706384
29	43,747,765	*rs42190085*	-0.03252887	-0.03454110	0.00266994	-0.06688717	-0.03643018	-0.02716611
29	43,749,527	*ss86299346*	-0.01815565	0.04194873	0.00506304	-0.04813533	-0.07093084	-0.05943258
29	43,777,249	*ss86299989*	-0.00379751	-0.08865721	-0.00587409	0.03403038	0.00214447	-0.02166572
29	43,826,144	*rs42191289*	0.00538920	0.08261932	-0.04308125	-0.01792714	-0.10331144	0.16352930
29	43,839,783	*ss86336460*	0.02873918	0.09722623	-0.03889303	0.04674230	-0.05443953	0.17591056
29	43,914,923	*ss86335514*	0.14099316	0.12325906	0.11582581	0.17858418	0.10103476	0.27239461
29	44,000,728	*rs42191319*	-0.02134130	0.00009678	-0.04485603	0.05849863	-0.10881374	0.02442284
29	44,029,526	*rs42192038*	-0.04465678	-0.04790513	-0.01687600	-0.05861484	-0.06926916	-0.12931805
29	44,042,363	*rs42192064*	-0.09088154	-0.11220353	-0.06259312	-0.03927838	0.01761857	-0.31307293
29	44,049,244	*rs42192077*	0.02578055	-0.02692225	0.08254911	0.01055042	-0.00004142	-0.01123121
29	44,050,471	*ss86284058*	-0.06361724	0.02388787	-0.19851647	-0.07943526	-0.02527633	0.08868873
29	44,051,567	*rs42192079*	-0.02210319	0.01920409	-0.07340928	-0.01089515	-0.00185196	0.01290773
29	44,053,733	*rs42192083*	0.02086679	-0.02545535	0.07588543	0.00806255	0.00679728	-0.01483180
29	44,054,901	*rs42192084*	0.01512321	-0.02258374	0.04596259	0.04186951	0.02413509	-0.05674960
29	44,058,688	*rs42192090*	0.02968789	0.06906875	-0.02816224	0.11941891	-0.07838293	0.05512612
29	44,060,410	*rs42192096*	-0.02832327	-0.07343302	0.04272696	-0.09131420	0.06035150	-0.08307020
29	44,062,694	*rs42192100*	-0.02736293	-0.07396977	0.04226760	-0.08474445	0.05322737	-0.07665678
29	44,063,938	*rs17872078*	-0.18564305	-0.18455479	-0.20510618	-0.16345367	-0.03249970	-0.23021449
29	44,067,234	*rs17872093*	0.02496105	0.07343302	-0.05406733	0.09131420	-0.05322737	0.08515734
29	44,067,796	*rs17871984*	0.17226965	0.20960280	0.25854084	0.10460733	0.08033891	0.14060072
29	44,068,143	*rs17870631*	0.23869986	0.22119704	0.24379122	0.22023146	0.00547252	0.34767985
29	44,068,346	*rs17870628*	0.16042843	0.15402954	0.24841222	0.10918790	-0.05396674	0.19102806
29	44,068,445	*rs17870626*	0.22829997	0.21152447	0.23663609	0.21618169	-0.02950121	0.32679359
29	44,068,519	*rs17872010*	0.22980629	0.21678554	0.23000251	0.22023146	0.00547252	0.33560285
29	44,068,580	*rs17872004*	0.23576592	0.22119704	0.24158328	0.21914000	0.01402673	0.33693156
29	44,068,812	*rs17872003*	-0.23425299	-0.22039076	-0.24087221	-0.22570015	0.01719659	-0.33744060
29	44,069,063	*rs17812000*	-0.23938706	-0.27604109	-0.21680105	-0.22023146	-0.00547252	-0.33888089
29	44,069,177	*rs17872006*	-0.20865952	-0.22809167	-0.21525294	-0.17836251	0.05335006	-0.31640257
29	44,070,713	*rs42192103*	-0.23126101	-0.20077133	-0.25377584	-0.23262707	0.04616506	-0.33919363
29	44,070,881	*rs42192105*	-0.13150092	-0.09916322	-0.20618399	-0.03162694	0.02776421	-0.16146931
29	44,071,034	*rs42192107*	-0.14426575	-0.11047033	-0.21343493	-0.07328780	0.02776421	-0.16118552
29	44,071,135	*rs42192108*	-0.05688668	-0.02886829	-0.03653651	-0.04193928	-0.29972195	-0.02004771
29	44,071,593	*rs42192109*	-0.05027538	-0.10884482	0.01585778	-0.12844689	0.08759822	-0.09627713
29	44,085,642	*rs17871051*	0.14188557	0.30364766	0.10809754	0.11126940	0.13503153	0.20082779
29	44,085,769	*rs17871058*	0.14392345	0.15281348	0.06846588	0.18131448	0.28735643	0.19762528
29	44,087,205	*rs17872032*	0.18873706	0.25640780	0.03850535	0.20027467	0.15725079	0.30972904
29	44,087,356	*rs17872033*	-0.12775860	-0.27170879	-0.10219163	-0.10479101	-0.15498932	-0.15942987
29	44,087,629	*rs17872050*	0.14442059	0.16563130	0.06653446	0.18413259	0.30762227	0.17956041
29	44,087,858	*rs17872151*	0.14001255	-0.02272857	0.30154212	0.12323939	0.07713691	0.18930825

29	44,088,897	*rs17870847*	-0.14124160	-0.15463556	-0.05878994	-0.18885633	-0.30340823	-0.17524866
29	44,110,295	*rs42192119*	-0.01942996	0.01451903	-0.02155698	-0.06463021	-0.08669320	0.03046439
29	44,119,671	*rs42195143*	0.00783300	-0.02935938	0.07389328	-0.00669293	0.02115000	-0.02711373
29	44,122,272	*rs42194216*	-0.03172111	0.02819610	-0.08098997	-0.04973577	0.02964052	-0.00160258
29	44,125,747	*rs42194178*	-0.03356792	0.03659072	-0.09186858	-0.04942879	0.02964052	-0.00160258
29	44,130,887	*rs42194132*	0.02644605	-0.03659072	0.08421906	0.05072096	-0.02964052	-0.03311083
29	44,154,126	*rs42193349*	-0.05305730	0.22356813	-0.10323256	-0.05776275	0.04588778	-0.09831775
29	44,172,614	*ss117965984*	-0.03964292	0.00112919	-0.06918327	-0.04362937	0.04977295	-0.10354199
29	44,196,154	*ss86318958*	0.01564406	0.11621015	0.08928295	-0.04546044	0.03894493	-0.02006039
29	44,208,978	*rs29003633*	0.07439988	0.43869793	0.00575558	0.03243145	-0.04998931	-0.08817917
29	44,223,148	*ss86323783*	0.07582241	0.21702321	0.00803405	0.12606020	0.13368766	0.02592427
29	44,243,444	*ss86313099*	-0.00549653	0.11812362	-0.10476431	0.08059616	0.02143301	-0.03460116
29	44,325,408	*ss86292140*	-0.16398496	-0.20530041	-0.19003334	-0.12468567	-0.10603965	-0.24574655
29	44,372,611	*ss86329852*	-0.03713291	0.00488436	-0.08660821	-0.09708762	-0.17343672	0.13482641
29	44,395,075	*ss86333994*	-0.06754849	-0.10992804	-0.09616456	0.00711078	-0.03712152	-0.16178806
29	44,416,282	*rs43709648*	-0.13069556	-0.28351741	-0.18944356	-0.09438042	-0.01593376	-0.08385696
29	44,546,564	*ss86288185*	0.05494993	0.09262489	0.08928476	0.05312496	-0.01034263	0.05095507
29	44,585,782	*ss86341172*	-0.03866292	-0.06206035	-0.05039949	-0.08198585	0.06203462	-0.03992657
29	44,628,137	*rs41600300*	-0.01483796	0.20889018	0.15455551	0.06038629	-0.18452356	-0.14991919
29	44,649,908	*ss86337530*	0.00294146	-0.01196893	0.08771622	-0.02822089	0.02041584	-0.09897733
29	44,740,917	*ss86334185*	0.05012176	0.10695692	0.08835503	-0.03612618	0.10055830	0.03904043
29	44,807,928	*ss117965824*	-0.01428477	0.10722949	-0.08601306	0.00337124	0.00765424	0.02422262
29	44,853,970	*rs42192429*	0.00310823	-0.14208317	0.02747885	-0.07932534	0.06115144	0.11080152
29	44,900,940	*rs43706228*	0.04401726	0.16432737	-0.01507454	0.14127813	-0.01453902	-0.02478305
29	44,969,518	*ss86337154*	-0.08569177	-0.09080754	0.27706137	-0.16974889	-0.08763089	-0.07547633
29	44,979,377	*ss86338214*	0.00973100	0.03288093	-0.03428688	0.02287154	0.02023673	0.05906225
29	44,999,264	*ss86310727*	0.07636034	0.08409333	0.01064296	0.09008127	0.16345165	0.16162329
29	45,023,665	*ss86338763*	-0.06266468	-0.09080754	0.56904494	-0.14493435	-0.08763089	-0.06060103
29	45,102,557	*ss86332221*	-0.09128831	-0.10489015	-0.01759630	-0.20456570	0.01847027	-0.16251600
29	45,129,099	*ss86335405*	0.11751600	0.15237997	0.16724531	0.20802589	0.03794230	0.08782773
29	45,187,114	*ss86295760*	-0.02403866	-0.10408408	-0.03232491	-0.00787294	-0.03145318	0.01223186
29	45,287,502	*ss86312485*	0.09994421	0.09989672	0.05326782	0.21938837	0.26063282	-0.02542529
29	45,326,585	*rs42198683*	0.01064705	0.03273957	-0.05642473	0.10167291	0.02256656	-0.05231106
29	45,367,095	*ss86322638*	0.02307329	0.03872453	0.09838548	-0.01799483	0.09863873	-0.08139580
29	45,458,280	*rs43706176*	-0.02873231	-0.11397784	0.07484033	-0.01161712	-0.08531336	0.00126990
29	45,482,143	*rs29025626*	-0.05831395	-0.18623897	-0.00657759	-0.04365267	-0.32231770	-0.06272849
29	45,530,264	*ss86341375*	0.02131837	0.05111941	0.00589401	-0.00306374	0.15679441	0.20788969

[1]UMD3.1 Coordinates. SNPs located within *CAST* and *CAPN1* are shaded in yellow.

[2]Commercialized SNPs are shaded in red.

[3]GBLUP allele substitution effect (α) ranks. Values less than 500 are shaded in yellow for the across-breed and green for the withi

[4]GBLUP of SNP allele substitution effects (α) estimated as a random effect.

[5]SNP allele substitution effects (β) estimated as fixed effects in a mixed linear model including the genomic relationship matrix.

[6]-$\log_{10}$(P)-value for the test of the null hypothesis of an allele substitution effect (β) of zero. Maximum values are shaded in red. Ot

[7]-$\log_{10}$(P)-value for the test of the null hypothesis of all haplotype substitution effects (H) being zero. Maximum values are shaded

[8]Number of haplotypes and effective number of haplotypes in parentheses for the 13 (*CAST*) or 31 (*CAPN1*) SNP block centered o

[9]Number Percentage of phenotypic variation in WBSF explained by haplotypes centered on the identified SNP.

						-log$_{10}$(P)ASE[6]		
BTA	POS[1]	SNP ID[2]	β-All Breeds	β-Angus	β-Hereford	β-Charolais	β-Limousin	β-Simmental
7	97,151,592	*rs41656985*	0.25920819	0.19065791	0.38791104	0.17268288	0.00129203	0.24547389
7	97,261,052	*rs41656987*	1.76394501	0.96309267	0.46932913	0.00960706	1.15663403	1.13658381
7	97,372,656	*rs41570922*	0.51757650	0.70940830	0.06022060	0.24466141	0.62472795	0.10403711
7	97,393,157	*rs41615915*	1.21374534	0.02346959	1.51546840	0.00867879	0.28640193	0.65871607
7	97,430,691	*ss86295638*	1.19548289	0.02323031	1.47921826	0.16870415	0.19703810	0.49965269
7	97,501,859	*ss86310801*	0.57707351	0.12657802	0.34928548	0.26680432	0.48566729	0.50598038
7	97,529,872	*ss61472319*	0.43312604	0.30306271	1.11617585	0.19968551	0.64220544	0.10489070
7	97,691,853	*ss86290016*	0.28588801	0.40039921	0.14156066	0.60508426	0.08498000	0.37342394
7	97,772,240	*ss86312419*	0.86395033	0.75292586	1.67261725	0.15800490	0.91674896	0.03562833
7	97,861,341	*rs41593284*	1.53245331	0.12732425	1.13126148	0.07533843	2.64951708	0.29967328
7	97,898,940	*rs41593281*	3.42270182	0.71645917	2.89390082	0.29177371	0.06921540	0.71900816
7	97,961,659	*ss117969141*	0.35277075	0.54610435	0.03956972	0.02062259	0.07559241	0.54397980
7	98,013,150	*ss117968909*	0.04524314	0.10712272	1.36976476	0.69474730	0.12949977	1.90813017
7	98,173,299	*ss86290989*	0.10943140	0.10718998	0.60809591	0.91744683	0.09275246	0.13070592
7	98,194,828	*rs41658549*	0.05932558	0.62843426	1.13325108	2.56758808	0.09789700	0.31044044
7	98,254,815	*rs41658542*	0.39532965	0.15482480	3.06228709	1.65770810	0.40341524	0.21863565
7	98,375,640	*ss86329349*	1.99903332	1.38818084	2.35512143	0.02362894	0.35954871	0.09411136
7	98,495,888	*rs43529872*	7.74766418	1.99768902	5.22892170	3.62427416	1.47999444	0.24953504
7	98,498,047	*ss86321563*	7.42639931	2.56544376	5.48906355	3.03632723	1.00081075	0.34592414
7	98,538,952	*rs41660170*	1.19616937	1.31606193	0.01343470	0.72869343	0.20372510	0.51234229
7	98,566,391	*ss117962527*	2.13133465	1.95083162	0.27369699	2.44272959	0.25132133	0.00778098
7	98,579,574	*rs41255587*	8.94616131	2.54505254	5.63316775	4.25399597	1.02795263	0.12970451
7	98,635,208	*ss86300200*	2.78390969	0.39024136	3.88488884	0.38083683	0.68919867	0.49697830
7	98,646,770	*rs41596489*	3.09302733	1.42263877	4.15919781	0.01538183	0.31858437	0.09451937
7	98,680,293	*ss86315737*	1.85792616	1.73175280	3.13848683	0.24889004	0.33750113	0.01134466
7	98,726,055	*rs29018251*	0.42728013	1.90332787	0.82779401	0.65329731	0.02111718	0.12131636
7	98,757,265	*ss86303738*	0.38498763	0.14407068	0.26033788	0.27396792	0.05509740	0.90591284
7	98,820,742	*ss86324113*	2.38541310	1.44419614	2.83381698	0.10584866	0.24004092	1.05287378
7	98,887,313	*rs41568595*	0.53301860	0.08132593	0.13823755	1.53243070	0.02459171	0.11513550
7	98,907,404	*ss86291843*	0.49381227	0.00947962	0.44931494	0.21138458	0.82054150	0.20763072
7	98,974,456	*ss86339659*	0.54215062	0.26713043	0.57315687	0.14318927	0.19621006	0.06602532
7	99,024,444	*ss117968912*	0.16733868	0.22504722	0.58555919	1.02559764	0.31898397	0.38434202
7	99,085,299	*rs41590403*	0.69481474	0.39318251	0.18795130	0.75050635	1.24660458	0.10624050
7	99,130,598	*ss86333606*	1.45570588	0.06251611	3.10294183	0.10574727	0.10273524	0.70938049
7	99,178,748	*rs41568774*	0.06868082	0.04898987	0.34108982	0.03196463	0.33551761	0.26193372
7	99,215,474	*ss86324013*	0.79647808	0.02351372	1.69652140	0.02952516	0.05206018	0.18142548
7	99,344,820	*rs41622872*	1.14352553	0.20570883	1.95773851	0.19025349	1.01497461	0.59501667
7	99,399,852	*ss117968895*	0.05287460	0.44925767	0.36214999	0.01233920	0.11948527	0.04633037
7	99,492,904	*ss86296856*	1.15969824	1.09708746	1.54697553	0.00766042	0.04497447	0.06521665
7	99,635,841	*ss86339853*	0.08261138	0.04523761	0.50695580	0.60607729	0.47188976	0.57737955
7	99,687,614	*rs41657025*	0.26637232	0.67054698	0.28528814	0.29544248	0.01572183	0.65781175
7	99,845,133	*rs41624339*	1.34660053	0.12133397	1.11618627	0.41055563	0.56501728	0.71205727
7	99,919,908	*ss86339607*	0.04573644	0.07034137	0.00151539	0.49076674	0.36871254	0.27101227
7	100,015,468	*ss86310226*	0.13573436	0.12458893	0.04893090	0.49885840	0.39851707	0.32578504
29	42,406,544	*ss86319674*	0.04986806	1.37866264	0.16134861	0.32115619	0.87009257	0.39403172
29	42,455,680	*ss117974470*	0.19574411	0.02641378	0.14141656	0.11127875	0.59456400	0.55434182
29	42,620,218	*rs42189770*	0.05722611	0.14305650	0.22931731	0.75240467	0.07529309	0.11527340
29	42,651,294	*rs43706142*	0.13527357	1.19216972	0.32601885	0.36725161	0.06524151	0.06544274
29	42,696,595	*rs42189112*	0.44441091	0.88270802	0.37685810	1.74463604	0.01829748	0.76562942
29	42,749,808	*rs29020063*	0.12666589	0.35244490	0.11091297	1.11550797	0.37213472	1.16115545
29	42,842,353	*ss117974486*	0.55536657	0.69280424	0.64651365	0.16318906	0.62728700	3.18305827
29	42,897,144	*ss86322358*	0.18345331	0.39458459	0.13964490	0.14498396	0.06992430	0.80845199

29	43,006,000	*ss86339964*	0.09586061	0.53898948	0.13318280	0.14498396	0.06992430	0.64216062
29	43,043,207	*ss86341572*	0.29218063	0.76407239	0.12313821	0.82608844	0.42613324	0.96006708
29	43,108,445	*ss86337140*	2.45598566	2.31486819	1.44440671	0.37952271	0.13871960	0.77016154
29	43,129,250	*ss86337549*	1.61915754	4.63029649	0.09175169	0.06199632	0.59514087	0.35844100
29	43,189,370	*ss86319044*	1.25955168	4.56366938	0.09175169	0.01982073	0.38561856	0.35844100
29	43,224,753	*rs29013208*	1.12052175	3.01526390	0.12262529	0.22241437	0.37189194	0.87811532
29	43,269,744	*ss86282762*	0.76185346	2.53505836	0.15756175	0.28646199	0.27662167	0.48219951
29	43,304,256	*rs29024708*	1.06275615	2.25837870	0.36526377	0.27590072	0.08300408	2.41431365
29	43,328,607	*ss86295061*	0.05426188	0.94535620	0.72983337	0.54636948	0.16252304	0.14634925
29	43,429,511	*ss86318094*	3.30593304	2.47535623	3.68132176	0.23311529	0.77433371	0.60737632
29	43,466,342	*ss86310741*	0.96296598	2.56839941	0.28684653	0.70252500	1.08226275	0.86478838
29	43,498,073	*ss86338800*	1.52028312	1.76670225	0.13365834	1.83411013	0.61664702	1.03469542
29	43,611,640	*ss86298040*	2.58028835	0.36374051	1.04977109	2.06890532	0.12475821	2.28082929
29	43,652,252	*ss86327310*	1.68525775	2.97699466	0.55688163	0.06156001	0.32155822	1.12682579
29	43,686,401	*ss86325323*	0.12890225	1.76992464	0.55000156	0.70435872	0.04177780	0.43177499
29	43,709,769	*ss86290081*	0.73588744	1.88286213	1.40490730	0.48685638	0.33902519	0.31447126
29	43,747,765	*rs42190085*	0.61498554	0.29925040	0.01867855	0.69135855	0.15545894	0.15423447
29	43,749,527	*ss86299346*	0.23117133	0.28797421	0.03062952	0.39580652	0.14410590	0.32099723
29	43,777,249	*ss86299989*	0.05204709	1.23855542	0.04279939	0.30596175	0.00862948	0.12086964
29	43,826,144	*rs42191289*	0.08355832	1.07275064	0.46344422	0.17992963	0.60679991	2.10421872
29	43,839,783	*ss86336460*	0.65687172	1.57750548	0.42813638	0.60240929	0.27736123	2.57848708
29	43,914,923	*ss86335514*	5.41309793	1.21043891	1.29638005	3.23934367	0.57977131	3.48219782
29	44,000,728	*rs42191319*	0.28733855	0.00041707	0.43862251	0.33148269	0.35713837	0.11318397
29	44,029,526	*rs42192038*	1.20751437	0.61860399	0.15163110	0.76352207	0.29883743	1.50891107
29	44,042,363	*rs42192064*	3.78983596	2.11305517	0.81294077	0.43141451	0.07967046	6.51065616
29	44,049,244	*rs42192077*	0.53544650	0.28049136	1.11635461	0.09636135	0.00017937	0.06853338
29	44,050,471	*ss86284058*	1.31634950	0.18165119	2.73155940	0.77897574	0.08559228	0.55261272
29	44,051,567	*rs42192079*	0.43791985	0.18793165	0.94534508	0.09995925	0.00801219	0.08018721
29	44,053,733	*rs42192083*	0.40600605	0.26290107	0.98615509	0.07197118	0.03036354	0.09347804
29	44,054,901	*rs42192084*	0.23271677	0.19155845	0.43966180	0.42005085	0.10259460	0.34485314
29	44,058,688	*rs42192090*	0.63374532	1.03088313	0.26325766	2.16293094	0.44993328	0.45997855
29	44,060,410	*rs42192096*	0.58111065	1.09484368	0.44353513	1.43528346	0.29247259	0.79125781
29	44,062,694	*rs42192100*	0.55597264	1.10606032	0.43772073	1.27617242	0.24952277	0.72058476
29	44,063,938	*rs17872078*	11.06385692	4.73157664	3.95490039	3.60275558	0.12298021	3.56044339
29	44,067,234	*rs17872093*	0.49119246	1.09484368	0.61056779	1.43528346	0.24952277	0.82128250
29	44,067,796	*rs17871984*	9.12386593	4.17029373	6.17311920	1.38841165	0.33534977	1.65236722
29	44,068,143	*rs17870631*	15.02478285	6.53020157	4.79267429	5.02153497	0.01743977	5.61780734
29	44,068,346	*rs17870628*	8.02478258	3.72104979	5.03083483	1.69430079	0.23008326	2.29019068
29	44,068,445	*rs17870626*	14.02591772	5.99882130	4.56681868	4.87245328	0.10332268	5.32147410
29	44,068,519	*rs17872010*	13.92040678	6.23558901	4.32611748	5.02153497	0.01743977	5.22609254
29	44,068,580	*rs17872004*	14.69756940	6.53020157	4.70900007	4.95672805	0.04699462	5.33363070
29	44,068,812	*rs17872003*	14.52861819	6.54890094	4.68559191	5.23726316	0.05731284	5.26903465
29	44,069,063	*rs17812000*	15.01318351	9.70325035	3.86955288	5.02153497	0.01743977	5.34387541
29	44,069,177	*rs17872006*	12.16297032	6.95858004	3.95278603	3.63333825	0.20827835	4.93570623
29	44,070,713	*rs42192103*	15.25158225	5.57498348	5.51973138	5.66567676	0.18467504	5.99674646
29	44,070,881	*rs42192105*	3.44303003	1.04085226	3.19416028	0.16828075	0.05476708	0.95464665
29	44,071,034	*rs42192107*	3.60877221	1.14058147	3.31494388	0.42816961	0.05476708	0.59604337
29	44,071,135	*rs42192108*	0.84761227	0.11400826	0.24937838	0.31296813	1.21868023	0.05172524
29	44,071,593	*rs42192109*	1.34891868	2.04739447	0.13589882	2.48998697	0.46197675	0.93864158
29	44,085,642	*rs17871051*	5.52314725	6.47497947	0.90205454	1.69543379	0.89215774	2.61559871
29	44,085,769	*rs17871058*	7.11609711	3.39328007	0.60316732	4.37345894	2.84572845	2.88941271
29	44,087,205	*rs17872032*	4.07412817	2.69647902	0.11119448	2.27449768	0.61567777	2.27417512
29	44,087,356	*rs17872033*	4.80268257	5.73921171	0.86225702	1.56598606	1.12117208	1.91960350
29	44,087,629	*rs17872050*	7.27411721	3.95801125	0.58442547	4.49008758	3.23458930	2.59790176
29	44,087,858	*rs17872151*	1.91592723	0.05811805	1.11957708	0.90851090	0.28340516	0.74334865

29	44,088,897	*rs17870847*	6.98857664	3.53198021	0.49906676	4.69114559	3.16712717	2.42102017
29	44,110,295	*rs42192119*	0.24763846	0.11219104	0.09233846	0.61056832	0.36911136	0.14787609
29	44,119,671	*rs42195143*	0.13149919	0.33349792	0.99637011	0.05862776	0.10126358	0.17576138
29	44,122,272	*rs42194216*	0.44091580	0.18173824	0.76346807	0.27321828	0.10568910	0.00595621
29	44,125,747	*rs42194178*	0.47074672	0.24535880	0.89696110	0.27137387	0.10568910	0.00595621
29	44,130,887	*rs42194132*	0.34790036	0.24535880	0.80201702	0.27822368	0.10568910	0.14103651
29	44,154,126	*rs42193349*	0.73134565	1.60354632	1.02285973	0.22531657	0.16005057	0.43020523
29	44,172,614	*ss117965984*	0.89921288	0.00903521	0.79790059	0.48422659	0.20438421	0.83691045
29	44,196,154	*ss86318958*	0.17826692	0.83275480	0.79139179	0.31733155	0.09584821	0.09352201
29	44,208,978	*rs29003633*	0.72332983	4.82095682	0.01810898	0.13778251	0.05217681	0.29638830
29	44,223,148	*ss86323783*	1.87225920	3.93233393	0.04523253	1.83618667	0.67743646	0.14198146
29	44,243,444	*ss86313099*	0.08257010	1.92540086	1.69616348	0.95541644	0.08870579	0.24639965
29	44,325,408	*ss86292140*	5.67467249	5.33541800	1.08931228	1.55484928	0.44966064	2.51916267
29	44,372,611	*ss86329852*	0.53162591	0.02803790	0.48418639	1.23731403	0.80255075	0.90485283
29	44,395,075	*ss86333994*	2.06219114	1.66817727	1.16265211	0.06130758	0.18184149	2.06865498
29	44,416,282	*rs43709648*	5.68390548	4.77448860	4.22466506	1.33513815	0.06867912	0.52090920
29	44,546,564	*ss86288185*	1.62442034	1.35229526	1.25855827	0.71522273	0.04494773	0.39605969
29	44,585,782	*ss86341172*	0.93033545	0.89359393	0.51199420	1.19185100	0.31208265	0.27979050
29	44,628,137	*rs41600300*	0.10219117	1.45782122	0.41298898	0.31552270	0.70959496	0.63515653
29	44,649,908	*ss86337530*	0.04163328	0.09909412	1.09696773	0.27955691	0.09828177	0.87398116
29	44,740,917	*ss86334185*	1.17528419	1.72921586	1.09441063	0.35244420	0.28055154	0.22148194
29	44,807,928	*ss117965824*	0.24486922	1.68461918	1.22415017	0.02552680	0.03251072	0.15375166
29	44,853,970	*rs42192429*	0.04285861	2.70896169	0.24443504	0.96548290	0.25802019	1.05292526
29	44,900,940	*rs43706228*	1.16255448	4.47829875	0.12815280	2.96298435	0.05421330	0.16986484
29	44,969,518	*ss86337154*	0.75559117	0.31551240	0.38456438	0.97456211	0.29136167	0.29457028
29	44,979,377	*ss86338214*	0.16929778	0.41243332	0.34406199	0.23475380	0.09033549	0.48360841
29	44,999,264	*ss86310727*	2.47136644	1.33314498	0.07862254	1.19352282	1.15758380	1.89272860
29	45,023,665	*ss86338763*	0.50154984	0.31551240	1.02362911	0.82814950	0.29136167	0.22855959
29	45,102,557	*ss86332221*	2.80208621	1.39414911	0.10735358	3.81708940	0.08121695	2.01548437
29	45,129,099	*ss86335405*	1.92518407	1.91040532	0.55154806	1.87871179	0.10445101	0.35823047
29	45,187,114	*ss86295760*	0.48656271	1.77102946	0.30303839	0.06520406	0.15318487	0.07669521
29	45,287,502	*ss86312485*	1.47288059	1.04081145	0.13075897	2.75606820	0.42122552	0.08683296
29	45,326,585	*rs42198683*	0.17928117	0.34149476	0.64650443	1.93008045	0.09554951	0.37980161
29	45,367,095	*ss86322638*	0.41840731	0.41923223	1.08477513	0.17936027	0.38174958	0.67379779
29	45,458,280	*rs43706176*	0.31849023	1.14656944	0.18898358	0.07227556	0.27896857	0.00510871
29	45,482,143	*rs29025626*	1.37928192	3.32818270	0.04864044	0.38112107	1.95322650	0.38976296
29	45,530,264	*ss86341375*	0.25925427	0.17470233	0.04224125	0.01600602	0.49028244	0.80895685

[1]UMD3.1 Coordinates. SNPs located within *CAST* and *CAPN1* are shaded in yellow.

[2]Commercialized SNPs are shaded in red.

[3]GBLUP allele substitution effect (α) ranks. Values less than 500 are shaded in yellow for the across-breed and green for the with

[4]GBLUP of SNP allele substitution effects (α) estimated as a random effect.

[5]SNP allele substitution effects (β) estimated as fixed effects in a mixed linear model including the genomic relationship matrix.

[6]$-\log_{10}$(P)-value for the test of the null hypothesis of an allele substitution effect (β) of zero. Maximum values are shaded in red. (

[7]$-\log_{10}$(P)-value for the test of the null hypothesis of all haplotype substitution effects (H) being zero. Maximum values are shade

[8]Number of haplotypes and effective number of haplotypes in parentheses for the 13 (*CAST*) or 31 (*CAPN1*) SNP block centered

[9]Number Percentage of phenotypic variation in WBSF explained by haplotypes centered on the identified SNP.

						-log$_{10}$(P)HSE[7]		
BTA	POS[1]	SNP ID[2]	H-All Breeds	H-Angus	H-Hereford	H-Charolais	H-Limousin	H-Simmental
7	97,151,592	*rs41656985*						
7	97,261,052	*rs41656987*						
7	97,372,656	*rs41570922*						
7	97,393,157	*rs41615915*						
7	97,430,691	*ss86295638*	0.26508076	0.22716139	0.35490286	0.11939695	1.17950014	0.05611460
7	97,501,859	*ss86310801*	0.81800928	0.07156966	0.33609847	0.06345397	2.15235804	0.09168065
7	97,529,872	*ss61472319*	1.98049919	0.14371942	1.39259947	0.12678032	1.29152534	0.15902671
7	97,691,853	*ss86290016*	1.38106720	0.03262946	1.44345770	0.06604644	1.08465456	0.00273854
7	97,772,240	*ss86312419*	1.11696273	0.03704186	0.59346134	0.01414687	1.48842612	0.51559835
7	97,861,341	*rs41593284*	1.07333880	0.18884830	1.54612811	1.28697105	1.94622343	0.70741950
7	97,898,940	*rs41593281*	1.20710768	0.21395773	0.35285888	0.56983555	0.84014096	0.97571611
7	97,961,659	*ss117969141*	1.56513141	0.58260066	1.18631024	0.80927320	1.45814808	0.69798479
7	98,013,150	*ss117968909*	1.94109221	0.42866082	1.70014361	1.07709074	1.53017736	0.54579568
7	98,173,299	*ss86290989*	5.19485373	0.21666189	3.08995548	0.57537080	0.40169634	1.26846310
7	98,194,828	*rs41658549*	7.19312845	1.45022845	3.28674164	0.88346307	0.94186821	1.09107705
7	98,254,815	*rs41658542*	7.30762392	1.20874235	3.86521461	0.64348316	1.16137417	1.44864297
7	98,375,640	*ss86329349*	5.20735013	0.69984802	3.62626557	0.21261409	0.99651092	1.49969468
7	98,495,888	*rs43529872*	8.78946851	2.75028009	3.88742490	3.77219492	1.18671624	0.67066205
7	98,498,047	*ss86321563*	5.26395768	0.95003489	3.26823256	1.24059094	0.59144138	0.50885778
7	98,538,952	*rs41660170*	5.75844597	1.24611419	4.18746267	4.02764991	0.34395397	1.25539145
7	98,566,391	*ss117962527*	5.29372661	0.80510588	4.56286232	2.37126445	0.89744762	0.24031325
7	98,579,574	*rs41255587*	3.98897683	0.52039442	3.44498563	2.02857191	0.63931022	0.40299644
7	98,635,208	*ss86300200*	2.32088832	0.36484788	3.13453957	1.47339982	1.97169891	0.04134551
7	98,646,770	*rs41596489*	3.35672116	0.12551053	2.35292792	0.83513958	0.61692184	0.74500295
7	98,680,293	*ss86315737*	1.46829182	0.45527887	1.11997405	0.72275624	1.52410372	0.77676452
7	98,726,055	*rs29018251*	2.68450826	0.00446483	2.59819051	1.64736930	0.04882888	1.21792070
7	98,757,265	*ss86303738*	0.25145766	0.01037767	1.07605607	0.11879893	0.05675627	0.52793539
7	98,820,742	*ss86324113*	1.27277626	0.00316008	0.87855733	0.82996525	0.16650701	0.07170139
7	98,887,313	*rs41568595*	1.04874555	0.24055696	1.92106638	2.27907223	0.25832342	0.34138781
7	98,907,404	*ss86291843*	0.12659421	0.04140937	0.47312626	1.08738269	0.30143055	0.17518428
7	98,974,456	*ss86339659*	0.35618278	0.11996119	0.43000780	3.20102208	1.59395272	0.16889318
7	99,024,444	*ss117968912*	1.38169398	0.46449100	2.42800812	3.82249522	0.33963017	0.03566803
7	99,085,299	*rs41590403*	1.41246704	0.22315877	2.07804148	1.22225322	0.25108351	0.12469519
7	99,130,598	*ss86333606*	0.46025697	0.00984278	1.09712190	0.87025315	0.99486135	0.02418621
7	99,178,748	*rs41568774*	0.27207244	0.07530685	0.40169694	1.03480593	0.17213830	0.08893838
7	99,215,474	*ss86324013*	1.54040285	0.23635459	1.69528036	0.45088169	0.01317582	0.20992227
7	99,344,820	*rs41622872*	0.77418023	1.10702407	0.64002365	0.03192407	0.45514191	0.44536115
7	99,399,852	*ss117968895*	0.02699322	0.01194669	0.05783008	0.01770490	0.62311139	0.00584030
7	99,492,904	*ss86296856*	0.67099476	0.03420171	0.23136372	0.31714738	0.56405439	0.21482810
7	99,635,841	*ss86339853*	0.26382175	0.06423947	0.07232531	0.24511858	1.09094161	0.22922627
7	99,687,614	*rs41657025*						
7	99,845,133	*rs41624339*						
7	99,919,908	*ss86339607*						
7	100,015,468	*ss86310226*						
29	42,406,544	*ss86319674*						
29	42,455,680	*ss117974470*						
29	42,620,218	*rs42189770*						
29	42,651,294	*rs43706142*						
29	42,696,595	*rs42189112*	0.30906697	0.88607575	0.02123152	0.39494132	0.15097430	0.23355190
29	42,749,808	*rs29020063*	0.60218769	0.98996223	0.03249730	0.59290246	0.34019608	1.19943101
29	42,842,353	*ss117974486*	1.13550193	0.65699706	0.47372237	0.02181479	0.08405313	1.12277303
29	42,897,144	*ss86322358*	0.71512224	2.11448646	0.53312529	0.14369412	0.21209756	0.88613922

29	43,006,000	*ss86339964*	0.73271265	2.41188485	0.33960364	0.08529153	0.77321440	1.56473566
29	43,043,207	*ss86341572*	0.82246094	2.32985985	0.11124371	0.09751926	0.15356810	1.43976778
29	43,108,445	*ss86337140*	0.81896949	2.33109058	0.00285613	0.00222270	0.18021498	1.69495979
29	43,129,250	*ss86337549*	1.63994035	4.20422145	0.06100995	0.16876456	0.03220266	0.93963329
29	43,189,370	*ss86319044*	1.18301713	1.19782548	0.86122390	0.01091282	0.23608231	0.49359785
29	43,224,753	*rs29013208*	2.37813436	3.18593611	1.67880213	0.01106032	0.11811475	0.61501851
29	43,269,744	*ss86282762*	3.86475938	3.82149385	2.26416192	0.14418999	0.55321311	0.92959597
29	43,304,256	*rs29024708*	1.24577534	1.55404902	0.83388990	0.16568222	0.13932080	1.35982228
29	43,328,607	*ss86295061*	1.62487690	2.40837626	0.42667436	1.64421339	0.02121940	1.33710261
29	43,429,511	*ss86318094*	2.12572529	3.80842344	1.28881993	0.95480642	0.02553625	1.44611500
29	43,466,342	*ss86310741*	0.71555522	1.12116750	0.88703677	0.33284474	0.00270686	0.56692846
29	43,498,073	*ss86338800*	1.45812150	2.95862003	0.90383764	0.85347409	0.41198322	0.31775444
29	43,611,640	*ss86298040*	1.71738830	1.49905151	0.65021301	0.56840134	0.58927359	0.81617442
29	43,652,252	*ss86327310*	1.85247447	2.72293599	0.38704847	1.21836700	0.46366774	1.06027271
29	43,686,401	*ss86325323*	2.85471722	5.50057900	0.89673711	1.10064931	0.00287942	2.04317052
29	43,709,769	*ss86290081*	2.37660557	7.30961397	0.69453568	0.90919636	0.76372755	2.56351112
29	43,747,765	*rs42190085*	1.57659695	2.45959885	0.82975654	0.08757620	0.69431647	2.26028050
29	43,749,527	*ss86299346*	1.17290269	1.12334737	0.87827269	0.58774448	0.69851826	1.38948401
29	43,777,249	*ss86299989*	3.02787309	1.34862602	1.70160346	0.33254679	1.47845730	1.58800258
29	43,826,144	*rs42191289*	5.32853039	1.18426692	3.31864667	0.80798556	0.11909686	1.37750458
29	43,839,783	*ss86336460*	0.34757971	1.24877665	0.88353443	0.25582816	0.10286128	0.42109271
29	43,914,923	*ss86335514*	0.41546339	0.90700286	0.13421452	0.59111298	0.14605836	0.88599779
29	44,000,728	*rs42191319*	0.82185269	0.04755089	0.49823042	0.84353654	0.13510732	0.83505751
29	44,029,526	*rs42192038*	0.50880964	0.31129890	0.27972788	0.86600833	0.26925594	0.44131506
29	44,042,363	*rs42192064*	0.13658017	0.11580871	0.72905363	0.71583709	0.01905937	0.08194063
29	44,049,244	*rs42192077*	1.35767164	1.22634702	0.85886097	1.46652883	0.02509769	0.70198908
29	44,050,471	*ss86284058*	1.61646995	1.67349815	0.67683014	0.48665107	0.28414395	1.97858819
29	44,051,567	*rs42192079*	3.30712727	1.42085559	2.44795993	0.82580793	0.00975328	2.86690989
29	44,053,733	*rs42192083*	3.39158339	2.38296426	1.30292318	0.94212815	0.21402334	3.00111352
29	44,054,901	*rs42192084*	11.49369842	3.55621855	5.21648679	3.10996361	0.23084442	1.99917782
29	44,058,688	*rs42192090*	7.88594584	1.80903613	5.70470916	3.31341794	0.09513478	2.83102329
29	44,060,410	*rs42192096*	0.12986183	1.46336669	2.16083744	1.23070016	0.45062150	0.15558495
29	44,062,694	*rs42192100*	12.27238874	2.41838262	6.74301670	1.67412867	0.48273454	1.60991865
29	44,063,938	*rs17872078*	12.23589788	2.29047402	5.87511925	2.49050918	0.10200335	2.19095222
29	44,067,234	*rs17872093*	16.88269453	6.57015459	5.61324508	3.26121021	0.26036723	4.51981527
29	44,067,796	*rs17871984*	18.42738682	5.84412338	5.71691132	4.08482900	0.27637483	4.20909177
29	44,068,143	*rs17870631*	18.39968289	6.37524252	5.45793276	4.05568887	0.23002304	4.03478613
29	44,068,346	*rs17870628*	12.38241270	5.04806672	2.70913095	4.10912997	0.24068611	2.93239801
29	44,068,445	*rs17870626*	13.82885695	9.34358563	3.53168518	4.15858213	0.32596470	3.28779936
29	44,068,519	*rs17872010*	15.25910115	9.89889309	5.93905737	4.63045660	1.24106440	3.82501971
29	44,068,580	*rs17872004*	6.47969623	5.68172394	3.81874178	0.49614568	0.25001902	1.82874448
29	44,068,812	*rs17872003*	2.05963582	2.46398596	1.24572801	1.47993935	0.15068095	0.56014166
29	44,069,063	*rs17812000*	9.77853894	7.47435434	2.39643816	2.79419971	0.12049838	3.66924718
29	44,069,177	*rs17872006*	13.46353251	7.96050207	3.86770851	4.29375576	0.88025979	3.88562500
29	44,070,713	*rs42192103*	11.55151331	6.44427023	4.27234495	4.12354576	0.95747805	2.99347063
29	44,070,881	*rs42192105*	13.20467660	6.97082267	4.74847929	5.85379933	1.34839067	2.63128615
29	44,071,034	*rs42192107*	12.98510976	6.43286369	4.17187350	5.64602414	0.28760212	2.92776017
29	44,071,135	*rs42192108*	2.37657109	2.85669709	1.57339654	0.99186327	0.28553372	0.51775051
29	44,071,593	*rs42192109*	2.43465244	2.35156168	1.72365835	1.06696035	0.56643274	0.11445303
29	44,085,642	*rs17871051*	2.22122719	3.74051691	0.20554006	3.10807112	0.97276859	2.13239684
29	44,085,769	*rs17871058*	6.61208861	8.38064456	3.00878845	3.07580730	1.26456466	2.23802562
29	44,087,205	*rs17872032*	6.40228903	7.70690103	1.37392140	1.67479791	1.32025609	2.25287170
29	44,087,356	*rs17872033*	2.71654734	2.41023275	0.82639683	0.30604784	1.49210014	0.98590337
29	44,087,629	*rs17872050*	2.98168563	1.19410286	1.05055383	1.77522158	0.83669683	1.28942592
29	44,087,858	*rs17872151*	1.93894476	1.14413413	1.96526756	0.39565199	0.29177155	0.62766408

29	44,088,897	*rs17870847*	0.16133199	0.79403792	0.21583410	0.49005619	0.02166887	0.00111337
29	44,110,295	*rs42192119*	1.60735942	0.87925948	0.21436998	1.12419681	0.03163904	1.06791215
29	44,119,671	*rs42195143*	1.22792288	0.54340136	0.97473257	0.20362366	0.10037664	0.29155327
29	44,122,272	*rs42194216*	2.51318370	0.47540070	1.04175576	2.70682242	0.14208366	1.16366448
29	44,125,747	*rs42194178*	2.23965847	0.35775641	0.18928124	2.96180470	0.07248796	1.32894979
29	44,130,887	*rs42194132*	1.81890816	0.46168104	0.39822265	1.67786057	0.29121497	1.17182571
29	44,154,126	*rs42193349*	0.65076323	3.31740232	0.02064049	0.26745156	0.31157124	0.36263333
29	44,172,614	*ss117965984*	0.23199661	3.28778318	1.02199034	2.44002739	0.05228876	0.36745166
29	44,196,154	*ss86318958*	1.81161647	1.75877838	2.16745327	0.19969184	0.01874044	1.57245625
29	44,208,978	*rs29003633*	1.87290255	3.00026737	0.70748339	0.12248651	0.01088652	1.10235697
29	44,223,148	*ss86323783*	1.54925700	0.56620010	2.55633328	0.59153503	0.02959121	1.37000248
29	44,243,444	*ss86313099*	1.65698290	0.89625423	2.40467619	0.64371127	0.20064084	0.76500188
29	44,325,408	*ss86292140*	6.82799864	1.97015854	3.23951326	2.38246215	0.32381463	0.80251350
29	44,372,611	*ss86329852*	1.44683209	0.19968322	2.94240217	0.12341834	0.54213787	0.50892430
29	44,395,075	*ss86333994*	1.43940004	0.57500362	2.98266063	0.19138583	0.46385971	0.49592585
29	44,416,282	*rs43709648*	1.56049570	2.54501513	1.06492009	0.63239930	0.01669722	0.90088312
29	44,546,564	*ss86288185*	5.32381518	3.01422973	3.84893761	0.53942642	0.00462038	1.36466524
29	44,585,782	*ss86341172*	2.46757085	1.31429916	0.75921234	0.64833951	0.01279982	0.53388457
29	44,628,137	*rs41600300*	0.59487472	2.14009510	0.49302374	0.15268886	0.55836508	0.91422243
29	44,649,908	*ss86337530*	1.01897824	1.15277044	0.96862457	1.32901788	0.30646495	1.12382422
29	44,740,917	*ss86334185*	0.48680305	2.69382778	0.26618189	0.25735165	0.12919985	0.70970398
29	44,807,928	*ss117965824*	2.15392065	3.50921540	0.05121356	2.63424357	0.08061088	0.55089100
29	44,853,970	*rs42192429*	2.31724705	5.76063374	0.77973760	0.17127986	0.80095841	0.88638095
29	44,900,940	*rs43706228*	2.19610799	3.93534502	1.11862283	0.39006681	0.76834606	0.28628755
29	44,969,518	*ss86337154*	1.94685789	5.32150326	0.59049892	1.21396124	0.34880684	0.04453270
29	44,979,377	*ss86338214*	0.62499693	1.76815948	0.83021256	0.35757153	0.24917828	0.37576807
29	44,999,264	*ss86310727*	0.58311707	0.05002567	0.40984010	1.24270857	0.16974211	0.20788783
29	45,023,665	*ss86338763*	1.10742450	2.82842226	0.54096063	3.35054933	0.06487693	0.47992127
29	45,102,557	*ss86332221*	0.70729646	1.30554383	0.40632859	3.33952068	0.20370789	0.65698242
29	45,129,099	*ss86335405*	0.08011029	0.56628152	0.17913265	1.47136413	0.10457292	0.03010530
29	45,187,114	*ss86295760*	3.90874839	0.47612537	3.19993420	1.36504297	0.10578806	0.13928507
29	45,287,502	*ss86312485*	1.62763739	2.77694631	2.25493961	0.77715979	0.15892100	0.81196521
29	45,326,585	*rs42198683*	1.84232573	2.43676900	2.15095695	1.04701284	0.35084659	0.49732541
29	45,367,095	*ss86322638*						
29	45,458,280	*rs43706176*						
29	45,482,143	*rs29025626*						
29	45,530,264	*ss86341375*						

[1]UMD3.1 Coordinates. SNPs located within *CAST* and *CAPN1* are shaded in yellow.

[2]Commercialized SNPs are shaded in red.

[3]GBLUP allele substitution effect (α) ranks. Values less than 500 are shaded in yellow for the across-breed and green for the with

[4]GBLUP of SNP allele substitution effects (α) estimated as a random effect.

[5]SNP allele substitution effects (β) estimated as fixed effects in a mixed linear model including the genomic relationship matrix.

[6]$-\log_{10}$(P)-value for the test of the null hypothesis of an allele substitution effect (β) of zero. Maximum values are shaded in red. (

[7]$-\log_{10}$(P)-value for the test of the null hypothesis of all haplotype substitution effects (H) being zero. Maximum values are shade

[8]Number of haplotypes and effective number of haplotypes in parentheses for the 13 (*CAST*) or 31 (*CAPN1*) SNP block centered

[9]Number Percentage of phenotypic variation in WBSF explained by haplotypes centered on the identified SNP.

						No. Haplotypes[8]		
BTA	POS[1]	SNP ID[2]	All Breeds	Angus	Hereford	Charolais	Limousin	Simmental
7	97,151,592	*rs41656985*						
7	97,261,052	*rs41656987*						
7	97,372,656	*rs41570922*						
7	97,393,157	*rs41615915*						
7	97,430,691	*ss86295638*	8 (2.97)	8 (2.20)	6 (2.65)	8 (3.79)	7 (3.73)	8 (2.67)
7	97,501,859	*ss86310801*	8 (3.65)	8 (3.13)	6 (2.76)	8 (4.47)	6 (4.83)	7 (3.52)
7	97,529,872	*ss61472319*	8 (3.73)	8 (3.05)	7 (3.00)	7 (3.84)	7 (4.44)	8 (3.78)
7	97,691,853	*ss86290016*	7 (3.89)	7 (3.07)	6 (2.99)	7 (4.04)	7 (4.55)	7 (4.19)
7	97,772,240	*ss86312419*	7 (3.57)	7 (2.77)	7 (2.85)	7 (4.57)	7 (4.61)	7 (3.72)
7	97,861,341	*rs41593284*	7 (3.04)	7 (2.07)	6 (2.42)	7 (3.99)	7 (5.29)	7 (3.18)
7	97,898,940	*rs41593281*	6 (3.46)	6 (2.90)	6 (2.45)	6 (4.16)	6 (4.23)	6 (4.06)
7	97,961,659	*ss117969141*	6 (3.22)	6 (3.22)	5 (1.95)	6 (3.62)	6 (3.74)	6 (4.00)
7	98,013,150	*ss117968909*	7 (3.41)	6 (3.31)	5 (2.07)	7 (4.01)	6 (3.86)	6 (4.13)
7	98,173,299	*ss86290989*	8 (4.77)	6 (3.82)	7 (3.56)	7 (4.07)	6 (4.22)	8 (4.02)
7	98,194,828	*rs41658549*	8 (4.43)	6 (3.27)	7 (3.54)	6 (3.95)	6 (3.97)	8 (3.53)
7	98,254,815	*rs41658542*	8 (4.34)	7 (3.52)	7 (3.42)	6 (4.03)	7 (4.29)	8 (3.65)
7	98,375,640	*ss86329349*	8 (4.18)	7 (3.20)	7 (3.63)	7 (3.42)	7 (3.84)	8 (3.60)
7	98,495,888	*rs43529872*	9 (4.21)	8 (3.39)	8 (3.34)	7 (3.38)	8 (3.95)	9 (3.46)
7	98,498,047	*ss86321563*	9 (5.03)	8 (3.93)	8 (3.79)	8 (4.01)	8 (4.51)	9 (3.92)
7	98,538,952	*rs41660170*	8 (4.20)	8 (3.58)	7 (2.97)	8 (3.90)	7 (3.46)	8 (4.00)
7	98,566,391	*ss117962527*	8 (3.78)	8 (3.22)	6 (2.79)	7 (3.61)	6 (3.01)	7 (4.49)
7	98,579,574	*rs41255587*	8 (4.10)	8 (2.92)	6 (3.20)	7 (3.98)	6 (3.23)	7 (4.40)
7	98,635,208	*ss86300200*	8 (3.25)	8 (3.00)	6 (2.70)	7 (3.35)	6 (2.89)	7 (3.34)
7	98,646,770	*rs41596489*	9 (3.46)	9 (3.75)	6 (2.50)	7 (4.01)	6 (3.07)	7 (3.17)
7	98,680,293	*ss86315737*	8 (3.54)	8 (3.48)	6 (2.82)	7 (4.08)	7 (3.31)	7 (3.38)
7	98,726,055	*rs29018251*	8 (3.54)	7 (3.63)	7 (2.66)	7 (3.66)	7 (3.73)	8 (3.41)
7	98,757,265	*ss86303738*	7 (3.38)	6 (4.02)	5 (2.52)	7 (3.81)	6 (3.12)	7 (3.29)
7	98,820,742	*ss86324113*	7 (4.01)	7 (3.97)	7 (2.93)	6 (3.86)	6 (3.56)	7 (3.45)
7	98,887,313	*rs41568595*	8 (4.84)	7 (4.79)	7 (4.12)	7 (4.98)	7 (3.89)	8 (4.53)
7	98,907,404	*ss86291843*	7 (4.35)	6 (3.78)	6 (3.51)	7 (4.39)	7 (3.50)	7 (4.70)
7	98,974,456	*ss86339659*	7 (3.79)	7 (3.93)	7 (3.15)	7 (4.04)	7 (2.91)	7 (3.89)
7	99,024,444	*ss117968912*	7 (3.58)	7 (3.19)	6 (3.30)	7 (3.37)	6 (3.41)	6 (3.47)
7	99,085,299	*rs41590403*	7 (3.97)	6 (3.54)	6 (3.77)	6 (3.61)	6 (3.06)	7 (4.21)
7	99,130,598	*ss86333606*	7 (3.89)	6 (3.51)	6 (3.62)	6 (3.70)	6 (3.64)	7 (4.08)
7	99,178,748	*rs41568774*	9 (4.42)	7 (4.37)	6 (3.60)	6 (3.92)	6 (3.95)	8 (4.52)
7	99,215,474	*ss86324013*	9 (4.14)	7 (4.41)	8 (3.25)	7 (4.05)	7 (3.59)	9 (4.71)
7	99,344,820	*rs41622872*	8 (3.77)	6 (4.34)	8 (2.69)	7 (3.64)	7 (3.45)	8 (4.05)
7	99,399,852	*ss117968895*	9 (3.71)	7 (3.58)	8 (3.08)	6 (3.31)	7 (3.36)	7 (4.11)
7	99,492,904	*ss86296856*	8 (4.87)	7 (4.59)	7 (3.06)	6 (4.06)	7 (3.43)	7 (4.87)
7	99,635,841	*ss86339853*	9 (4.28)	7 (4.79)	7 (2.39)	7 (4.52)	8 (3.65)	7 (4.39)
7	99,687,614	*rs41657025*						
7	99,845,133	*rs41624339*						
7	99,919,908	*ss86339607*						
7	100,015,468	*ss86310226*						
29	42,406,544	*ss86319674*						
29	42,455,680	*ss117974470*						
29	42,620,218	*rs42189770*						
29	42,651,294	*rs43706142*						
29	42,696,595	*rs42189112*	7 (3.96)	7 (3.83)	7 (3.14)	7 (3.75)	7 (4.05)	7 (3.91)
29	42,749,808	*rs29020063*	8 (5.15)	8 (4.64)	8 (4.10)	8 (4.93)	8 (4.67)	8 (5.59)
29	42,842,353	*ss117974486*	9 (4.30)	8 (3.29)	9 (4.15)	8 (3.99)	8 (4.62)	9 (4.81)
29	42,897,144	*ss86322358*	9 (4.91)	9 (3.94)	9 (3.93)	9 (4.52)	9 (5.42)	9 (4.80)

29	43,006,000	*ss86339964*	10 (3.56)	9 (3.28)	9 (3.08)	9 (2.93)	9 (5.55)	10 (4.31)
29	43,043,207	*ss86341572*	9 (3.80)	8 (3.87)	8 (2.50)	8 (3.66)	8 (4.67)	9 (4.61)
29	43,108,445	*ss86337140*	10 (2.88)	7 (2.57)	7 (2.26)	7 (2.72)	7 (4.72)	10 (3.45)
29	43,129,250	*ss86337549*	9 (2.36)	7 (2.21)	7 (1.92)	7 (2.38)	6 (3.65)	9 (2.67)
29	43,189,370	*ss86319044*	8 (2.25)	7 (2.21)	7 (1.63)	7 (2.50)	7 (3.07)	8 (3.07)
29	43,224,753	*rs29013208*	8 (2.92)	6 (2.71)	7 (2.68)	7 (2.61)	6 (3.06)	8 (3.35)
29	43,269,744	*ss86282762*	7 (2.83)	5 (2.57)	6 (2.86)	6 (2.66)	6 (1.80)	7 (3.23)
29	43,304,256	*rs29024708*	7 (3.26)	5 (3.05)	7 (3.04)	6 (3.16)	5 (2.25)	7 (3.62)
29	43,328,607	*ss86295061*	7 (4.00)	7 (3.65)	5 (3.71)	6 (4.22)	5 (3.17)	7 (3.83)
29	43,429,511	*ss86318094*	8 (4.72)	8 (4.52)	6 (3.85)	7 (5.13)	6 (3.17)	8 (4.74)
29	43,466,342	*ss86310741*	8 (5.38)	7 (4.56)	7 (5.75)	6 (5.46)	7 (3.18)	8 (5.07)
29	43,498,073	*ss86338800*	8 (6.21)	8 (6.12)	8 (6.03)	7 (6.12)	7 (3.37)	8 (6.18)
29	43,611,640	*ss86298040*	9 (6.18)	8 (5.89)	8 (5.38)	9 (6.26)	7 (3.47)	8 (6.39)
29	43,652,252	*ss86327310*	7 (5.32)	7 (6.32)	7 (3.74)	7 (5.34)	7 (3.61)	7 (6.21)
29	43,686,401	*ss86325323*	7 (5.06)	6 (4.83)	6 (4.88)	7 (5.03)	6 (4.20)	6 (4.67)
29	43,709,769	*ss86290081*	7 (4.64)	6 (4.69)	6 (3.82)	7 (4.94)	6 (4.33)	6 (4.83)
29	43,747,765	*rs42190085*	7 (4.82)	7 (5.00)	7 (2.85)	7 (5.67)	7 (4.82)	7 (5.78)
29	43,749,527	*ss86299346*	7 (4.47)	6 (5.42)	6 (2.75)	7 (5.42)	7 (3.53)	7 (5.49)
29	43,777,249	*ss86299989*	6 (4.53)	6 (4.94)	5 (3.06)	6 (4.70)	6 (3.13)	6 (4.50)
29	43,826,144	*rs42191289*	6 (4.42)	6 (4.50)	6 (4.17)	6 (3.93)	6 (3.55)	6 (4.53)
29	43,839,783	*ss86336460*	7 (4.67)	6 (4.35)	7 (4.21)	6 (4.43)	6 (3.70)	7 (4.60)
29	43,914,923	*ss86335514*	8 (4.57)	7 (4.10)	7 (3.53)	7 (4.69)	6 (4.87)	8 (4.86)
29	44,000,728	*rs42191319*	8 (4.52)	7 (4.03)	7 (4.33)	6 (4.39)	6 (4.59)	6 (4.30)
29	44,029,526	*rs42192038*	7 (3.76)	7 (3.19)	7 (3.53)	7 (3.70)	6 (4.11)	7 (3.44)
29	44,042,363	*rs42192064*	7 (3.22)	6 (2.55)	6 (2.95)	5 (3.11)	5 (4.09)	5 (3.43)
29	44,049,244	*rs42192077*	6 (3.30)	5 (2.98)	6 (3.08)	4 (3.34)	4 (3.04)	5 (3.32)
29	44,050,471	*ss86284058*	6 (3.66)	6 (3.10)	6 (3.14)	6 (3.50)	6 (2.36)	6 (3.44)
29	44,051,567	*rs42192079*	6 (3.10)	6 (2.97)	5 (2.85)	5 (3.26)	4 (2.30)	6 (2.95)
29	44,053,733	*rs42192083*	6 (3.14)	6 (2.88)	5 (2.91)	5 (3.30)	4 (3.03)	6 (3.17)
29	44,054,901	*rs42192084*	6 (2.71)	6 (2.42)	5 (2.46)	5 (2.88)	4 (2.29)	6 (2.92)
29	44,058,688	*rs42192090*	5 (2.17)	5 (2.64)	5 (1.68)	5 (2.39)	4 (1.91)	4 (2.24)
29	44,060,410	*rs42192096*	6 (2.07)	4 (2.28)	5 (1.51)	6 (2.39)	5 (2.04)	5 (2.70)
29	44,062,694	*rs42192100*	6 (1.87)	5 (1.62)	6 (1.67)	5 (2.21)	5 (1.63)	6 (2.45)
29	44,063,938	*rs17872078*	7 (3.06)	5 (3.19)	7 (2.19)	5 (3.33)	6 (2.84)	6 (3.61)
29	44,067,234	*rs17872093*	7 (3.22)	6 (2.81)	7 (2.57)	6 (3.80)	6 (2.67)	6 (3.97)
29	44,067,796	*rs17871984*	8 (3.00)	7 (2.79)	8 (2.45)	7 (3.31)	7 (2.14)	7 (4.12)
29	44,068,143	*rs17870631*	9 (2.95)	7 (2.76)	8 (2.45)	7 (3.27)	6 (2.11)	8 (3.78)
29	44,068,346	*rs17870628*	9 (2.09)	7 (2.44)	7 (1.58)	6 (2.29)	5 (1.68)	8 (2.57)
29	44,068,445	*rs17870626*	7 (1.79)	7 (2.34)	6 (1.47)	6 (1.80)	4 (1.56)	6 (1.88)
29	44,068,519	*rs17872010*	7 (2.87)	6 (2.98)	6 (2.39)	7 (3.04)	6 (2.14)	6 (3.35)
29	44,068,580	*rs17872004*	6 (1.85)	6 (2.27)	6 (1.54)	5 (1.78)	6 (1.66)	5 (2.04)
29	44,068,812	*rs17872003*	6 (1.42)	5 (1.52)	6 (1.41)	5 (1.35)	6 (1.28)	6 (1.51)
29	44,069,063	*rs17812000*	7 (1.81)	5 (2.37)	6 (1.55)	5 (1.68)	5 (1.48)	7 (1.94)
29	44,069,177	*rs17872006*	6 (2.40)	5 (2.81)	6 (2.38)	5 (2.40)	6 (1.78)	6 (1.98)
29	44,070,713	*rs42192103*	8 (3.14)	7 (3.26)	7 (2.99)	7 (3.00)	8 (2.16)	8 (2.99)
29	44,070,881	*rs42192105*	9 (4.11)	8 (3.75)	8 (3.22)	8 (4.32)	8 (3.14)	9 (4.12)
29	44,071,034	*rs42192107*	9 (5.26)	8 (4.70)	8 (3.85)	8 (5.55)	8 (4.26)	9 (5.45)
29	44,071,135	*rs42192108*	9 (4.51)	8 (4.05)	7 (3.83)	7 (3.50)	9 (4.44)	8 (4.09)
29	44,071,593	*rs42192109*	8 (3.86)	8 (3.84)	6 (3.54)	7 (3.19)	8 (2.63)	7 (3.44)
29	44,085,642	*rs17871051*	8 (3.97)	8 (3.91)	6 (3.27)	6 (3.65)	7 (3.31)	7 (4.06)
29	44,085,769	*rs17871058*	7 (4.11)	7 (3.61)	6 (3.52)	6 (3.60)	6 (3.90)	6 (4.06)
29	44,087,205	*rs17872032*	7 (2.73)	6 (2.16)	5 (2.41)	6 (2.80)	6 (2.49)	6 (3.11)
29	44,087,356	*rs17872033*	7 (3.33)	7 (2.69)	6 (3.23)	6 (3.11)	6 (3.69)	7 (3.57)
29	44,087,629	*rs17872050*	7 (3.16)	6 (3.00)	6 (2.79)	6 (3.32)	5 (3.73)	7 (3.14)
29	44,087,858	*rs17872151*	7 (2.86)	7 (2.96)	6 (2.69)	6 (2.88)	5 (3.20)	7 (2.69)

29	44,088,897	*rs17870847*	7 (3.41)	6 (2.81)	6 (3.30)	6 (3.61)	6 (3.93)	7 (3.11)
29	44,110,295	*rs42192119*	6 (3.29)	6 (2.70)	5 (2.96)	5 (3.80)	5 (3.73)	6 (3.14)
29	44,119,671	*rs42195143*	6 (2.73)	5 (2.50)	5 (2.91)	5 (2.66)	5 (3.19)	6 (2.33)
29	44,122,272	*rs42194216*	6 (3.40)	5 (2.63)	6 (3.39)	6 (3.36)	6 (3.65)	6 (3.20)
29	44,125,747	*rs42194178*	6 (3.53)	6 (2.80)	6 (3.64)	6 (3.54)	6 (3.56)	6 (3.22)
29	44,130,887	*rs42194132*	7 (3.30)	5 (2.59)	5 (3.65)	6 (3.38)	5 (3.42)	7 (3.05)
29	44,154,126	*rs42193349*	7 (3.43)	6 (2.85)	6 (3.07)	7 (3.84)	6 (3.38)	7 (3.97)
29	44,172,614	*ss117965984*	7 (3.38)	5 (2.73)	5 (3.23)	7 (3.34)	6 (2.57)	6 (3.61)
29	44,196,154	*ss86318958*	7 (3.43)	5 (3.27)	5 (3.29)	6 (3.29)	6 (2.81)	6 (3.56)
29	44,208,978	*rs29003633*	8 (3.58)	6 (3.65)	6 (3.21)	7 (3.24)	7 (3.19)	6 (3.63)
29	44,223,148	*ss86323783*	8 (3.87)	7 (3.61)	7 (2.87)	7 (3.90)	8 (4.32)	7 (4.22)
29	44,243,444	*ss86313099*	8 (4.51)	8 (3.85)	8 (3.91)	8 (3.93)	8 (4.86)	8 (4.48)
29	44,325,408	*ss86292140*	8 (3.88)	8 (2.40)	8 (3.56)	8 (4.31)	8 (4.87)	8 (4.02)
29	44,372,611	*ss86329852*	8 (3.84)	7 (3.23)	8 (3.51)	7 (4.19)	7 (4.60)	7 (3.80)
29	44,395,075	*ss86333994*	8 (3.87)	7 (3.25)	8 (3.61)	7 (4.23)	7 (4.71)	7 (3.86)
29	44,416,282	*rs43709648*	8 (4.31)	7 (3.21)	8 (3.88)	8 (5.07)	7 (4.95)	8 (4.50)
29	44,546,564	*ss86288185*	8 (4.92)	7 (4.21)	7 (4.75)	8 (5.14)	8 (4.49)	8 (4.64)
29	44,585,782	*ss86341172*	9 (3.72)	8 (2.79)	8 (4.09)	8 (3.58)	9 (4.63)	9 (3.25)
29	44,628,137	*rs41600300*	9 (4.29)	8 (3.15)	8 (4.14)	8 (3.57)	9 (4.53)	9 (3.86)
29	44,649,908	*ss86337530*	8 (4.23)	6 (3.25)	8 (3.98)	7 (4.06)	7 (4.28)	8 (3.60)
29	44,740,917	*ss86334185*	8 (3.53)	7 (3.16)	7 (2.90)	7 (3.23)	7 (3.91)	8 (3.46)
29	44,807,928	*ss117965824*	8 (3.56)	8 (2.83)	7 (2.99)	7 (3.54)	7 (4.67)	8 (4.23)
29	44,853,970	*rs42192429*	7 (4.05)	7 (4.15)	7 (3.07)	7 (4.22)	7 (4.08)	7 (4.47)
29	44,900,940	*rs43706228*	8 (4.12)	8 (4.04)	7 (3.13)	8 (4.32)	7 (4.58)	8 (4.25)
29	44,969,518	*ss86337154*	8 (4.45)	8 (4.24)	8 (3.82)	8 (4.46)	7 (4.60)	8 (4.57)
29	44,979,377	*ss86338214*	7 (4.14)	7 (3.79)	7 (4.21)	7 (3.71)	6 (4.61)	7 (4.08)
29	44,999,264	*ss86310727*	7 (3.92)	7 (3.51)	7 (2.74)	6 (3.27)	6 (3.90)	7 (4.34)
29	45,023,665	*ss86338763*	7 (4.03)	6 (3.94)	7 (3.90)	6 (3.19)	6 (4.38)	7 (4.21)
29	45,102,557	*ss86332221*	7 (3.53)	7 (3.34)	7 (2.88)	7 (3.65)	7 (4.83)	7 (3.92)
29	45,129,099	*ss86335405*	7 (4.16)	7 (4.25)	6 (3.72)	6 (4.41)	6 (3.77)	7 (4.25)
29	45,187,114	*ss86295760*	6 (4.30)	6 (3.97)	6 (3.68)	6 (4.83)	5 (3.52)	6 (4.10)
29	45,287,502	*ss86312485*	6 (4.45)	6 (3.35)	6 (3.93)	6 (4.76)	6 (3.23)	6 (4.87)
29	45,326,585	*rs42198683*	7 (4.08)	6 (3.24)	5 (3.51)	6 (4.34)	5 (3.09)	6 (4.52)
29	45,367,095	*ss86322638*						
29	45,458,280	*rs43706176*						
29	45,482,143	*rs29025626*						
29	45,530,264	*ss86341375*						

[1]UMD3.1 Coordinates. SNPs located within *CAST* and *CAPN1* are shaded in yellow.

[2]Commercialized SNPs are shaded in red.

[3]GBLUP allele substitution effect (α) ranks. Values less than 500 are shaded in yellow for the across-breed and gree

[4]GBLUP of SNP allele substitution effects (α) estimated as a random effect.

[5]SNP allele substitution effects (β) estimated as fixed effects in a mixed linear model including the genomic relation

[6]$-\log_{10}$(P)-value for the test of the null hypothesis of an allele substitution effect (β) of zero. Maximum values are sh

[7]$-\log_{10}$(P)-value for the test of the null hypothesis of all haplotype substitution effects (H) being zero. Maximum val

[8]Number of haplotypes and effective number of haplotypes in parentheses for the 13 (*CAST*) or 31 (*CAPN1*) SNP bl

[9]Number Percentage of phenotypic variation in WBSF explained by haplotypes centered on the identified SNP.

						% V_P explained[9]				
BTA	POS[1]	SNP ID[2]	All Breeds	Angus	Hereford	Charolais	Limousin	Simmental		
7	97,151,592	*rs41656985*								
7	97,261,052	*rs41656987*								
7	97,372,656	*rs41570922*								
7	97,393,157	*rs41615915*								
7	97,430,691	*ss86295638*	0.1069	0.3348	0.3181	0.2968	2.9028	0.5612		
7	97,501,859	*ss86310801*	0.1916	0.2039	0.3081	0.2291	3.8762	0.5472		
7	97,529,872	*ss61472319*	0.3281	0.2727	0.8707	0.2474	3.0739	0.8633		
7	97,691,853	*ss86290016*	0.2342	0.1155	0.7879	0.1839	2.7549	0.1346		
7	97,772,240	*ss86312419*	0.2042	0.1218	0.5156	0.0972	3.3667	1.3058		
7	97,861,341	*rs41593284*	0.1991	0.2543	0.8272	0.8832	4.0170	1.5665		
7	97,898,940	*rs41593281*	0.1880	0.2159	0.3170	0.4538	2.0289	1.6484		
7	97,961,659	*ss117969141*	0.2257	0.3903	0.5856	0.5680	2.9404	1.3240		
7	98,013,150	*ss117968909*	0.2944	0.3231	0.7719	0.7887	3.0408	1.1337		
7	98,173,299	*ss86290989*	0.6082	0.2174	1.5111	0.5415	1.2811	2.2421		
7	98,194,828	*rs41658549*	0.7887	0.7155	1.5810	0.6017	2.1868	2.0381		
7	98,254,815	*rs41658542*	0.8412	0.7193	1.7833	0.4899	2.8748	2.7163		
7	98,375,640	*ss86329349*	0.6480	0.5146	1.7002	0.3179	2.6146	2.7748		
7	98,495,888	*rs43529872*	1.0168	1.3608	1.9218	1.8626	3.2645	1.9619		
7	98,498,047	*ss86321563*	0.6907	0.6996	1.6994	0.9652	2.2145	1.7102		
7	98,538,952	*rs41660170*	0.6994	0.8205	1.8942	2.0957	1.4259	2.4906		
7	98,566,391	*ss117962527*	0.6561	0.6368	1.8793	1.3322	2.1184	0.8685		
7	98,579,574	*rs41255587*	0.5320	0.5029	1.5045	1.1954	1.7032	1.1394		
7	98,635,208	*ss86300200*	0.3645	0.4199	1.3981	0.9644	3.6388	0.3863		
7	98,646,770	*rs41596489*	0.5032	0.3066	1.1240	0.6743	1.6654	1.6151		
7	98,680,293	*ss86315737*	0.2711	0.4693	0.6606	0.6184	3.4188	1.6557		
7	98,726,055	*rs29018251*	0.4022	0.0532	1.3334	1.0384	0.5667	2.4458		
7	98,757,265	*ss86303738*	0.0872	0.0489	0.5442	0.2399	0.4469	1.3233		
7	98,820,742	*ss86324113*	0.2221	0.0468	0.6508	0.5775	0.7773	0.4901		
7	98,887,313	*rs41568595*	0.2211	0.2870	1.0798	1.2957	1.2288	1.2300		
7	98,907,404	*ss86291843*	0.0623	0.0927	0.3782	0.7934	1.3305	0.7421		
7	98,974,456	*ss86339659*	0.1047	0.2045	0.4300	1.6508	3.5200	0.7290		
7	99,024,444	*ss117968912*	0.2343	0.4070	1.1508	1.8810	1.1603	0.2613		
7	99,085,299	*rs41590403*	0.2377	0.2211	1.0249	0.7492	0.9754	0.6307		
7	99,130,598	*ss86333606*	0.1205	0.0478	0.6514	0.5958	2.2676	0.3095		
7	99,178,748	*rs41568774*	0.1257	0.1654	0.3425	0.6688	0.7915	0.6747		
7	99,215,474	*ss86324013*	0.3063	0.2844	1.0966	0.4720	0.3325	1.1453		
7	99,344,820	*rs41622872*	0.1857	0.5936	0.6192	0.1349	1.6586	1.4045		
7	99,399,852	*ss117968895*	0.0522	0.0772	0.2063	0.0734	1.9788	0.1781		
7	99,492,904	*ss86296856*	0.1716	0.1178	0.3100	0.3185	1.8695	0.8208		
7	99,635,841	*ss86339853*	0.1240	0.1541	0.1788	0.3416	3.1080	0.8480		
7	99,687,614	*rs41657025*								
7	99,845,133	*rs41624339*								
7	99,919,908	*ss86339607*								
7	100,015,468	*ss86310226*								
29	42,406,544	*ss86319674*								
29	42,455,680	*ss117974470*								
29	42,620,218	*rs42189770*								
29	42,651,294	*rs43706142*								
29	42,696,595	*rs42189112*	0.0971	0.5927	0.1067	0.4390	0.9426	0.8560		
29	42,749,808	*rs29020063*	0.1618	0.7164	0.1655	0.6353	1.6734	2.4237		
29	42,842,353	*ss117974486*	0.2566	0.5694	0.6020	0.1532	0.9007	2.5806		
29	42,897,144	*ss86322358*	0.1998	1.2397	0.6377	0.3813	1.5694	2.2685		

29	43,006,000	*ss86339964*	0.2243	1.3464	0.5149	0.3115	2.8678	3.3851		
29	43,043,207	*ss86341572*	0.2150	1.2177	0.2691	0.2727	1.1606	2.9719		
29	43,108,445	*ss86337140*	0.2371	1.1154	0.0496	0.0485	1.0267	3.5414		
29	43,129,250	*ss86337549*	0.3180	1.7102	0.1659	0.2836	0.3402	2.3409		
29	43,189,370	*ss86319044*	0.2375	0.7152	0.6429	0.0879	1.1739	1.4806		
29	43,224,753	*rs29013208*	0.3705	1.2772	0.9855	0.0883	0.6477	1.6622		
29	43,269,744	*ss86282762*	0.4843	1.3414	1.0922	0.2052	1.5560	1.8456		
29	43,304,256	*rs29024708*	0.2190	0.6515	0.6304	0.2211	0.5122	2.3447		
29	43,328,607	*ss86295061*	0.2609	1.1412	0.2814	0.9226	0.1729	2.3193		
29	43,429,511	*ss86318094*	0.3438	1.7052	0.7278	0.7317	0.3052	2.7134		
29	43,466,342	*ss86310741*	0.1778	0.6858	0.6546	0.3277	0.1834	1.5918		
29	43,498,073	*ss86338800*	0.2699	1.4302	0.7505	0.6832	1.5707	1.1878		
29	43,611,640	*ss86298040*	0.3270	0.9183	0.6246	0.7037	1.9166	1.9407		
29	43,652,252	*ss86327310*	0.2851	1.2446	0.4059	0.8527	1.6756	2.0019		
29	43,686,401	*ss86325323*	0.3868	1.9641	0.5687	0.7995	0.1161	2.7630		
29	43,709,769	*ss86290081*	0.3391	2.4762	0.4812	0.7101	1.9074	3.2671		
29	43,747,765	*rs42190085*	0.2557	1.1582	0.6285	0.2086	2.1066	3.2947		
29	43,749,527	*ss86299346*	0.2107	0.5996	0.5609	0.5481	2.1140	2.3776		
29	43,777,249	*ss86299989*	0.3691	0.6800	0.7725	0.3275	2.9688	2.3050		
29	43,826,144	*rs42191289*	0.5797	0.6216	1.4613	0.5674	0.6505	2.0858		
29	43,839,783	*ss86336460*	0.1034	0.6447	0.6530	0.2815	0.6028	1.1670		
29	43,914,923	*ss86335514*	0.1333	0.6012	0.2374	0.5500	0.7245	2.0326		
29	44,000,728	*rs42191319*	0.1921	0.1353	0.4667	0.5837	0.6951	1.4871		
29	44,029,526	*rs42192038*	0.1275	0.3279	0.3417	0.6893	1.0148	1.1975		
29	44,042,363	*rs42192064*	0.0646	0.1550	0.4965	0.4318	0.1630	0.2726		
29	44,049,244	*rs42192077*	0.2041	0.5438	0.5527	0.6122	0.0954	1.0924		
29	44,050,471	*ss86284058*	0.2310	0.7920	0.4733	0.4115	1.0465	2.6991		
29	44,051,567	*rs42192079*	0.3953	0.7053	1.0313	0.4786	0.0496	3.5538		
29	44,053,733	*rs42192083*	0.4032	1.0252	0.6287	0.5270	0.4525	3.6792		
29	44,054,901	*rs42192084*	1.1142	1.3904	1.9370	1.3357	0.4794	2.7195		
29	44,058,688	*rs42192090*	0.7568	0.7332	2.0917	1.4072	0.2471	2.7775		
29	44,060,410	*rs42192096*	0.0489	0.5181	0.9330	0.7528	1.0922	0.4018		
29	44,062,694	*rs42192100*	1.1801	0.9229	2.5851	0.8142	1.1448	2.3275		
29	44,063,938	*rs17872078*	1.2277	0.8836	2.4599	1.1151	0.6002	2.9079		
29	44,067,234	*rs17872093*	1.6207	2.2689	2.3735	1.5350	0.9956	5.0523		
29	44,067,796	*rs17871984*	1.8030	2.1985	2.5524	1.9766	1.2721	5.1526		
29	44,068,143	*rs17870631*	1.8519	2.3525	2.4650	1.9660	0.9285	5.3481		
29	44,068,346	*rs17870628*	1.3353	1.9642	1.3739	1.8383	0.7218	4.2853		
29	44,068,445	*rs17870626*	1.3634	3.1851	1.5340	1.8558	0.6257	3.9445		
29	44,068,519	*rs17872010*	1.4843	3.1839	2.3279	2.1726	2.6316	4.4334		
29	44,068,580	*rs17872004*	0.6819	2.0162	1.6311	0.3337	0.9731	2.2337		
29	44,068,812	*rs17872003*	0.2756	0.9369	0.7108	0.7401	0.7367	1.1522		
29	44,069,063	*rs17812000*	1.0159	2.3693	1.1396	1.2239	0.4688	4.6553		
29	44,069,177	*rs17872006*	1.2804	2.5017	1.6476	1.7458	2.0917	4.4879		
29	44,070,713	*rs42192103*	1.2167	2.3723	1.9232	1.9906	2.8836	4.3459		
29	44,070,881	*rs42192105*	1.4070	2.6580	2.2227	2.7550	3.5215	4.2918		
29	44,071,034	*rs42192107*	1.3879	2.5015	2.0222	2.6817	1.5437	4.5974		
29	44,071,135	*rs42192108*	0.4005	1.3964	0.9437	0.7492	1.7837	1.5178		
29	44,071,593	*rs42192109*	0.3764	1.2252	0.8942	0.7841	2.1647	0.6059		
29	44,085,642	*rs17871051*	0.3539	1.6836	0.2326	1.4792	2.5763	3.1650		
29	44,085,769	*rs17871058*	0.7367	2.9196	1.3546	1.4674	2.6655	2.9538		
29	44,087,205	*rs17872032*	0.7179	2.5865	0.6547	0.9348	2.7454	2.9682		
29	44,087,356	*rs17872033*	0.3732	1.1418	0.5388	0.3120	2.9878	1.9136		
29	44,087,629	*rs17872050*	0.3993	0.6251	0.6325	0.9748	1.6848	2.2658		
29	44,087,858	*rs17872151*	0.2942	0.6947	0.9837	0.3630	0.8176	1.4608		

29	44,088,897	*rs17870847*	0.0699	0.4757	0.2390	0.4133	0.2829	0.0973		
29	44,110,295	*rs42192119*	0.2301	0.5087	0.1788	0.6008	0.2157	1.7515		
29	44,119,671	*rs42195143*	0.1902	0.3015	0.5057	0.1852	0.4199	0.7790		
29	44,122,272	*rs42194216*	0.3200	0.2749	0.6289	1.3313	0.7139	1.8567		
29	44,125,747	*rs42194178*	0.2934	0.2900	0.2223	1.4256	0.5045	2.0344		
29	44,130,887	*rs42194132*	0.2816	0.2694	0.2685	0.9360	0.8166	2.1318		
29	44,154,126	*rs42193349*	0.1469	1.3176	0.0735	0.3572	1.1035	1.0763		
29	44,172,614	*ss117965984*	0.0837	1.1845	0.5237	1.3592	0.4293	0.8918		
29	44,196,154	*ss86318958*	0.2808	0.7172	0.9353	0.2450	0.2646	2.2890		
29	44,208,978	*rs29003633*	0.3164	1.2198	0.4870	0.2434	0.3088	1.7895		
29	44,223,148	*ss86323783*	0.2804	0.4551	1.3181	0.5502	0.6021	2.3560		
29	44,243,444	*ss86313099*	0.2925	0.6766	1.3772	0.6640	1.3068	1.8720		
29	44,325,408	*ss86292140*	0.7977	1.0914	1.6889	1.4586	1.6338	1.9225		
29	44,372,611	*ss86329852*	0.2686	0.2614	1.5797	0.2443	1.8282	1.2962		
29	44,395,075	*ss86333994*	0.2678	0.4591	1.5946	0.3017	1.6760	1.2776		
29	44,416,282	*rs43709648*	0.2816	1.1864	0.8254	0.6576	0.3649	2.0519		
29	44,546,564	*ss86288185*	0.6589	1.3387	1.7776	0.6043	0.3169	2.6192		
29	44,585,782	*ss86341172*	0.4103	0.8473	0.6801	0.6665	0.5738	1.7507		
29	44,628,137	*rs41600300*	0.1818	1.1516	0.5392	0.3301	2.4348	2.3067		
29	44,649,908	*ss86337530*	0.2174	0.6102	0.7810	0.9018	1.3420	2.3320		
29	44,740,917	*ss86334185*	0.1446	1.2352	0.3330	0.3502	0.8755	1.7962		
29	44,807,928	*ss117965824*	0.3468	1.6097	0.1538	1.4348	0.7054	1.5679		
29	44,853,970	*rs42192429*	0.3331	2.1742	0.6054	0.2857	2.2915	1.7928		
29	44,900,940	*rs43706228*	0.3513	1.7453	0.7563	0.5117	2.2357	1.1298		
29	44,969,518	*ss86337154*	0.3245	2.1719	0.5930	0.9527	1.4366	0.5132		
29	44,979,377	*ss86338214*	0.1435	0.9229	0.6287	0.4160	0.9712	1.0971		
29	44,999,264	*ss86310727*	0.1378	0.1383	0.4188	0.7578	0.7855	0.8074		
29	45,023,665	*ss86338763*	0.2031	1.1662	0.4889	1.5674	0.4774	1.2544		
29	45,102,557	*ss86332221*	0.1543	0.7558	0.4168	1.7026	1.0905	1.5001		
29	45,129,099	*ss86335405*	0.0505	0.4551	0.2156	0.8526	0.6079	0.3385		
29	45,187,114	*ss86295760*	0.4511	0.3444	1.4206	0.8089	0.4334	0.5176		
29	45,287,502	*ss86312485*	0.2321	1.1501	1.0889	0.5532	0.7580	1.4601		
29	45,326,585	*rs42198683*	0.2840	1.0424	0.9296	0.6741	0.9232	1.0704		
29	45,367,095	*ss86322638*								
29	45,458,280	*rs43706176*								
29	45,482,143	*rs29025626*								
29	45,530,264	*ss86341375*								

[1]UMD3.1 Coordinates. SNPs located within *CAST* and *CAPN1* are shaded in yellow.

[2]Commercialized SNPs are shaded in red.

[3]GBLUP allele substitution effect (α) ranks. Values less than 500 are shaded in yellow for the across-breed and green for the within-breed

[4]GBLUP of SNP allele substitution effects (α) estimated as a random effect.

[5]SNP allele substitution effects (β) estimated as fixed effects in a mixed linear model including the genomic relationship matrix.

[6]$-\log_{10}$(P)-value for the test of the null hypothesis of an allele substitution effect (β) of zero. Maximum values are shaded in red. Other larg

[7]$-\log_{10}$(P)-value for the test of the null hypothesis of all haplotype substitution effects (H) being zero. Maximum values are shaded in red.

[8]Number of haplotypes and effective number of haplotypes in parentheses for the 13 (*CAST*) or 31 (*CAPN1*) SNP block centered on the id

[9]Number Percentage of phenotypic variation in WBSF explained by haplotypes centered on the identified SNP.

analyses.

ge values within the region are shaded in yellow (across breed analysis) or green (within breed analyses).

Other large values within the region are shaded in yellow (across breed analysis) or green (within breed analyses).

entified SNP.
